# Cyanophages as an important factor in the early evolution of oxygenic photosynthesis

**DOI:** 10.1038/s41598-022-24795-1

**Published:** 2022-11-29

**Authors:** Ireneusz Ślesak, Halina Ślesak

**Affiliations:** 1 The Franciszek Górski Institute of Plant Physiology, Polish Academy of Sciences, Niezapominajek 21, 30-239 Kraków, Poland; 2 Institute of Botany, Faculty of Biology, Jagiellonian University, Gronostajowa 9, 30-387 Kraków, Poland

**Keywords:** Computational biology and bioinformatics, Evolution, Microbiology, Plant sciences

## Abstract

Cyanophages are viruses that infect cyanobacteria. An interesting feature of many of them is the presence of *psbA* and *psbD*, genes that encode D1 and D2 proteins, respectively. The D1 and D2 are core proteins of the photosystem II (PSII) in cyanobacteria, algae and plants and influence the proper function of oxygenic photosynthesis (OP) in all oxyphototrophs on Earth. The frequent occurrence of *psbA* and *psbD* in cyanophages raises the question whether these genes coevolved with hosts during the early stages of cyanophage and cyanobacterial evolution, or whether they are direct descendants of genes adopted from the genomes of cyanobacterial hosts. The phylogeny of D1/D2 proteins encoded in the genomes of selected cyanophages and oxyphototrophs was reconstructed. In addition, common ancestral sequences of D1 and D2 proteins were predicted for cyanophages and oxyphototrophs. Based on this, the reconstruction of the 3D structures of D1 and D2 proteins was performed. In addition, the ratio of non-synonymous to synonymous (d_N_/d_S_) nucleotide substitutions in the coding sequences (CDSs) of *psbA* and *psbD* was determined. The results of the predicted spatial structures of the D1 and D2 proteins and purifying selection for the CDSs of *psbA* and *psbD* suggest that they belong to the ancient proteins, which may have formed the primordial PSII. It cannot be ruled out that they involved in water oxidation in cyanobacteria-like organisms at early stages of the evolution of life on Earth and coevolved with ancient cyanophages. The data are also discussed in the context of the origin of viruses.

## Introduction

Cyanophages, or bacteriophages (phages) that infect cyanobacteria, were first described in the 1990s^1,2^. To date, the genomes of more than 70 cyanophages, mainly living in marine environments, have been identified and sequenced^3^. Interestingly, the vast majority of described cyanophages mainly infect cyanobacteria from the two genera *Prochlorococcus* and *Synechococcus*, which in turn are the dominant oxyphototrophs on Earth, as they inhabit all oceans^4–7^. Cyanophages are among most abundant ‘semi-life’ forms that play a crucial role in global biogeochemical cycles of carbon, oxygen and nitrogen^8,9^.

A particularly interesting feature of cyanophages is the presence of genes in their genome that encode proteins involved in the photosynthetic activity of cyanobacteria. The best known in this context are the genes coding the PSII core proteins that are most abundant in cyanophages, namely *psbA* for protein D1 and *psbD* for protein D2^3,10,11^. The first photosynthetic gene identified in cyanophages was *psbA*^12,13^. 80% of the cyanophage genomes sequenced so far have *psbA*, and 42% have both *psbA* and *psbD* genes^3^. The function of the cyanophage genes *psbA* and *psbD* is not yet fully understood, but it is likely that they play a key role in infection under changing light conditions that cause photoinhibition of host D1/D2 proteins^14^. Light-induced photoinhibition causes a more rapid degradation of D1/D2 than the completion of the cyanophage developmental cycle. Thus, providing copies of *psbA* (D1) and *psbD* (D2) during viral infection allows the infected cell to be photosynthetically active for as long as is required for full viral particle replication^3,15–17^.

The relatively wide distribution of *psbA* and *psbD* in cyanophages raises the question of whether these genes evolved in co-evolution with hosts in the early stages of cyanophage and cyanobacterial evolution or whether they are direct descendants of genes that cyanophages later adopted from the genomes of cyanobacterial hosts. The answer to this question relates to at least two interrelated issues. First, it concerns the origin of viruses in general and cyanophages in particular. Second, it sheds light on the evolution of oxygenic photosynthesis (OP) too, since cyanobacteria-like organisms are generally considered to be the first oxyphototrophs on Earth^18–20^. According to the most widely held view, the ancestor of cyanobacteria did not possess photosynthetic genes encoding proteins involved in O_2_ production. OP probably arose about 2.3–2.6 billion years ago during the so-called Great Oxidation Event (GOE) through lateral gene transfer and the fusion of two photosynthetic systems derived from primordial, non-oxygen evolving bacteria^21–23^. Some recent phylogenetic analyses suggest that OP may have arisen at very early stages of the evolution of life on Earth^24–26^. Therefore, the molecular evolution of proteins at the coding sequence (CDS) level for *psbA* and *psbD* was also studied here in the context of nucleotide substitutions in codons. Most nucleotide substitutions occur at the 3rd position in the codon, less frequently at the 1st and 2nd positions^27^. A nucleotide substitution in a codon can be synonymous and we usually find it at the 3rd position. Generally, synonymous substitutions do not lead to a change in the encoded amino acid in the protein. As a result, the protein retains its biological function. In other words, such a change is neutral and invisible to natural selection. Non-synonymous nucleotide substitutions at the codon lead to a change in the amino acid in the protein. The altered protein can in turn affect the fitness of an organism. Determining the number of non-synonymous (d_N_) and synonymous (d_S_) nucleotide substitutions therefore allows us to estimate what type of selection we are dealing with within the CDS under study^27–29^. By comparing the d_N_/d_S_ ratio, we were thus able to determine which type of selection acts on the CDS for *psbA* and *psbD* in cyanophages and selected oxyphototrophs (see "Methods" for details).

Our data are presented in the context of the various hypotheses that concern origin of viruses. It cannot be ruled out that cyanophages carrying *psbA*/*psbD* genes are ancient remnants and "living fossils" from the earliest stages of the evolution of life, when a mixture of protocyanobacterial and virus-like replicators existed.

## Results

### Phylogenetic analysis of D1 and D2 proteins

**Figure 1:**
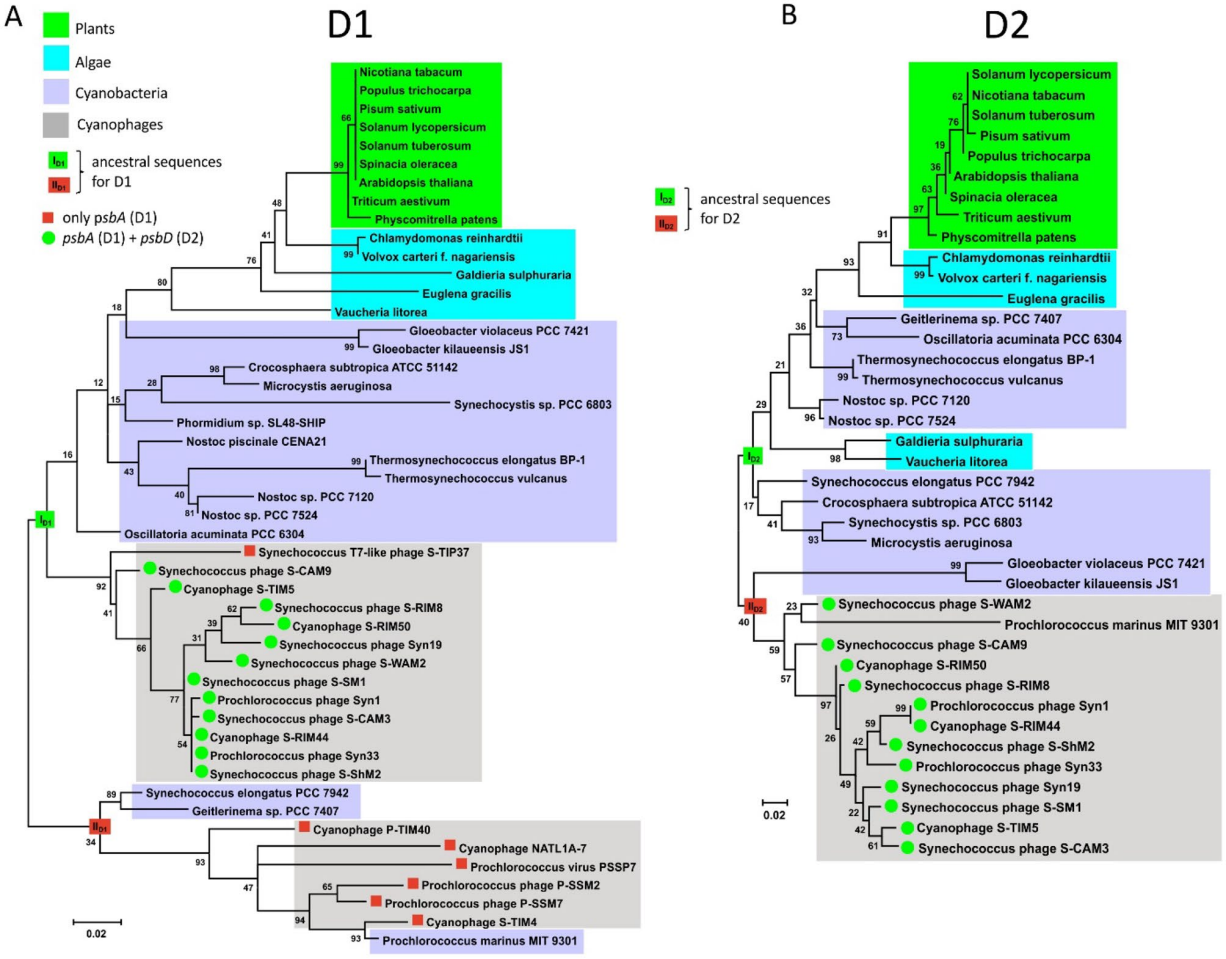
Phylogeny of the D1 and D2 proteins for the organism and cyanophage groups analysed in this study (Supplementary Information Notes S1 and Notes S2). (**A**) The unrooted phylogenetic tree of D1. (**B**) The unrooted phylogenetic tree of D2. The deepest nodes for reconstructing the ancestral sequences, designated I_D1_ and II_D1_ for D1 (**A**) and I_D2_ and II_D2_ for D2 (**B**), are indicated. The occurrence of *psbA* alone or *psbA* and *psbD* in the cyanophages is indicated. The numbers at the nodes represent bootstrap values (based on 1000 replicates). The scale shows the number of substitutions per amino acid position.

Phylogenetic trees were reconstructed for D1 and D2 proteins, highlighting cyanophages that have only the gene for the D1 protein (*psbA*) and/or D2 protein (*psbD*) in the genome. In the group of cyanophages examined, no case was found in which only *psbD* was present; when *psbD* was present, it was only together with *psbA* (Fig. 1). In contrast, in some cyanophages *psbA* was present as the only gene for the PSII core protein (Fig. 1).

The phylogenetic tree reconstructed for the D1 protein showed several distinct clades: for plants, algae and most of the cyanobacteria analysed (Fig. 1A). One of these clades, with the exception of the Synechococcus T7-like phage S-TIP37, includes cyanophages that have *psbD* for the D2 protein in their genome in addition to the *psbA* gene for D1 (Fig. 1A). This entire group of cyanophages most likely shares a common ancestor with D1 for other cyanobacteria, algae and plants.

A separate cyanophage clade is formed by cyanophages that only possess D1. Interestingly, 3 species of cyanobacteria also belong to this clade: *Synechococcus elongatus* PCC 7942, *Geitlerinema* sp. PCC 7407 and *Prochlorococcus marinus* MIT 9301 (Fig. 1). The localisation of the D1 protein in *P. marinus* suggests that *psbA* is derived from cyanophage ancestors of *psbA*. D1 in this group in turn shares a common ancestor with D1 in *S. elongatus* and *Geitlerinema* sp. (Fig. 1A).

### Phylogenetic analysis of D2 protein

In contrast to the phylogenetic tree for D1, the tree reconstructed for D2 contains fewer protein sequences. This is due to the fact that many fewer cyanophages contain *psbD*, which codes for the D2 protein, in their genome. Moreover, if *psbD* is already present in the cyanophage genome, it is accompanied by *psbA* for the D1 protein (Fig. 1B). Similar to the tree for D1, plants, algae and cyanobacteria form separate clades. However, it is worth noting that D2 occurs in two algal species: *Galdieria sulphuraria* and *Vaucheria litorea* between the D2 sequences for cyanobacteria (Fig. 1B). This result suggests that horizontal gene transfer for *psbD* may have occurred in the past between selected groups of algae and cyanobacteria. This possibility cannot be ruled out, as HGT between bacteria, cyanobacteria, viruses and algae, including the red alga *G. sulphuraria*, has already been observed^30^.

### Ancestral sequence reconstruction and prediction of spatial structure

**Figure 2:**
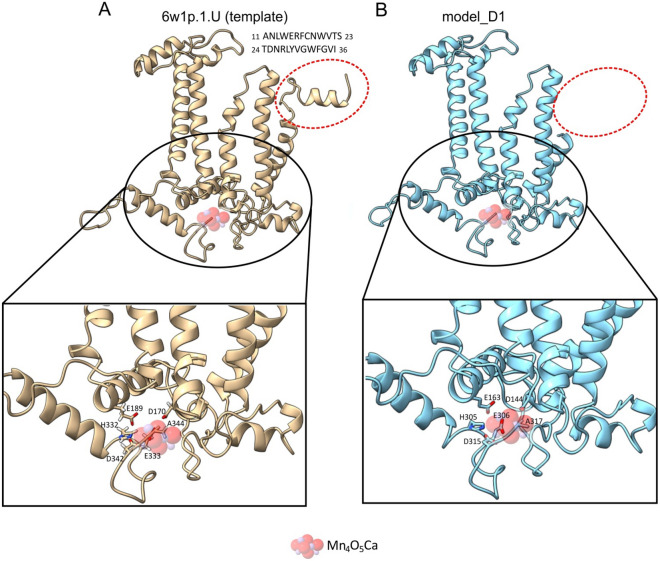
The predicted 3D structure of the ancestral D1 protein. (**A**) The structure of the template sequence from *T. elongatus* (PDB: 6w1p.1.U). (**B**) The structure of the predicted D1 protein (model_D1). Shown is the 26 amino acid sequence by which the two structures differ. The Mn_4_O_5_Ca cluster and its binding amino acid residues are shown. The 3D protein structures were visualised and analysed using UCSF ChimeraX software^31^.

The algorithm in the workspace SWISS-MODEL used the D1 template (PDB: 6w1p.1.U), which is 10 amino acids shorter at the N-terminus than the full D1 sequence (UniProt: P0A444, Supplementary Information Notes S5). Homology modelling performed by SWISS-MODEL showed that the predicted and ancestral D1 protein (model_D1) is 37 and 27 amino acids shorter than the full D1 sequence (UniProt: P0A444) and the D1 template (PDB: 6w1p.1.U) from *Thermosynechococcus elongatus*, respectively. The D1 model lacks the first 26 amino acids and is missing D308 (Fig. 2, Supplementary Information Notes S5). D1 belongs to proteins that play a key role in binding the Mn_4_O_5_Ca cluster^24,25,32^. In *T. elongatus*, the following amino acids of D1 are involved in this binding and the proper function of the oxygen evolving complex (OEC): D170, E189, H332, E333, D342, and A344^33^, https://www.rcsb.org/3d-view/6W1P, Supplementary Information Notes S5]. These 6 amino acid residues of the D1 protein are present in all modern organisms that carry out OP^34^. Although the predicted model protein is shorter than the template, namely only shifted by 37 amino acid residues, there is an identical set and pattern of amino acids required for the functioning of OEC: D144, E163, H305, E306, D315 and A317 (Fig. 2, Supplementary Information Notes S5). In general, the identity between the template and the model is ~ 83%. The results indicate that the amino acid sequence of a potentially ancestral D1 protein has not changed significantly compared to the extant D1 proteins found in oxyphototrophs and encoded in cyanophage genomes.

**Figure 3:**
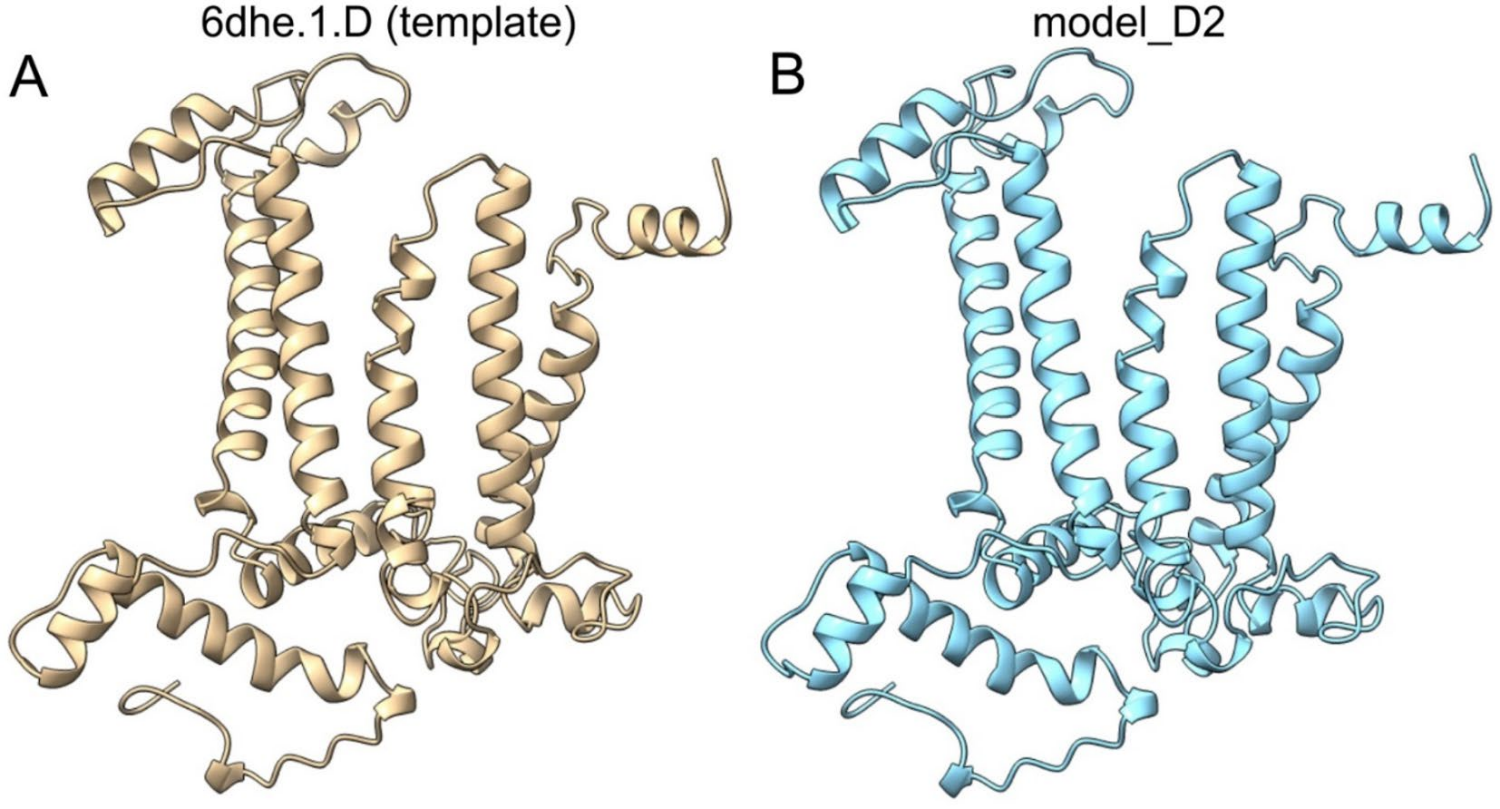
The predicted 3D structure of the ancestral D2 protein. (**A**) The structure of the template sequence from *T. elongatus* (PDB 6dhe.1.D). (**B**) The structure of the predicted D2 protein (model_D2). The 3D protein structures were visualised and analysed using UCSF ChimeraX software^31^.

In the case of the D2 protein, the template was also the D2 protein from *T. elongatus* (6dhe.1.D). We obtained a much more similar, almost identical 3D model of the predicted, probably ancestral D2 (Fig. 3, Supplementary Information Notes S6). The differences concern substitutions of only 22 amino acids with similar physicochemical properties (Supplementary Information Notes S6). Although the amino acids of the D2 protein are not directly involved in the binding of the Mn_4_O_5_Ca cluster, they play an important role in proper functioning of PSII^25^. It should be noted that the amino acid residues involved in Mn_4_O_5_Ca binding (Fig. 2) appear to be very conservative and probably occurred in an ancient D1-like protein at a very early stage of OP evolution.

### Determination of d_N_/d_S_ values

**Figure 4:**
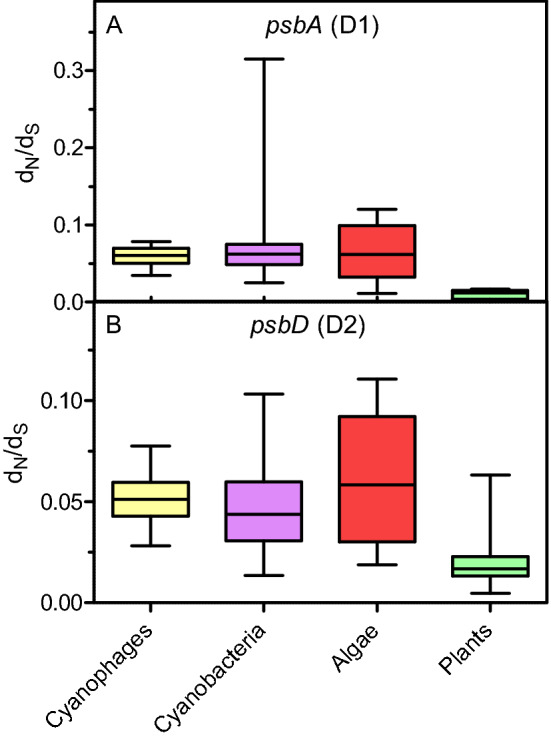
The pattern of evolutionary changes in CDSs for *psbA* (D1) and *psbD* (D2). (**A**) d_N_/d_S_ values of *psbA*. (**B**) d_N_/d_S_ values of *psbD*. Data represent min–max and median values. Values were determined within groups: cyanophages, cyanobacteria, algae and plants. See a detailed list of sequences and species (Supplementary Information Notes S7 and S8).

Analysis of CDSs for *psbA* and *psbD*, encoding D1 and D2 proteins respectively, showed that the d_N_/d_S_ values for cyanophages, cyanobacteria, algae and plants were lower than 1 (d_N_/d_S_ < 1) (Fig. 4, for details see “Methods”). The results show that the CDSs tested are subject to purifying (negative) selection. Interestingly, the d_N_/d_S_ values for *psbA* and *psbD* were much more similar in both cyanophages and cyanobacteria than the d_N_/d_S_ values in algae and plants (Fig. 4). These results suggest a close co-evolution of *psbA*/*psbD* in cyanophages and cyanobacteria.

## Discussion

**Figure 5:**
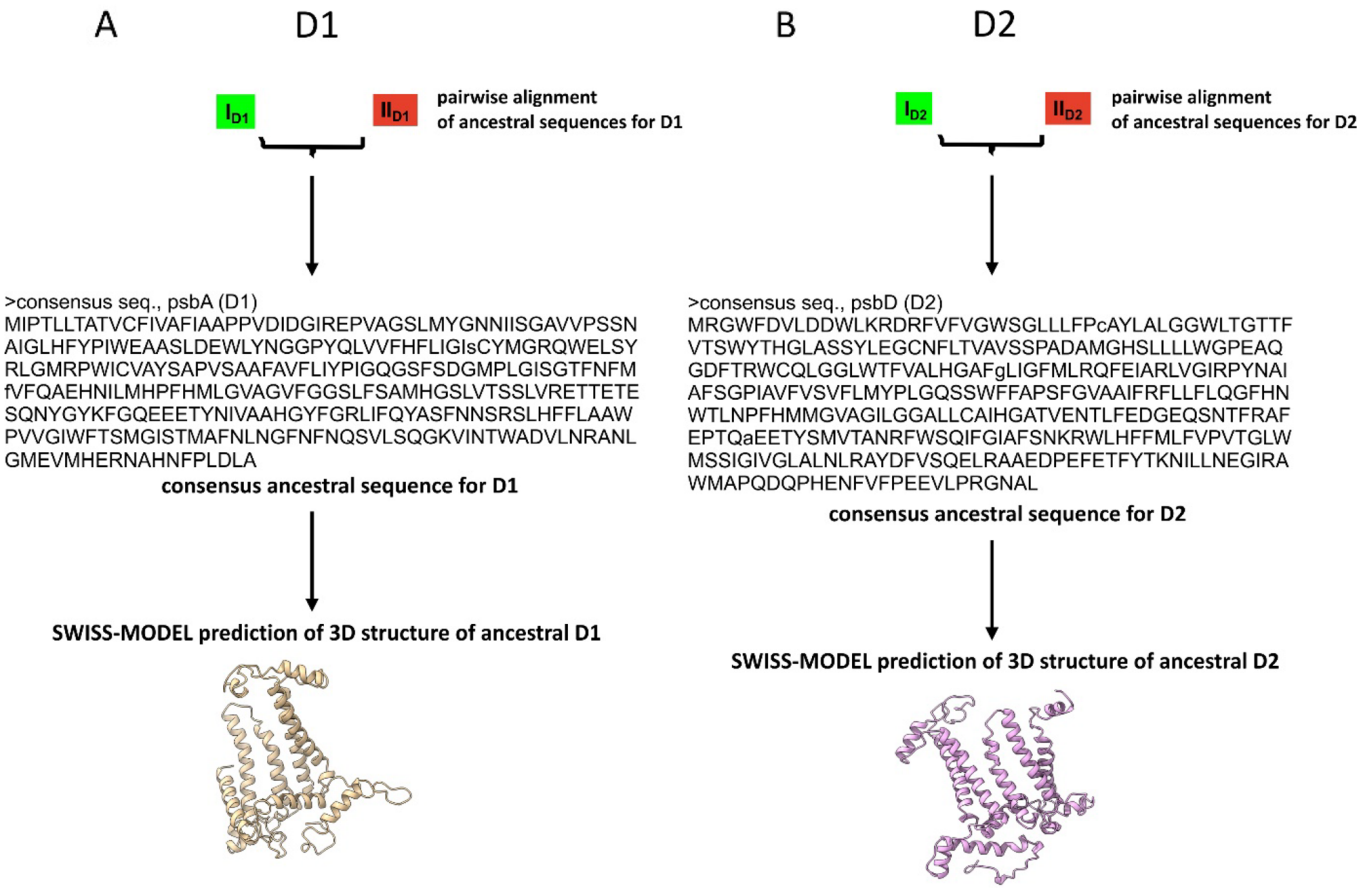
The scheme of ancestral sequence reconstruction for D1 and D2 proteins. (**A**) The consensus sequence for D1, obtained from the alignment of the ancestral sequences: I_D1_ and II_D1_ from the tree in Fig. 1A (Supplementary Information Notes S3), was used to reconstruct the spatial structure of the protein. (**B**) The consensus sequence for D2, obtained from the alignment of the ancestral sequences: I_D2_ and II_D2_ from the tree in Fig. 1B (Supplementary Information Notes S4), was used to reconstruct the spatial structure of the protein (see “Methods” for details).

The D1/D2 proteins are homologues formed by duplication of the original gene, and this duplication must have occurred long before the appearance of cyanobacteria^24,25^. The occurrence of early branched cyanobacterial D1 and D2 in clades dominated by cyanophage sequences also suggests an early and continuous gene exchange between cyanophages and their hosts (Figs. 1 and 5). Furthermore, it has already been confirmed that cyanophage genomes encode only D1 or both D1 and D2 proteins. According to our knowledge, no cyanophages have yet been found that have only *psbD* (D2) in their genome^3,10^. It is likely that *psbD* was later lost in some cyanophages. The results also show that purifying selection CDSs of *psbA* and *psbD*, early branching in the trees for D1/D2 proteins, and separate clades with dominance of cyanophage proteins indicate that the *psbA* and *psbD* genes for D1 and D2, respectively, must have evolved very early in both cyanophages and cyanobacteria. It cannot be excluded that the genes *psbA* and *psbD* had a common ancestor prior appearance of cyanobacteria-like organisms. Moreover, the reconstructed spatial structures for the predicted hypothetical ancestral D1 and D2 proteins suggest that they may have been functionally similar enough to the modern forms. The ability to bind the Mn_4_O_5_Ca cluster may even have been present in the case of the ancient form of the D1 protein. The common ancestor of the *psbA*/*psbD* genes and the corresponding D1 and D2 protein products proposed here is made plausible by previous data that the D1/D2 heterodimer may have preceded the homodimeric form D0^25^. The predicted structure of the hypothetical D0 protein suggests that it was more similar to the existing D1 than to the D2 sequences, and contained almost the same amino acids as D1, which are responsible for binding the Mn_4_O_5_Ca cluster. The only predicted mutation was at position 170, where D replaces E (D170E)^25^. Another important point related to the evolution of OP is also the structural similarity of the Ca-binding site between the homodimeric type I reaction centre (RCI) of green sulphur bacteria and heliobacteria and the Mn-binding site with the D/E residues in PSII^35–37^. Taken together, all these common structural features and the data based on molecular phylogeny provide further evidence for an early evolution of PSII and OEC.

As far as the origin of viruses is concerned, in connection with the phylogeny of *psbA* and *psbD* in cyanobacteria and cyanophages, one can ask whether it can not only be a rough model for the evolution of cyanobacteria and cyanophages, but whether it can also give an indication of how the evolution of viruses has proceeded in general. In the case of cyanophages and D1/D2 proteins, the results obtained indicate that the primordiality hypothesis of viruses in relation to their cellular hosts can be considered^38–41^. Our phylogenetic data showed that both the cyanophage and cyanobacterial D1/D2 proteins are deeply related, suggesting that the cyanophage and cyanobacterial ancestral sequences of *psbA* and *psbD* arose at least at the same time. This also suggests close co-evolution of *psbA* and *psbD* between hosts and their cyanophages. Therefore, the data seem to fit, at least in part, the 'virus early' hypothesis^38,39,41,42^. However, a certain problem with this virus/cyanophage origin scenario is the question of the origin of the proteins that build their capsids. Viral capsid proteins have no obvious homologues among the proteins of modern cells^43,44^. An analysis of cyanophage capsid proteins is beyond the scope of this study. Nevertheless, following new comparative analyses of capsid proteins, it has been hypothesised that protoviruses, the first replicons or mobile genetic elements, originated with the first cellular forms, i.e. the last universal common ancestor (LUCA), but that the capsid proteins were recruited from later primordial cellular forms^39,44^. All analysed here cyanophages belong to the realm *Duplodnaviria* that possess double-stranded DNA (dsDNA) (data form https://www.ncbi.nlm.nih.gov, October 2022). Comparative analyses of viruses and their hosts suggest that *Duplodnaviria*-like dsDNA viruses may have evolved from a population of replicators in the pre-LUCA era, which later gave rise to dsDNA viruses and cellular forms^39^.

At this point, a hypothetical scenario about the origin of viruses and cyanophages in particular is linked to the advent of OP. Cyanophages must have co-evolved with their hosts, as evidenced by the relatively abundant presence of *psbA* and *psbD* within them. Purifying selection for CDSs of *psbA*/*psbD* has already been described for the major groups of oxyphototrophs^26^. This fact, together with the conserved 3D structure of D1/D2 proteins predicted here, suggests that the ability to bind Mn_4_O_5_Ca clusters and to oxidise water may have arisen very early.

## Conclusions

In summary, the D1/D2 proteins and the genomes of the cyanophages that code for them arose in very early stages of the evolution of cyanobacteria and must have been in close relationship with the cyanophages (co-evolution). The in silico reconstructed 3D structures of the D1 and D2 proteins and the purifying selection estimated for the CDSs of *psbA* and *psbD* in the studied groups of oxyphototrophs and cyanophages suggest that D1/D2 are ancient proteins that may have formed the primordial PSII that could oxidise water at very early stages of the evolution of life on Earth.

## Methods

### Phylogenetic analysis of D1/D2 proteins

The molecular phylogenetic analysis was based on D1 and D2 amino acid sequences from selected group of organisms: cyanobacteria, algae, and plants. The latter are defined here as vascular and land plants. Moreover, D1/D2 proteins from cyanophages were also analysed. All sequences were derived from GenBank (www.ncbi.nlm.nih.gov) and UniProt (uniprot.org) databases (for details see Supplementary Information Notes S1 and Notes S2). The sequences were aligned with the default parameters of ClustalW implemented in MEGA6^45^. The evolutionary history was inferred by using the Maximum Likelihood method based on the Le and Gascuel^46^ model. A discrete Gamma distribution was used to model evolutionary rate differences among sites. The bootstrap consensus tree was inferred from 1000 replicates^47^. The tree is drawn to scale, with branch lengths measured in the number of substitutions per site. This analysis involved 48 and 39 proteins for D1 and D2, respectively.

### Ancestral sequence reconstruction and prediction of spatial structure

Based on the phylogenetic trees for D1 and D2 proteins, the deepest nodes were selected, designated I_D1_ and II_D1_ for D1 proteins and I_D2_ and II_D2_ for D2 proteins (Fig. 1). The ancestral amino acid sequences reconstructed for these nodes were pairwise aligned by the programme Cons (EMBOSS explorer, https://www.bioinformatics.nl/cgi-bin/emboss/cons) and the consensus sequences were determined (Fig. 5, Supplementary Information Notes S3 and Notes S4). A 3D structure for D1 and D2 was then predicted for the consensus sequences based on the SWISS-MODELL workspace on March 2022^48^. From the list of known PSII protein structures suggested as templates by the SWISS-MODEL algorithm, the protein structures of D1 (6w1p.1.U) and D2 (6dhe.1.D) for *T. elongatus* were used. The accession numbers, i.e.: 6w1p and 6dhe correspond to the 3D protein structures of PSII deposited in the Protein Data Bank (PDB) (https://www.rcsb.org). The selection was based on the fact that the 3D structures of the D1 and D2 proteins were determined by X-ray diffraction with a resolution of 2.26 and 2.05 Å, respectively^33,49^. The templates and the obtained models were pairwise alignment by EMBOSS needle software (Supplementary Information Notes S5 and Notes S6). The 3D protein structures were visualised and analysed with the software UCSF ChimeraX^31^.

### Coding sequences (CDSs) for *psbA*/*psbD* and determination of the values of non-synonymous (d_N_) and synonymous (d_S_) substitutions

The molecular phylogenetic analysis was based on CDSs for *psbA* and *psbD* taken from selected group of organisms: cyanobacteria, algae, and plants. Moreover, CDSs for these genes from cyanophages were also analysed. All sequences were derived from GenBank (www.ncbi.nlm.nih.gov), UniProt (www.uniprot.org), Kyoto Encyclopedia of Genes and Genomes (KEGG, www.genome.jp/kegg/), and European Nucleotide Archive (ENA, www.ebi.ac.uk/ena/browser/home) databases for details see Supplementary Information Notes S7 and Notes S8). The number of non-synonymous (d_N_) and synonymous (d_S_) nucleotide substitutions was determined in the analysed CDSs. The ratio of d_N_ to d_S_ (d_N_/d_S_) was calculated to estimate the type of selection. According to the d_N_/d_S_ method, d_N_/d_S_ = 1 means neutral evolution, d_N_/d_S_ < 1 indicates purifying (negative) selection, and d_N_/d_S_ > 1 means positive selection^29^. The sequences were aligned using the default parameters of ClustalW implemented in MEGA6^45^. The d_N_ and d_S_ values were calculated using the software DnaSP v. 6.12.03^50^, then d_N_/d_S_ values were determined.

## Supplementary Information

Supplementary Information.

## Data Availability

The datasets generated during and/or analysed during the current study are available from the corresponding author on reasonable request.
