# Supplementary material for: Cyanophages as an important factor in the early evolution of oxygenic photosynthesis
Source: Sci Rep. 2022 Nov 29;12:20581. doi: 10.1038/s41598-022-24795-1 (PMC9709159; doi:10.1038/s41598-022-24795-1)
Supplement: Supplementary file 1 — Supplementary Information. [file 41598_2022_24795_MOESM1_ESM.docx]

**Supplementary Information**

**Cyanophages as an important factor in the early evolution of oxygenic photosynthesis: a hypothesis worth considering**

**Ireneusz Ślesak, Halina Ślesak**

**Notes S1.** Amino acid sequences of D1 proteins

**Notes S2.** Amino acid sequences of D2 proteins

**Notes S3.** Ancestral sequences I_D1_ and II_D1_ predicted on the basis of the reconstructed phylogenetic tree for the D1 protein (Fig. 1A in the main text)

**Notes S4.** Ancestral sequences I_D2_ and II_D2_ predicted on the basis of the reconstructed phylogenetic tree for the D2 protein (Fig. 1B in the main text)

**Notes S5.** Alignment of the template sequence for the D1 protein (PDB, 6v1p.1.U) and the predicted model for D1

**Notes S6.** Alignment of the template sequence for the D2 protein (PDB, 6dhe.1.D) and the predicted model for D2

**Notes S7.** Coding sequences (CDSs) of *psbA* (D1)

**Notes S8.** Coding sequences (CDSs) of *psbD* (D2)

**Notes S1.** Amino acid sequences of D1 proteins

>YP_009188294.1 PsbA [Cyanophage P-TIM40]

MTTLSRRNSGELLQGWEQFCQWVTNTNNRIYVGWFGVLMIPCLLTAAACFIVAFIAAPPVDIDGIREPVAGSLLYGNNIISGAVVPSSNAIGLHFYPIWEAATVDEWLYNGGPYQLVVFHFLIGISAYMGRQWELSYRLGMRPWICVAYSAPVSAAMAVFLVYPFGQGSFSDGMPLGISGTFNFMFVFQAEHNILMHPFHMLGVAGVFGGSLFSAMHGSLVTSSLVRETTENESQNYGYKFGQEEETYNIVAAHGYFGRLIFQYASFNNSRSLHFFLAAFPVVCIWFTAMGVCTMAFNLNGFNFNQSVLDNSGKVIPTWGDILNRANLGMEVMHERNAHNFPLDLAAAETTQVALTAPSIG

>YP_214204.1 core photosystem II reaction center protein [Prochlorococcus virus PSSP7]

MAAISVTREGTTNWQKFCEWVTSTENRLYVGWFGVLMIPCLLAATTCFILAFIAAPPVDIDGIREPVSGSLMYGNNIISGAVVPSSNAIGLHFYPIWEAGTLDEWLYNGGPYQLVVFHFLIGVAAYAGRQWELSYRLGMRPWIFVAYTAPLSAALAVFLVYPFGQGSFSDGMPLGISGTFNFMFVFQAEHNILMHPFHMLGVAGVFGGSLFSAMHGSLVTSSIIKETTEDVSQNYGYKFGQDEETYNIVAAHGYFGRLIFQYASFNNSRSLHFFLATFPVVGIWLTSMGICTMAFNLNGFNFNQSVVDVNGKIIPTWADVLNRANLGFEVMHERNAHNFPLDLASAESTNIALTAPEIG

>YP_009807527.1 photosystem II D1 protein [Synechococcus T7-like phage S-TIP37]

MTAISYGQRSSSTWDDFCQWVTSTNNRLYVGWFGILMIPCLLAATLCFITAFVAAPPVDIDGIREPVAGSLMYGNNIISGAVVPSSNAIGLHFYPIWEANTLDEWLYNGGPYQLVVFHFLIGVFSYMGREWELSYRLGMRPWICVAYSAPVAAASAVFLVYPFGQGSFSDGMPLGISGTFNYMLVFQAEHNILMHPFHMLGVAGVFGGALFSAMHGSLVTSSLVRETTETESQNYGYKFGQEEETYNIVAAHGYFGRLIFQYASFNNSRSLHFFLAAWPVVGIWFAALGVSTMAFNLNGFNFNQSIQAQGHVVNTWADILNRANLGFEVMHERNAHNFPLDLAMASTTEVALQAPAIG

>YP_214516.1 photosystem II D1 protein [Prochlorococcus phage P-SSM2]

MTTLQKREQGLLSGWSEFCDWVTSTNNRIYVGWFGVLMIPCLLAATTCFIVAFIAAPPVDIDGIREPVAGSFMYGNNIISGAVVPSSNAIGLHFYPIWEAATLDEWLYNGGPYQLVIFHFLIGISAYMGRQWELSYRLGMRPWICVAYSAPVSAAFAVFLVYPFGQGSFSDGMPLGISGTFNFMFVFQAEHNILMHPFHMAGVAGMFGGALFSAMHGSLVTSSLIRETTGLDSQNYGYKFGQEEETYNIVAAHGYFGRLIFQYASFNNSRSLHFFLASWPVICVWLTSMGICTMAFNLNGFNFNQSVVDASGKVVPTWGDVLNRANLGMEVMHERNAHNFPLDLASANETEVALVAPSIG

>YP_009806476.1 photosystem II D1 protein [Cyanophage S-TIM4]

MTTLSRQGRQGGLLQGWPEFCEWVTSTNNRLYVGWFGVLMIPCLLTAAACFIVAFIAAPPVDIDGIREPVAGALMYGNNIISGAVVPSSNAIGLHFYPIWEAATIDEWLYNGGPYQLVIFHFLIGISAYMGRQWELSYRLGMRPWICVAYSAPVSAAFAVFLVYPFGQGSFSDGMPLGISGTFNFMFVFQAEHNILMHPFHMAGVAGMFGGSLFSAMHGSLVTSSLIRETTEEESQNYGYKFGQEEETYNIVAAHGYFGRLIFQYASFNNSRSLHFFLAVFPVVCVWLTSMGICTMAFNLNGFNFNQSVVDVNGKIIPTWGDVLNRANLGMEVMHERNAHNFPLDLASAETTEVALTAPTIG

>YP_004325052.1 photosystem II D1 protein [Prochlorococcus phage P-SSM7]

MTTLQKRETGLLAGWPQFTDWVTSTNNRIYVGWFGVLMIPCLLAAATCFIVAFIAAPPVDIDGIREPVAGSFMYGNNIISGAVVPSSNAIGLHFYPIWEAATLDEWLYNGGPYQLVIFHFLIGISAYMGRQWELSYRLGMRPWICVAYSAPVSAAFAVFLVYPFGQGSFSDGMPLGISGTFNFMFVFQAEHNILMHPFHMAGVAGMFGGALFSAMHGSLVTSSLIRETTDNESQNYGYKFGQEEETYNIVAAHGYFGRLIFQYASFNNSRSLHFFLATFPVVCIWLTSMGICTMAFNLNGFNFNQSIVDGSGKVVPTWADVLNRANLGMEVMHERNAHNFPLDLATAETSEVALIAPSVG

>YP_004324586.1 photosystem II D1 protein [Prochlorococcus phage Syn1]

MASTLSRQQSTSSWEQFCEWVTSTNNRLYVGWFGVLMIPTLLAATICFIVAFVAAPPVDIDGIREPVAGSLMYGNNIISGAVVPSSNAIGLHFYPIWEAASLDEWLYNGGPFQLVVFHFLIGIYAYMGREWELSYRLGMRPWICVAYSAPVAAASAVFLVYPFGQGSFSDAMPLGISGTFNYMLVFQAEHNILMHPFHMLGVAGVFGGSLFSAMHGSLVTSSLVRETTEQESQNYGYKFGQEEETYNIVAAHGYFGRLIFQYASFNNSRSLHFFLAAWPVVGIWFTALGVSTMAFNLNGFNFNQSILDGQGRVLNTWADVLNRAGLGMEVMHERNAHNFPLDLAAAESTPVALVAPSVG

>YP_004324037.1 photosystem II D1 protein [Synechococcus phage Syn19]

MASSTLSRSRVSNWESFCDWVTSTNNRLYVGWFGVLMIPTLLAATICFIIAFVGAPPVDIDGIREPVAGSLMYGNNIISGAVVPSSNAIGLHFYPIWEAATLDEWLYNGGPFQLVVFHFLIGIYAYMGREWELSYRLGMRPWICVAYSAPVAAASAVFLVYPFGQGSFSDAMPLGISGTFNYMLVFQAEHNILMHPFHMLGVAGVFGGSLFSAMHGSLVTSSLVRETTETESQNYGYKFGQEEETYNIVAAHGYFGRLIFQYASFNNSRSLHFFLAAWPVVGIWFTALGVSTMAFNLNGFNFNQSILDSSGKVLPTWADVLNRAGLGMEVMHERNAHNFPLDLAAAESTPVALTAPAIGA

>YP_004323819.1 photosystem II D1 protein [Prochlorococcus phage Syn33]

MATTLSRQQSTSPWNDFCEWVTSTNNRLYVGWFGVLMIPTLLAATICFIVAFVAAPPVDIDGIREPVAGSLMYGNNIISGAVVPSSNAIGLHFYPIWEAASLDEWLYNGGPFQLVVFHFLIGIYAYMGREWELSYRLGMRPWICVAYSAPVAAASAVFLVYPFGQGSFSDAMPLGISGTFNYMLVFQAEHNILMHPFHMLGVAGVFGGSLFSAMHGSLVTSSLVRETTETESQNYGYKFGQEEETYNIVAAHGYFGRLIFQYASFNNSRSLHFFLAAWPVVGIWFTALGVSTMAFNLNGFNFNQSILDGQGRVLNTWADVLNRAGLGMEVMHERNAHNFPLDLAAAESTPVALTAPTVG

>YP_004323112.1 photosystem II D1 protein [Synechococcus phage S-SM1]

MATTLSRQSGVSSWESFCEWVTSTNNRLYVGWFGVLMIPTLLAATICFIVAFVAAPPVDIDGIREPVAGSLMYGNNIISGAVVPSSNAIGLHFYPIWEAASLDEWLYNGGPFQLVVFHFLIGIYAYMGREWELSYRLGMRPWICVAYSAPVAAASAVFLVYPFGQGSFSDAMPLGISGTFNYMLVFQAEHNILMHPFHMLGVAGVFGGSLFSAMHGSLVTSSLVRETTETESQNYGYKFGQEEETYNIVAAHGYFGRLIFQYASFNNSRSLHFFLAAWPVVGIWFTALGVSTMAFNLNGFNFNQSIIDGQGRVLNTWADVLNRAGLGMEVMHERNAHNFPLDLAAAESTPVALTAPAIG

>YP_004322876.1 photosystem II D1 protein [Synechococcus phage S-ShM2]

MATTLSRQQTSPWNDFCEWVTSTNNRLYVGWFGVLMIPTLLAATICFIVAFVAAPPVDIDGIREPVAGSLMYGNNIISGAVVPSSNAIGLHFYPIWEAASLDEWLYNGGPFQLVVFHFLIGIYAYMGREWELSYRLGMRPWICVAYSAPVAAASAVFLVYPFGQGSFSDAMPLGISGTFNYMLVFQAEHNILMHPFHMLGVAGVFGGSLFSAMHGSLVTSSLVRETTETESQNYGYKFGQEEETYNIVAAHGYFGRLIFQYASFNNSRSLHFFLAAWPVVGIWFTALGVSTMAFNLNGFNFNQSILDGQGRVLNTWADVLNRAGLGMEVMHERNAHNFPLDLAAAESTPVALIAPSVG

>YP_005087457.1 photosystem II reaction center protein PsbA/D1 [Cyanophage NATL1A-7]

MTTATLSPLSNWDKFCDWVTSTNNRLYVGWFGVLMIPALLTATTAFILAFVAAPPVDIDGIREPVAGSLLYGNNIISGAVVPSSNAIGLHFYPIWEAANLDEWLYNGGPYQLVVFHFLIGIAAYLGRQWELSYRLGMRPWICVAYSAPVAAAYSVFLVYPFGQGSFSDGMPLGISGTFNFMFVFQAEHNILMHPFHMLGVAGVFGGALFAAMHGSLVTSSLIRETTGLTSQNYGYKFGQEEETYNIVAAHGYFGRLIFQYASFNNSRSLHFFLATWPVVCIWLTSMGISTMAFNLNGFNFNQSVVDSSGRIVPTWADVLNRANLGMEVMHERNAHNFPLDLAAAESSEVALTAPSIG

>YP_007006003.1 D1 protein [Cyanophage S-TIM5]

MSASTLSRQQSQSTWENFCEWVTSTNNRLYVGWFGVLMIPTLLAATICFITAFVAAPPVDIDGIREPVAGSLMYGNNIISGAVVPSSNAIGLHFYPIWEAASLDEWLYNGGPYQLVVFHFLLGVFAYMGREWELSYRLGMRPWICVAYSAPVAAASAVFLVYPFGQGSFSDGMPLGISGTFNYMLVFQAEHNILMHPFHMLGVAGVFGGSLFSAMHGSLVTSSLVRETTETESQNYGYKFGQEEETYNIVAAHGYFGRLIFQYASFNNSRSLHFFLAAWPVVGIWFTALGVSTMAFNLNGFNFNQSIIDGQGRVLNTWADVLNRAGLGMEVMHERNAHNFPLDLAAAESTPVALTAPSIG

>YP_009783352.1 photosystem II protein D1 [Cyanophage S-RIM44]

MASTLSRQQSTSSWEQFCEWVTSTNNRLYVGWFGVLMIPTLLAATICFIVAFVAAPPVDIDGIREPVAGSLMYGNNIISGAVVPSSNAIGLHFYPIWEAASLDEWLYNGGPFQLVVFHFLIGIYAYMGREWELSYRLGMRPWICVAYSAPVAAASAVFLVYPFGQGSFSDAMPLGISGTFNYMLVFQAEHNILMHPFHMLGVAGVFGGSLFSAMHGSLVTSSLVRETTETESQNYGYKFGQEEETYNIVAAHGYFGRLIFQYASFNNSRSLHFFLAAWPVVGIWFTALGVSTMAFNLNGFNFNQSILDGQGRVLNTWADVLNRAGLGMEVMHERNAHNFPLDLAAAESTPVALVAPSVG

>YP_009783122.1 photosystem II protein D1 [Synechococcus phage S-RIM8]

MTATLSQQRSTNTWEQFCNWVTSTDNRLYVGWFGVLMIPCLLAATICFIIAFVGAPPVDIDGIREPVAGSLMYGNNIISGAVIPSSNAIGLHFYPIWEAASLDEWLYNGGPFQLVVFHFLIGIYAYMGREWELSYRLGMRPWICVAYSAPVAAASAVFLVYPFGQGSFSDAMPLGISGTFNYMLVFQAEHNILMHPFHMLGVAGVFGGSLFSAMHGSLVTSSLVRETTENESQNYGYKFGQEEETYNIVAAHGYFGRLIFQYASFNNSRSLHFFLAAWPVVGIWFTALGVSTMAFNLNGFNFNQSIVDSQGKVINTWADVLNRAGLGMEVMHERNAHNFPLDLAAAESTPVALQAPAIG

>YP_009324374.1 photosystem II protein D1 [Synechococcus phage S-WAM2]

MIPTLLAAAICFIVAFVAAPPVDIDGIREPVAGSLMYGNNIISGAVVPSSNAIGLHFYPIWEAASLDEWLYNGGPFQLVIFHFLIGIYAYMGREWELSYRLGMRPWICVAYSAPVAAASAVFLVYPFGQGSFSDAMPLGISGTFNYMLVFQAEHNILMHPFHMLGVAGVFGGSLFSAMHGSLVTSSLVRETTENESQNYGYKFGQEEETYNIVAAHGYFGRLIFQYASFNNSRSLHFFLAAWPVVGIWFTALGVSTMAFNLNGFNFNQSIMDSQGKVLNTWADVLNRAGLGMEVMHERNAHNFPLDLAAAESTPVALTAPAVG

>YP_009322638.1 photosystem II protein D1 [Synechococcus phage S-CAM9]

MANSTLSQGYNQQSTWEQFCEWVTSTNNRLYVGWFGVLMIPTLLAATVCFITAFVAAPPVDIDGIREPVAGSLMYGNNIISGAVVPSSNAIGLHFYPIWEAASLDEWLYNGGPYQLVVFHFLIGIFCYMGREWELSYRLGMRPWICVAYSAPVAAASAVFLVYPFGQGSFSDGMPLGISGTFNFMLVFQAEHNILMHPFHMLGVAGVFGGSLFSAMHGSLVTSSLVRETTENESQNYGYKFGQEEETYNIVAAHGYFGRLIFQYASFNNSRSLHFFLAAWPVVGIWFTALGVSTMAFNLNGFNFNQSIMDGQGKVLNTWADVLNRANLGMEVMHERNAHNFPLDLAAAESTPVALTAPTVG

>YP_009321483.1 photosystem II protein D1 [Synechococcus phage S-CAM3]

MTATLSRQQSGTWESFCEWVTSTNNRLYVGWFGVLMIPTLLAATICFIVAFVAAPPVDIDGIREPVAGSLMYGNNIISGAVVPSSNAIGLHFYPIWEAASLDEWLYNGGPFQLVVFHFLIGIYAYMGREWELSYRLGMRPWICIAYSAPVAAASAVFLVYPFGQGSFSDAMPLGISGTFNYMLVFQAEHNILMHPFHMLGVAGVFGGSLFSAMHGSLVTSSLVRETTETESQNYGYKFGQEEETYNIVAAHGYFGRLIFQYASFNNSRSLHFFLAAWPVVGIWFTALGVSTMAFNLNGFNFNQSILDGQGRVLNTWADVLNRAGLGMEVMHERNAHNFPLDLAAAESTPVALTAPAIG

>YP_009302297.1 photosystem II protein D1 [Cyanophage S-RIM50]

MTATLSQQRSTNTWEQFCNWVTSTDNRLYVGWFGVLMIPCLLAATTCFIIAFIGAPPVDIDGIREPVAGSLMYGNNIISGAVIPSSNAIGLHFYPIWEAASLDEWLYNGGPFQLVVFHFLIGIYAYMGREWELSYRLGMRPWICVAYSAPVAAASAVFLVYPFGQGSFSDAMPLGISGTFNYMLVFQAEHNILMHPFHMLGVAGVFGGSLFSAMHGSLVTSSLVRETTENESQNYGYKFGQEEETYNIVAAHGYFGRLIFQYASFNNSRSLHFFLAAWPVVGIWFTALGVSTMAFNLNGFNFNQSIIDGQGRVLNTWADVLNRAGLGMEVMHERNAHNFPLDLAAAENTPVALKAPAVG

>sp|P83755|PSBA_ARATH Photosystem II protein D1 OS=Arabidopsis thaliana OX=3702 GN=psbA PE=1 SV=2

MTAILERRESESLWGRFCNWITSTENRLYIGWFGVLMIPTLLTATSVFIIAFIAAPPVDIDGIREPVSGSLLYGNNIISGAIIPTSAAIGLHFYPIWEAASVDEWLYNGGPYELIVLHFLLGVACYMGREWELSFRLGMRPWIAVAYSAPVAAATAVFLIYPIGQGSFSDGMPLGISGTFNFMIVFQAEHNILMHPFHMLGVAGVFGGSLFSAMHGSLVTSSLIRETTENESANEGYRFGQEEETYNIVAAHGYFGRLIFQYASFNNSRSLHFFLAAWPVVGIWFTALGISTMAFNLNGFNFNQSVVDSQGRVINTWADIINRANLGMEVMHERNAHNFPLDLAAVEAPSTNG

>sp|P69560|PSBA_SPIOL Photosystem II protein D1 OS=Spinacia oleracea OX=3562 GN=psbA PE=1 SV=2

MTAILERRESESLWGRFCNWITSTENRLYIGWFGVLMIPTLLTATSVFIIAFIAAPPVDIDGIREPVSGSLLYGNNIISGAIIPTSAAIGLHFYPIWEAASVDEWLYNGGPYELIVLHFLLGVACYMGREWELSFRLGMRPWIAVAYSAPVAAATAVFLIYPIGQGSFSDGMPLGISGTFNFMIVFQAEHNILMHPFHMLGVAGVFGGSLFSAMHGSLVTSSLIRETTENESANEGYRFGQEEETYNIVAAHGYFGRLIFQYASFNNSRSLHFFLAAWPVVGIWFTALGISTMAFNLNGFNFNQSVVDSQGRVINTWADIINRANLGMEVMHERNAHNFPLDLAAIEAPSTNG

>sp|P0A444|PSBA1_THEEB Photosystem II protein D1 1 OS=Thermosynechococcus elongatus (strain BP-1) OX=197221 GN=psbA1 PE=1 SV=1

MTTTLQRRESANLWERFCNWVTSTDNRLYVGWFGVIMIPTLLAATICFVIAFIAAPPVDIDGIREPVSGSLLYGNNIITGAVVPSSNAIGLHFYPIWEAASLDEWLYNGGPYQLIIFHFLLGASCYMGRQWELSYRLGMRPWICVAYSAPLASAFAVFLIYPIGQGSFSDGMPLGISGTFNFMIVFQAEHNILMHPFHQLGVAGVFGGALFCAMHGSLVTSSLIRETTETESANYGYKFGQEEETYNIVAAHGYFGRLIFQYASFNNSRSLHFFLAAWPVVGVWFTALGISTMAFNLNGFNFNHSVIDAKGNVINTWADIINRANLGMEVMHERNAHNFPLDLASAESAPVAMIAPSING

>sp|P07826|PSBA1_SYNY3 Photosystem II protein D1 1 OS=Synechocystis sp. (strain PCC 6803 / Kazusa) OX=1111708 GN=psbA1 PE=1 SV=3

MTTTQLGLQEQSLWSRFCCWITSTSNRLYIGWFGVLMIPTLLTATTCFIIAFIAAPPVDIDGIREPIAGSLLYGNNIITAAVVPSSNAIGLHFYPIWEAHSLDEWLYNGGPYQLIVFHFLIGIFCYLGRQWELSYRLGMRPWICVAYSAPVAAATATLLIYSIGQGSFSDGLPLGISGTFNFMLVLQAEHNVLMHPFHMLGVAGVFGGALFAAMHGSLVTSSLIRETTEVESQNQGYKFGQEEETYNIVAAHGYFGRLIFQYASFNNSRALHFFLGAWPVVGIWFAALAVCCFAFNLNGFNFNQSILDAQGRPVSTWADVINRANIGFEVMHERNVHNFPLDLASGDAQMVALNAPAIEG

>sp|P04996|PSBA1_SYNE7 Photosystem II protein D1 1 OS=Synechococcus elongatus (strain PCC 7942) OX=1140 GN=psbA1 PE=1 SV=1

MTSILREQRRDNVWDRFCEWVTSTDNRIYVGWFGVLMIPTLLTATICFIVAFIAAPPVDIDGIREPVAGSLMYGNNIISGAVVPSSNAIGLHFYPIWEAASLDEWLYNGGPYQLVVFHFLLGISCYMGRQWELSYRLGMRPWICVAYSAPLSAAFAVFLIYPIGQGSFSDGMPLGISGTFNFMFVFQAEHNILMHPFHMLGVAGVFGGSLFSAMHGSLVTSSLVRETTETESQNYGYKFGQEEETYNIVAAHGYFGRLIFQYASFNNSRSLHFFLGAWPVVGIWFTSMGISTMAFNLNGFNFNQSVLDSQGKVINTWADVLNRANLGMEVMHERNAHNFPLDLAAGEATPVALTAPSIHG

>sp|P06631|PSBA_EUGGR Photosystem II protein D1 OS=Euglena gracilis OX=3039 GN=psbA PE=1 SV=2

MISPVLKKYARPSLWYRFCAWVASKKNRLYVGWFGVLMIPTLLTAATVFIIAFIAAPPVDIDGIREPVSGSLFYGNNIITGAVVPTSNAIGLHFYPIWEATSLDEWLYNGGPYQLIVCHFFIGICSYMGREWELSFRLGMRPWIAVAYSAPVAAASAVFIVYPLGQGSFSDGMPLGISGTFNFMIVFQAEHNILMHPFHMLGVAGVFGGSLFSAMHGSLVTSSLLRETTENESINVGYKFGQEEETYNIIAAHAYFGRLIFQYASFNNSRSLHFFLAVWPVVGIWFTALGVSTMAFNLNGFNFNQSVIDSQGRVINTWADIINRANLGMEVMHERNAHNFPLDLA

>sp|P51765|PSBA_THEVL Photosystem II protein D1 OS=Thermosynechococcus vulcanus OX=32053 GN=psbA PE=1 SV=1

MTTTLQRRESANLWERFCNWVTSTDNRLYVGWFGVIMIPTLLAATICFVIAFIAAPPVDIDGIREPVSGSLLYGNNIITGAVVPSSNAIGLHFYPIWEAASLDEWLYNGGPYQLIIFHFLLGASCYMGRQWELSYRLGMRPWICVAYSAPLASAFAVFLIYPIGQGSFSDGMPLGISGTFNFMIVFQAEHNILMHPFHQLGVAGVFGGALFCAMHGSLVTSSLIRETTETESANYGYKFGQEEETYNIVAAHGYFGRLIFQYASFNNSRSLHFFLAAWRVVGVWFAALGISTMAFNLNGFNFNHSVIDAKGNVINTWADIINRANLGMEVMHERNAHNFPLDLASAESAPVAMIAPSING

>sp|Q2VEJ6|PSBA_SOLTU Photosystem II protein D1 OS=Solanum tuberosum OX=4113 GN=psbA PE=3 SV=1

MTAILERRESESLWGRFCNWITSTENRLYIGWFGVLMIPTLLTATSVFIIAFIAAPPVDIDGIREPVSGSLLYGNNIISGAIIPTSAAIGLHFYPIWEAASVDEWLYNGGPYELIVLHFLLGVACYMGREWELSFRLGMRPWIAVAYSAPVAAATAVFLIYPIGQGSFSDGMPLGISGTFNFMIVFQAEHNILMHPFHMLGVAGVFGGSLFSAMHGSLVTSSLIRETTENESANEGYRFGQEEETYNIVAAHGYFGRLIFQYASFNNSRSLHFFLAAWPVVGIWFTALGISTMAFNLNGFNFNQSVVDSQGRVINTWADIINRANLGMEVMHERNAHNFPLDLAAIEAPSTNG

>sp|Q2MIC0|PSBA_SOLLC Photosystem II protein D1 OS=Solanum lycopersicum OX=4081 GN=psbA PE=3 SV=1

MTAILERRESESLWGRFCNWITSTENRLYIGWFGVLMIPTLLTATSVFIIAFIAAPPVDIDGIREPVSGSLLYGNNIISGAIIPTSAAIGLHFYPIWEAASVDEWLYNGGPYELIVLHFLLGVACYMGREWELSFRLGMRPWIAVAYSAPVAAATAVFLIYPIGQGSFSDGMPLGISGTFNFMIVFQAEHNILMHPFHMLGVAGVFGGSLFSAMHGSLVTSSLIRETTENESANEGYRFGQEEETYNIVAAHGYFGRLIFQYASFNNSRSLHFFLAAWPVVGIWFTALGISTMAFNLNGFNFNQSVVDSQGRVINTWADIINRANLGMEVMHERNAHNFPLDLAAIEAPSTNG

>sp|P06585|PSBA_PEA Photosystem II protein D1 OS=Pisum sativum OX=3888 GN=psbA PE=1 SV=3

MTAILERRDSENLWGRFCNWITSTENRLYIGWFGVLMIPTLLTATSVFIIAFIAAPPVDIDGIREPVSGSLLYGNNIISGAIIPTSAAIGLHFYPIWEAASVDEWLYNGGPYELIVLHFLLGVACYMGREWELSFRLGMRPWIAVAYSAPVAAATAVFLIYPIGQGSFSDGMPLGISGTFNFMIVFQAEHNILMHPFHMLGVAGVFGGSLFSAMHGSLVTSSLIRETTENESANEGYRFGQEEETYNIVAAHGYFGRLIFQYASFNNSRSLHFFLAAWPVVGIWFTALGISTMAFNLNGFNFNQSVVDSQGRVINTWADIINRANLGMEVMHERNAHNFPLDLAAVEAPSING

>sp|P69556|PSBA_TOBAC Photosystem II protein D1 OS=Nicotiana tabacum OX=4097 GN=psbA PE=3 SV=2

MTAILERRESESLWGRFCNWITSTENRLYIGWFGVLMIPTLLTATSVFIIAFIAAPPVDIDGIREPVSGSLLYGNNIISGAIIPTSAAIGLHFYPIWEAASVDEWLYNGGPYELIVLHFLLGVACYMGREWELSFRLGMRPWIAVAYSAPVAAATAVFLIYPIGQGSFSDGMPLGISGTFNFMIVFQAEHNILMHPFHMLGVAGVFGGSLFSAMHGSLVTSSLIRETTENESANEGYRFGQEEETYNIVAAHGYFGRLIFQYASFNNSRSLHFFLAAWPVVGIWFTALGISTMAFNLNGFNFNQSVVDSQGRVINTWADIINRANLGMEVMHERNAHNFPLDLAAIEAPSTNG

>sp|P12463|PSBA_WHEAT Photosystem II protein D1 OS=Triticum aestivum OX=4565 GN=psbA PE=3 SV=2

MTAILERRESTSLWGRFCNWITSTENRLYIGWFGVLMIPTLLTATSVFIIAFIAAPPVDIDGIREPVSGSLLYGNNIISGAIIPTSAAIGLHFYPIWEAASVDEWLYNGGPYELIVLHFLLGVACYMGREWELSFRLGMRPWIAVAYSAPVAAATAVFLIYPIGQGSFSDGMPLGISGTFNFMIVFQAEHNILMHPFHMLGVAGVFGGSLFSAMHGSLVTSSLIRETTENESANEGYKFGQEEETYNIVAAHGYFGRLIFQYASFNNSRSLHFFLAAWPVVGIWFTALGISTMAFNLNGFNFNQSVVDSQGRVINTWADIINRANLGMEVMHERNAHNFPLDLAAVEVPSING

>sp|P46242|PSBA1_NOSS1 Photosystem II protein D1 1 OS=Nostoc sp. (strain PCC 7120 / SAG 25.82 / UTEX 2576) OX=103690 GN=psbA1 PE=3 SV=1

MTTTLQQRSSANVWERFCTWITSTENRIYVGWFGVLMIPTLLAATVCFIIAFVAAPPVDIDGIREPVAGSLIYGNNIISGAVVPSSNAIGLHFYPIWEAASLDEWLYNGGPYQLVIFHFLIGCACYLGRQWELSYRLGMRPWICVAYSAPLASATAVFLIYPIGQGSFSDGMPLGISGTFNFMIVFQAEHNILMHPFHMLGVAGVFGGSLFSAMHGSLVTSSLVRETTEIESQNYGYKFGQEEETYNIVAAHGYFGRLIFQYASFNNSRQLHFFLAAWPVIGIWFTALGVSTMAFNLNGFNFNQSIIDSQGRVINTWADIINRANLGMEVMHERNAHNFPLDLAAGEVAPVALTAPAING

>sp|P07753|PSBA_CHLRE Photosystem II protein D1 OS=Chlamydomonas reinhardtii OX=3055 GN=psbA PE=1 SV=1

MTAILERRENSSLWARFCEWITSTENRLYIGWFGVIMIPCLLTATSVFIIAFIAAPPVDIDGIREPVSGSLLYGNNIITGAVIPTSNAIGLHFYPIWEAASLDEWLYNGGPYQLIVCHFLLGVYCYMGREWELSFRLGMRPWIAVAYSAPVAAASAVFLVYPIGQGSFSDGMPLGISGTFNFMIVFQAEHNILMHPFHMLGVAGVFGGSLFSAMHGSLVTSSLIRETTENESANEGYRFGQEEETYNIVAAHGYFGRLIFQYASFNNSRSLHFFLAAWPVIGIWFTALGLSTMAFNLNGFNFNQSVVDSQGRVLNTWADIINRANLGMEVMHERNAHNFPLDLASTNSSSNN

>tr|D0VMW3|D0VMW3_VOLCA Photosystem II protein D1 OS=Volvox carteri f. nagariensis OX=3068 GN=psbA PE=3 SV=1

MTAILERRENSSLWARFCEWITSTENRLYIGWFGVIMIPCLLTATSVFIIAFIAAPPVDIDGIREPVSGSLLYGNNIITGAVVPTSNAIGLHFYPIWEAASLDEWLYNGGPYQLIVCHFLLGVYCYMGREWELSFRLGMRPWIAVAYSAPVAAASAVFLVYPIGQGSFSDGMPLGISGTFNFMIVFQAEHNILMHPFHMLGVAGVFGGSLFSAMHGSLVTSSLIRETTENESANEGYRFGQEEETYNIVAAHGYFGRLIFQYASFNNSRSLHFFLAAWPVIGIWFTALGLSTMAFNLNGFNFNQSVVDSQGRVLNTWADIINRANLGMEVMHERNAHNFPLDLASNNNSSMN

>sp|P51759|PSBA_CROS5 Photosystem II protein D1 OS=Crocosphaera subtropica (strain ATCC 51142 / BH68) OX=43989 GN=psbA1 PE=3 SV=2

MTTTLQQRESVSLWEQFCQWVTSTNNRIYVGWFGTLMIPTLLTATTCFIIAFIAAPPVDIDGIREPVAGSLLYGNNIVSGAVVPSSNAIGLHFYPIWEAASLDEWLYNGGPYQLVIFHFLIGIFCYMGRQWELSYRLGMRPWICVAYSAPVSAATAVFLIYPIGQGSFSDGMPLGISGTFNFMFVFQAEHNILMHPFHMLGVAGVFGGSLFSAMHGSLVTSSLVRETTEIESQNYGYKFGQEEETYNIVAAHGYFGRLIFQYASFNNSRALHFFLGAWPVIGIWFTAMGVSTMAFNLNGFNFNQSILDSQGRVIGTWADVLNRAGIGMEVMHERNAHNFPLDLASAEPVSAPVING

>sp|Q7M7A9|PSBA1_GLOVI Photosystem II protein D1 1 OS=Gloeobacter violaceus (strain ATCC 29082 / PCC 7421) OX=251221 GN=psbA1 PE=3 SV=1

MTATLERRSSQGLWDRFADWVTSTNNRFYVGWFGVLMIPTLLSATICFVVAFVAAPPVDMDGIREPISGSLLYGNNIITGAVIPSSNAIGLHFYPIWEAASMDEWLYNGGPYQLVVFHFLIGVFCYLGREWELSYRLGLRPWICIAYSAPVAAAAAVFLIYPIGQGSFSDGMPLGISGTFNFMFVFQAEHNILNHPFHMLGVAGVFGGSLFSAMHGSLVTSSLIRETSMEESQNYGYKFGQEEETYNIIAAHGYFGRLIFQYASFNNSRSLHFFLAAWPVIGIWFTALGISVMAFNLNGFNFNSSIVDSQGRAIYTWADIVNRANLGMEVMHERNAHNFPLDLAGTESAPVAVGNADLNG

>tr|K9S5G7|K9S5G7_9CYAN Photosystem II protein D1 OS=Geitlerinema sp. PCC 7407 OX=1173025 GN=psbA PE=3 SV=1

MTTTLQRREGGSLWERFCSWVTSTDNRLYVGWFGVLMIPTLLTATVCFIIAFVAAPPVDIDGIREPVAGSLLYGNNIISGAVVPSSNAIGLHFYPIWEAASLDEWLYNGGPYQLVVCHFLIGISCYMGRQWELSYRLGMRPWICVAYSAPLSAAFAVFLIYPIGQGSFSDGMPLGISGTFNFMFVFQAEHNILMHPFHMLGVAGVFGGSLFSAMHGSLVTSSLVRETTENESQNAGYKFGQEEETYNIVAAHGYFGRLIFQYASFNNSRSLHFFLGAWPVVGIWFTSMGISTMAFNLNGFNFNQSVLDSQGRVVGTWADVLNRANLGMEVMHERNAHNFPLDLASGDEMPVALQAPAIHG

>sp|A4GYN9|PSBA_POPTR Photosystem II protein D1 OS=Populus trichocarpa OX=3694 GN=psbA PE=2 SV=1

MTAILERRESESLWGRFCNWITSTENRLYIGWFGVLMIPTLLTATSVFIIAFIAAPPVDIDGIREPVSGSLLYGNNIISGAIIPTSAAIGLHFYPIWEAASVDEWLYNGGPYELIVLHFLLGVACYMGREWELSFRLGMRPWIAVAYSAPVAAATAVFLIYPIGQGSFSDGMPLGISGTFNFMIVFQAEHNILMHPFHMLGVAGVFGGSLFSAMHGSLVTSSLIRETTENESANEGYRFGQEEETYNIVAAHGYFGRLIFQYASFNNSRSLHFFLAAWPVVGIWFTALGISTMAFNLNGFNFNQSVVDSQGRVINTWADIINRANLGMEVMHERNAHNFPLDLAAVEVPSTNG

>tr|U5QLI7|U5QLI7_9CYAN Photosystem II protein D1 OS=Gloeobacter kilaueensis JS1 OX=1183438 GN=psbA PE=3 SV=1

MTATLERRSTQGLWERFADWVTSTNNRFYVGWFGVLMIPTLLSATICYIVAFIAAPPVDMDGIREPISGSLLYGNNIITGAVIPSSNAIGLHFYPIWEAASMDEWLYNGGPYQLVVFHFLIGVFCYLGREWELSYRLGLRPWICIAYSAPVAAATAVFLIYPIGQGSFSDGMPLGISGTFNFMFVFQAEHNILNHPFHMLGVAGVFGGSLFSAMHGSLVTSSLIRETSYEESQNYGYKFGQEEETYNIIAAHGYFGRLIFQYASFNNSRSLHFFLAAWPVVGIWFTALGISVMAFNLNGFNFNSSIVDSQGRAIYTWADVVNRANLGMEVMHERNAHNFPLDLASSESVPVAVSTADLNG

>sp|P51764|PSBA_MICAE Photosystem II protein D1 OS=Microcystis aeruginosa OX=1126 GN=psbA PE=3 SV=1

MTTTLQQRESASLWEQFCQWITSTNNRLYVGWFGVIMIPTLLTATTCFIIAFIAAPPVDIDGIREPVAGSLLYGNNIISGAVVPSSNAIGLHFYPIWEAASLDEWLYNGGPYQLVIFHFLLGVFCYLGRQWELSFRLGMRPWICVAYSAPVSAATAVFLIYPIGQGSFSDGMPLGISGTFNFMFVFQAEHNILMHPFHMLGVAGVFGGSLFSAMHGSLVTSSLVRETTEIESQNYGYKFGQEEETYNIVAAHGYFGRLIFQYASFNNSRSLHFFLGAWPVIGIWFTAMGVSTMAFNLNGFNFNQSILDSQGRVIGTWVDVLNRAGIGMEVMHERNAHNFPLDLASGEQAPVALTAPAING

>tr|A0A0M4SU62|A0A0M4SU62_9NOSO Photosystem II protein D1 OS=Nostoc piscinale CENA21 OX=224013 GN=psbA PE=3 SV=1

MTATLQQRRSANVWEQFCNWITSTNNRLYIGWFGVLMIPTLLAATTCFVIAFIAAPPVDIDGIREPVAGSLLYGNNIISGAVVPSSNAIGLHFYPIWEAASLDEWLYNGGPYQLVIFHFLIGVFCYLGREWELSYRLGMRPWICLAFSAPVAAATAVFLIYPIGQGSFSDGMPLGISGTFNFMIVFQAEHNILMHPFHMLGVAGVFGGSLFSAMHGSLVTSSLVRETTENESQNYGYKFGQEEETYNIVAAHGYFGRLIFQYASFNNSRSLHFFLAAWPVIGIWFTALGVSTMAFNLNGFNFNQSVIDSQGRVINTWADIINRANLGMEVMHERNAHNFPLDLAAGEQAPVALTAPAING

>tr|K9TQ26|K9TQ26_9CYAN Photosystem II protein D1 OS=Oscillatoria acuminata PCC 6304 OX=56110 GN=psbA PE=3 SV=1

MTTTLQQRESANLWDRFCEWVASTENRLYIGWFGVLMIPTLLSATVCYIIAFIAAPPVDIDGIREPVAGSLLYGNNIISGAVVPSSNAIGLHFYPIWEAASLDEWLYNGGPYQLVIFHFLIGIFCYMGREWELSYRLGMRPWICVAYSAPVAAASAVFLIYPIGQGSFSDGMPLGISGTFNFMLVFQAEHNILMHPFHMLGVAGVFGGSLFSAMHGSLVTSSLVRETSETESQNYGYKFGQEEETYNIVAAHGYFGRLIFQYASFNNSRSLHFFLAAWPVVGIWFTALGVSTMAFNLNGFNFNQSIIDSTGRVVNTWADVINRANLGMEVMHERNAHNFPLDLAAGEATPVALTAPSING

>tr|K9QUF0|K9QUF0_NOSS7 Photosystem II protein D1 OS=Nostoc sp. (strain ATCC 29411 / PCC 7524) OX=28072 GN=psbA PE=3 SV=1

MTTTLQQRKSANVWEQFCEWITSTDNRIYIGWFGVLMIPTLLAATTCFIIAFIAAPPVDIDGIREPVAGSLIYGNNIISGAVVPSSNAIGLHFYPIWEAASLDEWLYNGGPYQLVIFHFLIGCACYLGRQWELSYRLGMRPWICVAYSAPLASATAVFLIYPIGQGSFSDGMPLGISGTFNFMIVFQAEHNILMHPFHMLGVAGVFGGSLFSAMHGSLVTSSLVRETTETESQNYGYKFGQEEETYNIVAAHGYFGRLIFQYASFNNSRSLHFFLAAWPVVGIWFTALGISTMAFNLNGFNFNQSVIDSQGRVINTWADIINRANLGMEVMHERNAHNFPLDLAAGEVAPVALTAPAING

>tr|A0A522XDT2|A0A522XDT2_9CYAN Photosystem II protein D1 OS=Phormidium sp. SL48-SHIP OX=2518363 GN=psbA PE=3 SV=1

MTTTLQQQQSASLWERFCGWVTSTNNRLYIGWFGVLMIPTLLTATTCFIIAFIAAPPVDIDGIREPVAGSLLYGNNIISGAVVPSSNAIGLHLYPIWEAASLDEWLYNGGPYQLVILHFLIGVFCYMGREWELSFRLGMRPWICVAYSAPVAAATAVFLIYPIGQGSFSDGMPLGISGTFNFMLVFQAEHNILMHPFHMLGVAGVFGGALFSAMHGSLVTSSLVRETTESESQNYGYKFGQEEETYNIVAAHGYFGRLIFQYASFNNSRSLHFFLGAWPVVGIWFTALGISTMAFNLNGFNFNQSVLDSQGRVINTWADVINRANLGMEVMHERNAHNFPLDLAATEAPSING

>sp|A3PAU3|PSBA_PROM0 Photosystem II protein D1 OS=Prochlorococcus marinus (strain MIT 9301) OX=167546 GN=psbA PE=3 SV=1

MTTIQQQRSSLLKGWPQFCEWVTSTNNRIYVGWFGVLMIPCLLTAAACFIVAFIAAPPVDIDGIREPVAGSFLYGNNIISGAVVPSSNAIGLHFYPIWEAATVDEWLYNGGPYQLVIFHFLIGISAYMGRQWELSYRLGMRPWICVAYSAPVSAAFAVFLVYPFGQGSFSDGMPLGISGTFNFMFVFQAEHNILMHPFHMAGVAGMFGGSLFSAMHGSLVTSSLIRETTETESQNYGYKFGQEEETYNIVAAHGYFGRLIFQYASFNNSRSLHFFLAVFPVVCVWLTSMGICTMAFNLNGFNFNQSVVDANGKIVPTWGDVLNRANLGMEVMHERNAHNFPLDLAAAESTTVALSAPAIG

>tr|Q6YXN7|Q6YXN7_PHYPA Photosystem II protein D1 OS=Physcomitrella patens subsp. patens OX=3218 GN=psbA PE=3 SV=1

MTATLERRESASLWGRFCDWVTSTENRLYIGWFGVLMIPTLLTATSVFIIAFIAAPPVDIDGIREPVSGSLLYGNNIISAAIIPTSAAIGLHFYPIWEAASVDEWLYNGGPYELIVLHFLLGVACYMGREWELSYRLGMRPWIAVAYSAPVAAATAVFLIYPIGQGSFSDGMPLGISGTFNFMIVFQAEHNILMHPFHMLGVAGVFGGSLFSAMHGSLVTSSLIRETTENESANAGYKFGQEEETYNIVAAHGYFGRLIFQYASFNNSRSLHFFLAAWPVVGIWFTALGISTMAFNLNGFNFNQSVVDSQGRVINTWADIINRANLGMEVMHERNAHNFPLDLASVEAPSVNG

>tr|D4NY65|D4NY65_GALSU Photosystem II protein D1 OS=Galdieria sulphuraria OX=130081 GN=psbA PE=3 SV=1

MTATLERRQTASLWERFCSWITSTENRLYIGWFGVLMIPTLLTATSVFIIGFIAAPPVDIDGIREPVSGSLLYGNNIITGAIVPTSNAIGIHFYPIWEAASLDEWLYNGGPYELIVLHFFIGICAYMGREWELSYRLGMRPWIAVAFSAPVAAATAVFIIYPIGQGSFSDGMPLGISGTFNFMLVFQAEHNILMHPFHMMGVAGVFGGSLFSAMHGSLVTSSLIRETTENESANNGYKFGQEYETYNIVAAHGYFGRLIFQYASFNNSRSLHFFLALWPVVCIWLTSLGISTMAFNLNGFNFNQSVVDSQGRVINTWADILNRANLGIEVMHERNAHNFPLDLASEVSLPVALDKVEING

>tr|Q9MUA3|Q9MUA3_VAULI Photosystem II protein D1 OS=Vaucheria litorea OX=109269 GN=psbA PE=3 SV=1

MTATLERRESISLWERFCSWITSTDNRLYIGWFGVLMIPTLLTATTCYIIAFIAAPPVDIDGIREPVAGSLLYGNNIISGAVIPSSNAIGVHFYPIWEAASVDEWLYNGGPYQLIVLHFLLGVASYMGREWELSYRLGMRPWIFVAFSAPVAAASAVFLVYPIGQGSFSDGMPLGISGTFNFMLVFQAEHNILMHPFHMAGVAGVFGGSLFSAMHGSLVTSSLIRETSEVESTNYGYKFGQEEETYNIVAAHGYFGRLIFQYASFNNSRALHFFLAAWPVVGIWLTALGVSTMAFNLNGFNFNQSVVDSQGRVINTWADIINRADLGMEVMHERNAHNFPLDLAAGEILPVAVTAPVIAG

**Notes S2.** Amino acid sequences of D2 proteins

>YP_004324588.1 photosystem II D2 protein (photosystem q(a) protein) [Prochlorococcus phage Syn1]

MTTSTLTTPTRGWFDVLDDWVKRDRFVFVGWSGLLLLPTAYLAIGGWLTGTAFVTSWYTHGLASSYLEGANFLTAAVSTPADAMGHSLLLLWGPESQGDFQRWCQLGGLWNFVALHGAFALIGFMLRQFELARLIGIRPYNAIAFSGPIAVFVSVFLIYPLGQSSWFFAPSFGVAAIFRFLLFLQGFHNWTLNPFHMMGVAGILGGALLSAIHGVTVENTLYEDGEQANTFKAFDSTQEEETYSMVTANRFWSQIFGVAFSNKRWLHFFMLFVPVMGLWVSSIGIIGLALNLRAYDFVSQEIRAAEDPEFETFYTKNILLNEGLRAWLAPSDQPHENFIFPEEVLPRGNAL

>YP_004324039.1 photosystem II D2 protein (photosystem q(a) protein) [Synechococcus phage Syn19]

MVASTLSPPRRGWFDVLDDWLKRDRFVFVGWSGLLLFPTAYLAIGGWLTGTAFVTSWYTHGLASSYLEGANFLTAAVSTPADSMGHSLLLLWGPEAQGDFVRWCQLGGLWAFVALHGAFALIGFMLRQFELARLIGIRPYNAIAFSGPIAVFVSVFLIYPLGQSSWFFAPSFGVSAIFRFLLFLQGFHNWTLNPFHMMGVAGILGGALLSAIHGVTVENTLYQDGEQANTFKAFDSTQEEETYSMVTANRFWSQIFGIAFSNKRWLHFFMLFVPVMGLWTSSIGIIGLALNLRAYDFVSQEIRAAEDPEFETFYTKNILLNEGLRAWLAPIDQPHENFVFPEEVLPRGNAL

>YP_004323821.1 photosystem II D2 protein (photosystem q(a) protein) [Prochlorococcus phage Syn33]

MTTTTLKAPTRGWFDVLDDWLKRDRFVFIGWSGLLLLPTAYMAIGGWLTGTTFVTSWYTHGLATSYLEGANFLTAAVSTPADAMGHSLLLLWGPEAQGDFVRWIQLGGLWNFVALHGAFALIGFMLRQFELARLIGIRPYNAIAFSGPIAVFVSVFLIYPLGQSSWFFAPSFGVAAIFRFLLFLQGFHNWTLNPFHMMGVAGILGGALLSAIHGVTVENTLYEDGEQANTFKAFDSTQEEETYSMVTANRFWSQIFGVAFSNKRWLHFFMLFVPVMGLWTSSIGIIGLALNLRAYDFVSQEIRAAEDPEFETFYTKNILLNEGLRAWLAPVDQPGERFVFPEEVLPRGNAL

>YP_004323114.1 photosystem II D2 protein (photosystem q(a) protein) [Synechococcus phage S-SM1]

MVASTLSPPRRGWFDVLDDWLKRDRFVFVGWSGLLLFPTAYLAIGGWLTGTTFATSWYTHGLASSYLEGANFLTAAVSTPADAMGHSLLLLWGPEAQGDFVRWIQLGGLWAFVALHGAFALIGFMLRQFELARLIGIRPYNAIAFSGPIAVFVSVFLIYPLGQSSWFFAPSFGVAAIFRFLLFLQGFHNWTLNPFHMMGVAGILGGALLSAIHGVTVENTLYQDGDDANTFKAFDSTQEEETYSMVTANRFWSQIFGIAFSNKRWLHFFMLFVPVMGLWTSSIGIIGLALNLRAYDFVSQEIRAAEDPEFETFYTKNILLNEGLRAWLAPVDQPHENFVFPEEVLPRGNAL

>YP_004322878.1 photosystem II D2 protein (photosystem q(a) protein) [Synechococcus phage S-ShM2]

MTTSTLNIPTRGWFDVLDDWLKRDRFVFIGWSGLLLLPTAYLAIGGWLTGTAFVTSWYTHGLASSYLEGANFLTAAVSTPADAMGHSLLLLWGPEAQGDFVRWCQLGGLWNFVALHGAFALIGFMLRQFELARLIGIRPYNAIAFSGPIAVFVSVFLIYPLGQSSWFFAPSFGVSAIFRFLLFLQGFHNWTLNPFHMMGVAGILGGALLSAIHGVTVENTLYEDGEQANTFKAFDSTQEEETYSMVTANRFWSQIFGIAFSNKRWLHFFMLFVPVMGLWTSSIGIIGLALNLRAYDFVSQEIRAAEDPEFETFYTKNILLNEGLRAWLAPADQPHENFIFPEEVLPRGNAL

>YP_007006006.1 photosystem II D2 protein (photosystem q(a) protein) [Cyanophage S-TIM5]

MVASTLTQQSQRREWFDILDDWLKRDRFVFVGWSGLLLFPTAYLAIGGWLTGTTFVTSWYTHGLASSYLEGANFLTAAVSTPADAMGHSLLLLWGPESQGDFVRWIQLGGLWAFVALHGAFALIGFMLRQFELARLIGIRPYNAIAFSGPIAVFVSVFLIYPLGQSSWFFAPSFGVAAIFRFLLFLQGFHNWTLNPFHMMGVAGILGGALLSAIHGVTVENTLYQDGEQANTFKAFDSTQEEETYSMVTANRFWSQIFGIAFSNKRWLHFFMLFVPVMGLWTSSIGIIGLALNLRAYDFVSQEIRASEDPEFETFYTKNILLNEGLRAWMAPVDQPHESFVFPEEVLPRGNAL

>YP_009783353.1 photosystem II D2 protein (photosystem q(a) protein) [Cyanophage S-RIM44]

MTTSTLTTPTRGWFDVLDDWVKRDRFVFVGWSGLLLLPTAYLAIGGWLTGTAFVTSWYTHGLASSYLEGANFLTAAVSTPADAMGHSLLLLWGPESQGDFQRWCQLGGLWNFVALHGAFALIGFMLRQFELARLIGIRPYNAIAFSGPIAVFVSVFLIYPLGQSSWFFAPSFGVAAIFRFLLFLQGFHNWTLNPFHMMGVAGILGGALLSAIHGVTVENTLYEDGEQANTFKAFDSTQEEETYSMVTANRFWSQIFGVAFSNKRWLHFFMLFVPVMGLWVSSIGIIGLALNLRAYDFVSQEIRAAEDPEFETFYTKNILLNEGLRAWLAPSDQPHENFIFPEEVLPRGNAL

>YP_009783123.1 photosystem II D2 protein (photosystem q(a) protein) [Synechococcus phage S-RIM8]

MTTSTLQLPRRGWFDVLDDWLKRDRFVFVGWSGLLLFPTAYLALGGWLTGTTFVTSWYTHGLASSYLEGANFLTAAVSTPADAMGHSLLLLWGPESQGDFIRWCQLGGLWTFVALHGAFALIGFMLRQFELARLIGIRPYNAIAFSGPIAVFVSVFLIYPLGQSSWFFAPSFGVAAIFRFLLFLQGFHNWTLNPFHMMGVAGILGGALLSAIHGVTVENTLYEDGEQANTFKAFDSTQEEETYSMVTANRFWSQIFGIAFSNKRWLHFFMLFVPVMGLWTSSIGIIGLALNLRAYDFVSQEVRAAEDPEFETFYTKNILLNEGLRAWLAPVDQPHENFVFPEEVLPRGNAL

>YP_009324375.1 photosystem II D2 protein (photosystem q(a) protein) [Synechococcus phage S-WAM2]

MVASTLTLQKQRGWFDVLDDWLKRDRFVFVGWSGLLLFPTAYMALGGWLTGTTFVTSWYTHGIASSYLEGCNFLTAAVSTPADALGHSLLLLWGPEAQGDFVRWLQLGGLWPFVALHGAFALIGFMLRQFEIARLVGIRPYNAIAFSGPIAVFVSVFLMYPLGQSSWFFAPSFGVAAIFRFLLFLQGFHNWTLNPFHMMGVAGILGGALLCAIHGATVENTLFEDSDQSNTFKAFEPTQEEETYSMVTANRFWSQIFGIAFSNKRWLHFFMLFVPVMGLWTSSIGIIGLALNLRAYDFVSQEIRAAEDPEFETFYTKNILLNEGLRAWMAPVDQPHENFVFPEEVLPRGNAL

>YP_009322642.1 photosystem II D2 protein (photosystem q(a) protein) [Synechococcus phage S-CAM9]

MTTSTLNIPQRGWFDVLDDWLKRDRFVFVGWSGLLLFPTAYLAIGGWLTGTTFVTSWYTHGLASSYLEGANFLTAAVSTPADAMGHSLLLLWGPESQGDFIRWCQLGGLWAFVAFHGSFALIGFMLRQFEIARLVGIRPYNAIAFSGPIAVFVSVFLMYPLGQSSWFFAPSFGVAAIFRFLLFLQGFHNWTLNPFHMMGVAGILGGALLCAIHGATVENTLFEDGEQSNTFKAFEPTQEEETYSMVTANRFWSQIFGIAFSNKRWLHFFMLFVPVMGLWTSSIGIIGLALNLRAYDFVSQEIRAAEDPEFETFYTKNILLNEGLRAWMAPVDQPHESFVFPEEVLPRGNAL

>YP_009321484.1 photosystem II D2 protein (photosystem q(a) protein) [Synechococcus phage S-CAM3]

MVASTLQQPRREWFDILDDWLKRDRFVFVGWSGLLLFPTAYLAIGGWLTGTTFATSWYTHGLASSYLEGANFLTAAVSTPADAMGHSLLLLWGPESQGDFVRWIQLGGLWAFVALHGAFALIGFMLRQFELARLIGIRPYNAIAFSGPIAVFVSVFLIYPLGQSSWFFAPSFGVAAIFRFLLFLQGFHNWTLNPFHMMGVAGILGGALLSAIHGVTVENTLYEDGEQANTFKAFDSTQEEETYSMVTANRFWSQIFGVAFSNKRWLHFFMLFVPVMGLWTSSIGIIGLALNLRAYDFVSQEIRAAEDPEFETFYTKNILLNEGLRSWLAPVDQPHENFVFPEEVLPRGNAL

>YP_009302298.1 photosystem II D2 protein (photosystem q(a) protein) [Cyanophage S-RIM50]

MVASTLSPPRRGWFDVLDDWLKRDRFVFVGWSGLLLFPTAYLALGGWLTGTTFVTSWYTHGLASSYLEGANFLTAAVSTPADAMGHSLLLLWGPESQGDFIRWCQLGGLWTFVALHGAFALIGFMLRQFELARLIGIRPYNAIAFSGPIAVFVSVFLMYPLGQSSWFFAPSFGVAAIFRFLLFLQGFHNWTLNPFHMMGVAGILGGALLSAIHGVTVENTLYEDGEQANTFKAFDSTQEEETYSMVTANRFWSQIFGIAFSNKRWLHFFMLFVPVMGLWTSSIGIIGLALNLRAYDFVSQEIRAAEDPEFETFYTKNILLNEGLRAWLAPVDQPHENFVFPEEVLPRGNAL

>sp|P56761|PSBD_ARATH Photosystem II D2 protein OS=Arabidopsis thaliana OX=3702 GN=psbD PE=1 SV=3

MTIALGKFTKDEKDLFDIMDDWLRRDRFVFVGWSGLLLFPCAYFALGGWFTGTTFVTSWYTHGLASSYLEGCNFLTAAVSTPANSLAHSLLLLWGPEAQGDFTRWCQLGGLWAFVALHGAFALIGFMLRQFELARSVQLRPYNAIAFSGPIAVFVSVFLIYPLGQSGWFFAPSFGVAAIFRFILFFQGFHNWTLNPFHMMGVAGVLGAALLCAIHGATVENTLFEDGDGANTFRAFNPTQAEETYSMVTANRFWSQIFGVAFSNKRWLHFFMLFVPVTGLWMSALGVVGLALNLRAYDFVSQEIRAAEDPEFETFYTKNILLNEGIRAWMAAQDQPHENLIFPEEVLPRGNAL

>sp|P06005|PSBD_SPIOL Photosystem II D2 protein OS=Spinacia oleracea OX=3562 GN=psbD PE=1 SV=3

MTIAVGKFTKDEKDLFDSMDDWLRRDRFVFVGWSGLLLFPCAYFALGGWFTGTTFVTSWYTHGLASSYLEGCNFLTAAVSTPANSLAHSLLLLWGPEAQGDFTRWCQLGGLWAFVALHGAFALIGFMLRQFELARSVQLRPYNAIAFSGPIAVFVSVFLIYPLGQSGWFFAPSFGVAAIFRFILFFQGFHNWTLNPFHMMGVAGVLGAALLCAIHGATVENTLFEDGDGANTFRAFNPTQAEETYSMVTANRFWSQIFGVAFSNKRWLHFFMLFVPVTGLWMSALGVVGLALNLRAYDFVSQEIRAAEDPEFETFYTKNILLNEGIRAWMAAQDQPHENLIFPEEVLPRGNAL

>sp|Q8CM25|PSBD_THEEB Photosystem II D2 protein OS=Thermosynechococcus elongatus (strain BP-1) OX=197221 GN=psbD1 PE=1 SV=1

MTIAIGRAPAERGWFDILDDWLKRDRFVFVGWSGILLFPCAYLALGGWLTGTTFVTSWYTHGLASSYLEGCNFLTVAVSTPANSMGHSLLLLWGPEAQGDFTRWCQLGGLWTFIALHGAFGLIGFMLRQFEIARLVGVRPYNAIAFSAPIAVFVSVFLIYPLGQSSWFFAPSFGVAAIFRFLLFFQGFHNWTLNPFHMMGVAGVLGGALLCAIHGATVENTLFQDGEGASTFRAFNPTQAEETYSMVTANRFWSQIFGIAFSNKRWLHFFMLFVPVTGLWMSAIGVVGLALNLRSYDFISQEIRAAEDPEFETFYTKNLLLNEGIRAWMAPQDQPHENFVFPEEVLPRGNAL

>sp|P09192|PSBD_SYNY3 Photosystem II D2 protein OS=Synechocystis sp. (strain PCC 6803 / Kazusa) OX=1111708 GN=psbD PE=1 SV=2

MTIAVGRAPVERGWFDVLDDWLKRDRFVFIGWSGLLLFPCAFMALGGWLTGTTFVTSWYTHGLASSYLEGANFLTVAVSSPADAFGHSLLFLWGPEAQGNLTRWFQIGGLWPFVALHGAFGLIGFMLRQFEISRLVGIRPYNAIAFSGPIAVFVSVFLMYPLGQSSWFFAPSFGVAGIFRFILFLQGFHNWTLNPFHMMGVAGILGGALLCAIHGATVENTLFEDGEDSNTFRAFEPTQAEETYSMVTANRFWSQIFGIAFSNKRWLHFFMLFVPVTGLWMSSVGIVGLALNLRAYDFVSQELRAAEDPEFETFYTKNILLNEGMRAWMAPQDQPHENFIFPEEVLPRGNAL

>sp|P11005|PSBD_SYNE7 Photosystem II D2 protein OS=Synechococcus elongatus (strain PCC 7942) OX=1140 GN=psbD1 PE=3 SV=2

MTIAVGRAPAERGWFDVLDDWLKRDRFVFVGWSGLLLFPCAYLALGGWLTGTSFVTSWYTHGIASSYLEGGNFLTVAVSTPADAFGHSLMLLWGPEAQGNFVRWCQLGGLWNFVALHGAFGLIGFMLRQFEIARLVGVRPYNAIAFSGPIAVFVSVFLMYPLGQSSWFFAPSFGVAAIFRFLLFLQGFHNWTLNPFHMMGVAGILGGALLCAIHGATVENTLFEDSEQSNTFRAFEPTQAEETYSMVTANRFWSQIFGIAFSNKRWLHFFMLFVPVTGLWMSSIGIVGLALNLRAYDFVSQELRAAEDPEFETFYTKNILLNEGIRAWMAPQDQPHEKFVFPEEVLPRGNAL

>sp|P31557|PSBD_EUGGR Photosystem II D2 protein OS=Euglena gracilis OX=3039 GN=psbD PE=3 SV=1

MTFTDLNTENKNGWFDVADDWLKKDRFIFIGWSGLLLFPCSYLALGGWLTGITFVTSWYTHGLASSFLEGCNALTAAVSTPPNSMGHSLLLLLGSEAQWDFTRWLQIGGLWPFIALHGAFGLIGFMLRQFEIAKAVQIRPYNAIAFSAPISVFVSVFLIYPLGQSGWFFAPSFGVAAIFRFILFFQGFHNWTLNPFHMMGVAGVLGAALLCAIHGATVENTIFEDGDSPNTFRAFNPLQSEETYSMVTANRFWSQIFGVAFSNKRWLHFFMVFVPVTGLRMSALGIVGLALNLRAYDFVSQEIRAAEDPEFETFYTKNILLNEGIRAWMAAQDQPHEQFIFPEEVLPRGNAL

>sp|D0VWR8|PSBD_THEVL Photosystem II D2 protein (Fragment) OS=Thermosynechococcus vulcanus OX=32053 GN=psbD PE=1 SV=2

ERGWFDILDDWLKRDRFVFVGWSGILLFPCAYLALGGWLTGTTFVTSWYTHGLASSYLEGCNFLTVAVSTPANSMGHSLLLLWGPEAQGDFTRWCQLGGLWTFIALHGAFGLIGFMLRQFEIARLVGVRPYNAIAFSAPIAVFVSVFLIYPLGQSSWFFAPSFGVAAIFRFLLFFQGFHNWTLNPFHMMGVAGVLGGALLCAIHGATVENTLFQDGEGASTFRAFNPTQAEETYSMVTANRFWSQIFGIAFSNKRWLHFFMLFVPVTGLWMSAIGVVGLALNLRSYDFISQEIRAAEDPEFETFYTKNLLLNEGIRAWMAPQDQPHENFVFPEEVLPRGNAL

>sp|Q2VEI1|PSBD_SOLTU Photosystem II D2 protein OS=Solanum tuberosum OX=4113 GN=psbD PE=3 SV=1

MTIAIGKFTKDENDLFDIMDDWLRRDRFVFVGWSGLLLFPCAYFAVGGWFTGTTFVTSWYTHGLASSYLEGCNFLTAAVSTPANSLAHSLLLLWGPEAQGDFTRWCQLGGLWTFVALHGAFGLIGFMLRQFELARSVQLRPYNAIAFSGPIAVFVSVFLIYPLGQSGWFFAPSFGVAAIFRFILFFQGFHNWTLNPFHMMGVAGVLGAALLCAIHGATVENTLFEDGDGANTFRAFNPTQAEETYSMVTANRFWSQIFGVAFSNKRWLHFFMLFVPVTGLWMSALGVVGLALNLRAYDFVSQEIRAAEDPEFETFYTKNILLNEGIRAWMAAQDQPHENLIFPEEVLPRGNAL

>sp|Q2MIA5|PSBD_SOLLC Photosystem II D2 protein OS=Solanum lycopersicum OX=4081 GN=psbD PE=3 SV=1

MTIAIGKFTKDENDLFDIMDDWLRRDRFVFVGWSGLLLFPCAYFAVGGWFTGTTFVTSWYTHGLASSYLEGCNFLTAAVSTPANSLAHSLLLLWGPEAQGDFTRWCQLGGLWTFVALHGAFGLIGFMLRQFELARSVQLRPYNAIAFSGPIAVFVSVFLIYPLGQSGWFFAPSFGVAAIFRFILFFQGFHNWTLNPFHMMGVAGVLGAALLCAIHGATVENTLFEDGDGANTFRAFNPTQAEETYSMVTANRFWSQIFGVAFSNKRWLHFFMLFVPVTGLWMSALGVVGLALNLRAYDFVSQEIRAAEDPEFETFYTKNILLNEGIRAWMAAQDQPHENLIFPEEVLPRGNAL

>sp|P06006|PSBD_PEA Photosystem II D2 protein OS=Pisum sativum OX=3888 GN=psbD PE=1 SV=5

MTIALGKFTKDQNDLFDIMDDWLRRDRFVFVGWSGLLLFPCAYFAVGGWFTGTTFVTSWYTHGLASSYLEGCNFLTAAVSTPANSLAHSLLLLWGPEAQGDLTRWCQLGGLWTFVALHGAFGLIGFMLRQFELARSVQLRPYNAIAFSGPIAVFVSVFLIYPLGQSGWFFAPSFGVAAIFRFILFFQGFHNWTLNPFHMMGVAGVLGAALLCAIHGATVENTLFEDGDGANTFRAFNPTQAEETYSMVTANRFWSQIFGVAFSNKRWLHFFMLFVPVTGLWMSALGVVGLALNLRAYDFVSQEIRAAEDPEFETFYTKNILLNEGIRAWMATQDQPHENLIFPEEVLPRGNAL

>sp|P69686|PSBD_TOBAC Photosystem II D2 protein OS=Nicotiana tabacum OX=4097 GN=psbD PE=3 SV=1

MTIALGKFTKDENDLFDIMDDWLRRDRFVFVGWSGLLLFPCAYFAVGGWFTGTTFVTSWYTHGLASSYLEGCNFLTAAVSTPANSLAHSLLLLWGPEAQGDFTRWCQLGGLWTFVALHGAFGLIGFMLRQFELARSVQLRPYNAIAFSGPIAVFVSVFLIYPLGQSGWFFAPSFGVAAIFRFILFFQGFHNWTLNPFHMMGVAGVLGAALLCAIHGATVENTLFEDGDGANTFRAFNPTQAEETYSMVTANRFWSQIFGVAFSNKRWLHFFMLFVPVTGLWMSALGVVGLALNLRAYDFVSQEIRAAEDPEFETFYTKNILLNEGIRAWMAAQDQPHENLIFPEEVLPRGNAL

>sp|Q36814|PSBD_WHEAT Photosystem II D2 protein OS=Triticum aestivum OX=4565 GN=psbD PE=3 SV=2

MTIALGRIPKEENDLFDTMDDWLRRDRFVFVGWSGLLLFPCAYFALGGVFTGTTFVTSWYTHGVASSYLEGCNFLTAAVSTPANSLAHSLLLLWGPEAQGDFTRWCQLGGLWTFVALHGAFALIGFMLRQFELARSVQLRPYNAISFSGPIAVFVSVFLIYPLGQSGWFFAPSFGVAAIFRFILFFQGFHNWTLNPFHMMGVAGVLGAALLCAIHGATVENTLFEDGDGANTFRAFNPTQAEETYSMVTANRFWSQIFGVAFSNKRWLHFFMLFVPVTGLWMSAIGVVGLALNLRAYDFVSQEIRAAEDPEFETFYTKNILLNEGIRAWMAAQDQPHENLIFPEEVLPRGNAL

>sp|Q8XFA5|PSBD_NOSS1 Photosystem II D2 protein OS=Nostoc sp. (strain PCC 7120 / SAG 25.82 / UTEX 2576) OX=103690 GN=psbD1 PE=3 SV=1

MTIAVGRAPSRGWFDVLDDWLKRDRFVFVGWSGILLFPCAFLALGGWLTGTTFVTSWYTHGLASSYLEGANFLTVAVSSPADSMGHSLLLLWGPEAQGDLTRWFQLGGLWPFVALHGAFGLIGFMLRQFEIARLVGIRPYNALAFSAPIAVFVSVFLMYPLGQSSWFFAPSFGVAAIFRFLLFLQGFHNWTLNPFHMMGVAGVLGGALLCAIHGATVENTLFEDGEGANTFRAFNPTQSEETYSMVTANRFWSQIFGIAFSNKRWLHFFMLFVPVTGLWMSAVGIVGLALNLRAYDFVSQELRAAEDPEFETFYTKNILLNEGIRAWMAPQDQPHEKFVFPEEVLPRGNAL

>sp|P06007|PSBD_CHLRE Photosystem II D2 protein OS=Chlamydomonas reinhardtii OX=3055 GN=psbD PE=1 SV=1

MTIAIGTYQEKRTWFDDADDWLRQDRFVFVGWSGLLLFPCAYFALGGWLTGTTFVTSWYTHGLATSYLEGCNFLTAAVSTPANSMAHSLLFVWGPEAQGDFTRWCQLGGLWAFVALHGAFGLIGFMLRQFEIARSVNLRPYNAIAFSAPIAVFVSVFLIYPLGQSGWFFAPSFGVAAIFRFILFFQGFHNWTLNPFHMMGVAGVLGAALLCAIHGATVENTLFEDGDGANTFRAFNPTQAEETYSMVTANRFWSQIFGVAFSNKRWLHFFMLLVPVTGLWMSAIGVVGLALNLRAYDFVSQEIRAAEDPEFETFYTKNILLNEGIRAWMAAQDQPHERLVFPEEVLPRGNAL

>tr|D0VMT7|D0VMT7_VOLCA Photosystem II D2 protein OS=Volvox carteri f. nagariensis OX=3068 GN=psbD PE=3 SV=1

MTIAIGTYQEKRTWFDDADDWLRQDRFVFVGWSGLLLFPCAYFALGGWLTGTTFVTSWYTHGLASSYLEGCNFLTAAVSTPANSMAHSLLFVWGPEAQGDFTRWCQLGGLWTFVALHGAFGLIGFMLRQFEIARSVNLRPYNAIAFSAPIAVFVSVFLIYPLGQSGWFFAPSFGVAAIFRFILFFQGFHNWTLNPFHMMGVAGVLGAALLCAIHGATVENTLFEDGDGANTFRAFNPTQAEETYSMVTANRFWSQIFGVAFSNKRWLHFFMLLVPVTGLWMSALGVVGLALNLRAYDFVSQEIRAAEDPEFETFYTKNILLNEGIRAWMAAQDQPHERLVFPEEVLPRGNAL

>sp|B1WQ89|PSBD_CROS5 Photosystem II D2 protein OS=Crocosphaera subtropica (strain ATCC 51142 / BH68) OX=43989 GN=psbD1 PE=3 SV=2

MTIAVGRAPERGWFDVLDDWLKRDRFVFVGWSGLLLFPCAYLALGGWLTGTTFVTSWYTHGLASSYLEGCNFLTVAVSSPANAFGHSLLFLWGPEAQGDFTRWCQIGGLWTFTALHGAFGLIGFMLRQFEIARLVGIRPYNAIAFSAPIAVFVSVFLMYPLGQSSWFFGPSFGVAGIFRFILFLQGFHNWTLNPFHMMGVAGVLGGALLCAIHGATVENTLFEDGEQANTFRAFEPTQAEETYSMVTANRFWSQIFGIAFSNKRWLHFFMLFVPVTGLWMSAIGIVGLALNLRAYDFVSQELRAAEDPEFETFYTKNILLNEGLRAWMAPQDQPHQNFVFPEEVLPRGNAL

>tr|Q7NI61|Q7NI61_GLOVI Photosystem II D2 protein OS=Gloeobacter violaceus (strain ATCC 29082 / PCC 7421) OX=251221 GN=psbD PE=3 SV=1

MTIAVGRSEQSQGWFDALDDWLKKDRFVFIGWSGLLFFPTAYLAVGAWLTGTTFVTSWFTHGLASSYLEGGNFLTVAVSSPADAMGHSLLLLWGPEAQGDFTRWCQIGGLWAFISFHGALALMGFMLRQFEIARLIGIRPYNAIAFSAPIAVFVSVFLMYPLGQHSWFFGPSYGVNGIFRFLLFFQGFHNWTLNPFHMMGVAGVLGGALLCAIHGATVENTLFEDGEAPNTFKAFDPAQEEETYSMILANRFWSQIFGIAFSNKRWLHFFMLFVPVTGLWMASIGIIGVALNLRAYEFVSQEIRAAQDPEFETLYTANILINEGIRAWMGPYDQNYDETLKFPEEVLPRGNAL

>tr|K9S8C9|K9S8C9_9CYAN Photosystem II D2 protein OS=Geitlerinema sp. PCC 7407 OX=1173025 GN=psbD PE=3 SV=1

MTIAVGRAQAQRGWFDVLDDWLKRDRFVFIGWSGLLLFPCAYLAVGGWLTGTTFVSSWYTHGLASSYLEGCNFLTVAVSTPPNSLGHSLLFLWGPEAQGDFVRWCQLGGLWTFVALHGAFGLIGFMLRQFEISRLVGIRPYNAIAFSAPIAVFVSVFLMYPLGQSGWFFAPSFGVAAIFRFLLFFQGFHNWTLNPFHMMGVAGVLGGALLCAIHGATVENTLFEDGENANTFRAFNPTQAEETYSMVTANRFWSQIFGIAFSNKRWLHFFMLFVPVTGLWMSSVGIIGLGLNLRAYDFVSQEIRAAEDPEFETFYTKNILLNEGIRAWLAPQDQPHEGFAFPEEVLPRGNAL

>sp|A4GYQ4|PSBD_POPTR Photosystem II D2 protein OS=Populus trichocarpa OX=3694 GN=psbD PE=3 SV=1

MTIALGKFTKDENDLFDIMDDWLRRDRFVFVGWSGLLLFPCAYFALGGWFTGTTFVTSWYTHGLASSYLEGCNFLTAAVSTPANSLAHSLLLLWGPEAQGDFTRWCQLGGLWTFVALHGAFGLIGFMLRQFELARSVQLRPYNAIAFSGPIAVFVSVFLIYPLGQSGWFFAPSFGVAAIFRFILFFQGFHNWTLNPFHMMGVAGVLGAALLCAIHGATVENTLFEDGDGANTFRAFNPTQAEETYSMVTANRFWSQIFGVAFSNKRWLHFFMLFVPVTGLWMSALGVVGLALNLRAYDFVSQEIRAAEDPEFETFYTKNILLNEGIRAWMAAQDQPHENLIFPEEVLPRGNAL

>tr|U5QM98|U5QM98_9CYAN Photosystem II D2 protein OS=Gloeobacter kilaueensis JS1 OX=1183438 GN=psbD PE=3 SV=1

MTIAVGRSEQSQGWFDALDDWLKKDRFVFIGWSGLLFFPTAYFAVGGWLTGTTFVSSWYTHGLASSYLEGANFLTSAVSSPADAMGHSLLLLWGPEAQGDFTRWCQIGGLWAFIAFHGALALMGFMLRQFEIARLIGIRPYNAIAFSAPIAVFVSVFLMYPLGQHSWFFGPSFGVNGIFRFLLFVQGFHNWTLNPFHMMGVAGVLGGALLCAIHGATVENTLFEDGEAPNTFKAFDPAQEEETYSMVLANRFWSQIFGIAFSNKRWLHFFMLFVPVTGLWMASIGFVGLALNLRAYEFISQESRAAQDPEFETLYTANILINEGIRAWMGPYDQNYDETLKFPEEVLPRGNAL

>tr|A0A1V4BL54|A0A1V4BL54_MICAE Photosystem II D2 protein OS=Microcystis aeruginosa KW OX=1960155 GN=psbD PE=3 SV=1

MTIAVGRAPERGLFDALDDWLKRDRFVFIGWSGLLLFPCAFMALGGWLTGTTFVTSWYTHGLASSYLEGGNFLTVAVSTPADAFGHSILFLWGPEAQGNFTRWCQIGGLWPFVALHGAFGLIGFMLRQFEIARLVGIRPYNALAFSGPIAVFVSVFLMYPLGQSSWFFAPSFGVAGIFRFILFFQGFHNWTLNPFHMMGVAGILGGALLCAIHGATVENTLFEDGEGSNTFRAFEPTQAEETYSMVTANRFWSQIFGIAFSNKRWLHFFMLFVPVTGLWMSAVGVVGLALNLRAYDFVSQELRAAEDPEFETFYTKNILLNEGLRAWMAPQDQPHENFIFPEEVLPRGNAL

>tr|K9THR6|K9THR6_9CYAN Photosystem II D2 protein OS=Oscillatoria acuminata PCC 6304 OX=56110 GN=psbD PE=3 SV=1

MTIAVGRAQAERGWFDVLDDWLKRDRFVFIGWSGLLLFPCAYLAVGGWLTGTTFVTSWYTHGLASSYLEGCNFLTVAVSTPPNSLGHSLLFLWGPEAQGDFTRWFQLGGLWTFVALHGAFGLIGFCLRQLEIARLLGIRPYNGLAFTGPIAVFVSVFLIYPLGQSGWFFAPSFGVAAIFRFLLFFQGFHNWTLNPFHMMGVAGILGGALLCAIHGATVENTLFEDGEGSNTFRAFEPTQAEETYSMVTANRFWSQIFGIAFSNKRWLHFFMLFVPVTGLWMSAIGVVGLGLNLRAYDFVSQELRAAEDPEFETFYTKNILLNEGIRAWMAPVDQPHENFEFPEEVLPRGNAL

>tr|K9QMT7|K9QMT7_NOSS7 Photosystem II D2 protein OS=Nostoc sp. (strain ATCC 29411 / PCC 7524) OX=28072 GN=psbD PE=3 SV=1

MTIAVGRAPSRGWFDVLDDWLKRDRFVFVGWSGILLFPCAFLALGGWLTGTTFVTSWYTHGLASSYLEGCNFLTVAVSSPADSMGHSLLLLWGPEAQGDFTRWCQLGGLWPFVALHGAFGLIGFMLRQFEIARLVGIRPYNALAFSAPIAVFVSVFLMYPLGQSSWFFAPSFGVAAIFRFLLFLQGFHNWTLNPFHMMGVAGVLGGALLCAIHGATVENTLFEDGDGANTFRAFNPTQAEETYSMVTANRFWSQIFGIAFSNKRWLHFFMLFVPVTGLWMSAVGIVGLALNLRAYDFVSQELRAAEDPEFETFYTKNILLNEGIRAWMAPQDQPHEKFVFPEEVLPRGNAL

>tr|A3PDZ7|A3PDZ7_PROM0 Photosystem II D2 protein OS=Prochlorococcus marinus (strain MIT 9301) OX=167546 GN=psbD PE=3 SV=1

MTIAVGSAPQRGWFDVLDDWLKRDRFVFIGWSGLLLLPCAYLAIGGWFVGTTFVTSWYTHGVASSYLEGCNFLTAAVSTPGDAMGHSLLFLWGPEAQGSFVRWLQLGGLWNFVALHGVFGLIGFMLRQFEIAGLVGIRPYNALAFSAVIAVFTSIFLIYPLGQHSWFFAPSFGVAAIFRYILFIQGFHNITLNPFHMMGVAGILGGALLCAIHGATVQNTLYEDTSIYTDGKVQSSTFRAFDPTQEEETYSMITANRFWSQIFGIAFSNKRFLHFLMLFVPVMGMWTSSIGIVGLALNLRAYDFVSQEIRAAEDPEFETFYTKNILLNEGMRAWMSSVDQPHENFVFPEEVLPRGNAL

>sp|Q6YXN8|PSBD_PHYPA Photosystem II D2 protein OS=Physcomitrella patens subsp. patens OX=3218 GN=psbD PE=3 SV=1

MTIAIGKSSKEPKGLFDSMDDWLRRDRFVFVGWSGLLLFPCAYFSLGGWFTGTTFVTSWYTHGLASSYLEGCNFLTAAVSTPANSLAHSLLLLWGPEAQGDFTRWCQLGGLWTFVALHGAFALIGFMLRQFELARSVQLRPYNAIAFSGPIAVFVSVFLIYPLGQSGWFFAPSFGVAAIFRFILFFQGFHNWTLNPFHMMGVAGVLGAALLCAIHGATVENTLFEDGDGANTFRAFNPTQSEETYSMVTANRFWSQIFGVAFSNKRWLHFFMLFVPVTGLWMSAIGVVGLALNLRAYDFVSQEIRAAEDPEFETFYTKNILLNEGIRAWMAAQDQPHENLVFPEEVLPRGNAL

>sp|P28253|PSBD_GALSU Photosystem II D2 protein OS=Galdieria sulphuraria OX=130081 GN=psbD PE=3 SV=1

MTIAIERNIQRGLFDLVDDWLKRDRFVFIGWSGLLLFPCSYLALGAWFTGTTFVTSWYTHGLASSYLEGCNFLTAAVSSPANSMGHSLLFLWGPEAQGDFTRWCQIGGLWTFTALHGAFGLIGFCLRQFEIARLVGIRPYNAIAFSGPIAVFVSVFLIYPLGQASWFFAPSFGVAAIFRFILFLQGFHNWTLNPFHMMGVAGILGGALLCAIHGATVENTLFEDGEAANTFRAFTPTQSEETYSMVTANRFWSQIFGVAFSNKRWLHFFMLFVPVTGLWTSSIGIIGLALNLRAYDFVSQELRAAEDPEFETFYTKNLLLNEGIRAWMATQDQPHENFVFPEEVLPRGNAL

>tr|B7T1T0|B7T1T0_VAULI Photosystem II D2 protein OS=Vaucheria litorea OX=109269 GN=psbD PE=3 SV=1

MTIAIGQNQERGIFDLIDDWLKRDRFVFIGWSGLLLFPTAYLSLGGWFTGTTFVTSWYTHGLASSYLEGCNFLTAAVSSPANSMGHSLILLWGPEAQGDFTRWCQIGGLWAFIALHGSFALIGFCLRQFEIARLVGIRPYNAIAFSGPISVFLSVFLLYPLGQASWFFAPSFGVAAIFRFLLFLQGFHNWTLNPFHMMGVAGILGGALLCAIHGATVENTLFEDGDAANTFRAFTPTQSEETYSMVTANRFWSQIFGIAFSNKRWLHFFMLFVPVTGLWTSSIGIVGLALNLRAYDFVSQELRAAEDPEFETFYTKNILLNEGIRAWMAAQDQPHENFVFPEEVLPRGNAL

**Notes S3.** Ancestral sequences I_D1_ and II_D1_ predicted on the basis of the reconstructed phylogenetic tree for the D1 protein (Fig. 1A in the main text)

**I_D1_**

psbA (D1) ancestral sequence - node 82

MIPTLLTATVCFIIAFIAAPPVDIDGIREPVAGSLMYGNNIISGAVVPSSNAIGLHFYPIWEAASLDEWLYNGGPYQLVVFHFLIGIFCYMGREWELSYRLGMRPWICVAYSAPVAAASAVFLIYPIGQGSFSDGMPLGISGTFNFMLVFQAEHNILMHPFHMLGVAGVFGGSLFSAMHGSLVTSSLVRETTETESQNYGYKFGQEEETYNIVAAHGYFGRLIFQYASFNNSRSLHFFLAAWPVVGIWFTALGVSTMAFNLNGFNFNQSIISQGRVINTWADVLNRANLGMEVMHERNAHNFPLDLA

**II_D1_**

>psbA (D1) ancestral sequence - node 79 MIPTLLTATVCFIVAFIAAPPVDIDGIREPVAGSLMYGNNIISGAVVPSSNAIGLHFYPIWEAASLDEWLYNGGPYQLVVFHFLIGISCYMGRQWELSYRLGMRPWICVAYSAPVSAAFAVFLIYPIGQGSFSDGMPLGISGTFNFMFVFQAEHNILMHPFHMLGVAGVFGGSLFSAMHGSLVTSSLVRETTETESQNYGYKFGQEEETYNIVAAHGYFGRLIFQYASFNNSRSLHFFLAAWPVVGIWFTSMGISTMAFNLNGFNFNQSVLSQGKVINTWADVLNRANLGMEVMHERNAHNFPLDLA

**Notes S4.** Ancestral sequences I_D2_ and II_D2_ predicted on the basis of the reconstructed phylogenetic tree for the D2 protein (Fig. 1B in the main text)

**I_D2_**

>psbD (D2) ancestral sequence - node 62

MRGWFDVLDDWLKRDRFVFVGWSGLLLFPCAYLALGGWLTGTTFVTSWYTHGLASSYLEGCNFLTVAVSSPADAMGHSLLLLWGPEAQGDFTRWCQLGGLWTFVALHGAFGLIGFMLRQFEIARLVGIRPYNAIAFSGPIAVFVSVFLMYPLGQSSWFFAPSFGVAAIFRFLLFLQGFHNWTLNPFHMMGVAGILGGALLCAIHGATVENTLFEDGEQSNTFRAFEPTQAEETYSMVTANRFWSQIFGIAFSNKRWLHFFMLFVPVTGLWMSSIGIVGLALNLRAYDFVSQELRAAEDPEFETFYTKNILLNEGIRAWMAPQDQPHENFVFPEEVLPRGNAL

**II_D1_**

>psbD (D2) ancestral sequence - node 65

MRGWFDVLDDWLKRDRFVFVGWSGLLLFPTAYLALGGWLTGTTFVTSWYTHGLASSYLEGCNFLTVAVSSPADAMGHSLLLLWGPEAQGDFTRWCQLGGLWTFVALHGAFALIGFMLRQFEIARLVGIRPYNAIAFSGPIAVFVSVFLMYPLGQSSWFFAPSFGVAAIFRFLLFLQGFHNWTLNPFHMMGVAGILGGALLCAIHGATVENTLFEDGEQSNTFKAFEPTQEEETYSMVTANRFWSQIFGIAFSNKRWLHFFMLFVPVTGLWMSSIGIIGLALNLRAYDFVSQEIRAAEDPEFETFYTKNILLNEGIRAWMAPQDQPHENFVFPEEVLPRGNAL

**Notes S5.** The sequence for D1 protein and alignment of the template sequence for the D1 protein (PDB, 6v1p.1.U) and the predicted model for D1. The green colour indicates the sequence used as template for D1 protein. The yellow colour indicates amino acids that are only present in the template, the blue colour indicates amino acids in both the template and the model D1 that are involved in the binding of the Mn_4_O_5_Ca cluster

**D1 protein**

**>sp|P0A444|PSBA1_THEVB Photosystem II protein D1 1 OS=Thermosynechococcus vestitus; T. vulcanus(strain IAM M-273 / NIES-2133 / BP-1) OX=197221 GN=psbA1 PE=1 SV=1**

**11**

**1 MTTTLQRRESANLWERFCNWVTSTDNRLYVGWFGVIMIPTLLAATICFVIAFIAAPPVDI 60**

**61 DGIREPVSGSLLYGNNIITGAVVPSSNAIGLHFYPIWEAASLDEWLYNGGPYQLIIFHFL 120**

**121 LGASCYMGRQWELSYRLGMRPWICVAYSAPLASAFAVFLIYPIGQGSFSDGMPLGISGTF 180**

**181 NFMIVFQAEHNILMHPFHQLGVAGVFGGALFCAMHGSLVTSSLIRETTETESANYGYKFG 240**

**241 QEEETYNIVAAHGYFGRLIFQYASFNNSRSLHFFLAAWPVVGVWFTALGISTMAFNLNGF 300**

**301 NFNHSVIDAKGNVINTWADIINRANLGMEVMHERNAHNFPLDLASAESAPVAMIAPSING 360**

**344**

**6v1p.1.U (template) 11 ANLWERFCNWVTSTDNRLYVGWFGVIMIPTLLAATICFVIAFIAAPPVDI 60**

**||||||.||:||::||||||||||**

**model_D1 11 --------------------------MIPTLLTATVCFIVAFIAAPPVDI 34**

**6v1p.1.U (template) 61 DGIREPVSGSLLYGNNIITGAVVPSSNAIGLHFYPIWEAASLDEWLYNGG 110**

**|||||||:|||:||||||:|||||||||||||||||||||||||||||||**

**model_D1 35 DGIREPVAGSLMYGNNIISGAVVPSSNAIGLHFYPIWEAASLDEWLYNGG 84**

**6v1p.1.U (template) 111 PYQLIIFHFLLGASCYMGRQWELSYRLGMRPWICVAYSAPLASAFAVFLI 160**

**||||::||||:|.|||||||||||||||||||||||||||:::|||||||**

**model_D1 85 PYQLVVFHFLIGISCYMGRQWELSYRLGMRPWICVAYSAPVSAAFAVFLI 134**

**6v1p.1.U (template) 161 YPIGQGSFSDGMPLGISGTFNFMIVFQAEHNILMHPFHQLGVAGVFGGAL 210**

**|||||||||||||||||||||||.||||||||||||||.|||||||||:|**

**model_D1 135 YPIGQGSFSDGMPLGISGTFNFMFVFQAEHNILMHPFHMLGVAGVFGGSL 184**

**6v1p.1.U (template) 211 FCAMHGSLVTSSLIRETTETESANYGYKFGQEEETYNIVAAHGYFGRLIF 260**

**|.|||||||||||:||||||||.|||||||||||||||||||||||||||**

**model_D1 185 FSAMHGSLVTSSLVRETTETESQNYGYKFGQEEETYNIVAAHGYFGRLIF 234**

**6v1p.1.U (template) 261 QYASFNNSRSLHFFLAAWPVVGVWFTALGISTMAFNLNGFNFNHSVIDAK 310**

**||||||||||||||||||||||:|||::|||||||||||||||.||: ::**

**model_D1 235 QYASFNNSRSLHFFLAAWPVVGIWFTSMGISTMAFNLNGFNFNQSVL-SQ 283**

**6v1p.1.U (template) 311 GNVINTWADIINRANLGMEVMHERNAHNFPLDLA 344**

**|.|||||||::|||||||||||||||||||||||**

**model_D1 284 GKVINTWADVLNRANLGMEVMHERNAHNFPLDLA 317**

**Notes S6.** The sequence for D2 protein and alignment of the template sequence for the D2 protein (PDB, 6dhe.1.D) and the predicted model for D2. The green colour indicates the sequence used as template for D2 protein. The blue colour indicates similar amino acids in both the template and the model D2

**D2 protein**

**>sp|Q8CM25|PSBD_THEVB Photosystem II D2 protein OS=Thermosynechococcus vestitus; T. vulcanus (strain IAM M-273 / NIES-2133 / BP-1) OX=197221 GN=psbD1 PE=1 SV=1**

**12**

**1 MTIAIGRAPAERGWFDILDDWLKRDRFVFVGWSGILLFPCAYLALGGWLTGTTFVTSWYT 60**

**61 HGLASSYLEGCNFLTVAVSTPANSMGHSLLLLWGPEAQGDFTRWCQLGGLWTFIALHGAF 120**

**121 GLIGFMLRQFEIARLVGVRPYNAIAFSAPIAVFVSVFLIYPLGQSSWFFAPSFGVAAIFR 180**

**181 FLLFFQGFHNWTLNPFHMMGVAGVLGGALLCAIHGATVENTLFQDGEGASTFRAFNPTQA 240**

**241 EETYSMVTANRFWSQIFGIAFSNKRWLHFFMLFVPVTGLWMSAIGVVGLALNLRSYDFIS 300**

**301 QEIRAAEDPEFETFYTKNLLLNEGIRAWMAPQDQPHENFVFPEEVLPRGNAL 352**

**6dhe.1.D (template) 12 RGWFDILDDWLKRDRFVFVGWSGILLFPCAYLALGGWLTGTTFVTSWYTH 61**

**|||||:|||||||||||||||||:||||||||||||||||||||||||||**

**model_D2 12 RGWFDVLDDWLKRDRFVFVGWSGLLLFPCAYLALGGWLTGTTFVTSWYTH 61**

**6dhe.1.D (template) 62 GLASSYLEGCNFLTVAVSTPANSMGHSLLLLWGPEAQGDFTRWCQLGGLW 111**

**||||||||||||||||||:||::|||||||||||||||||||||||||||**

**model_D2 62 GLASSYLEGCNFLTVAVSSPADAMGHSLLLLWGPEAQGDFTRWCQLGGLW 111**

**6dhe.1.D (template) 112 TFIALHGAFGLIGFMLRQFEIARLVGVRPYNAIAFSAPIAVFVSVFLIYP 161**

**||:|||||||||||||||||||||||:|||||||||.||||||||||:||**

**model_D2 112 TFVALHGAFGLIGFMLRQFEIARLVGIRPYNAIAFSGPIAVFVSVFLMYP 161**

**6dhe.1.D (template) 162 LGQSSWFFAPSFGVAAIFRFLLFFQGFHNWTLNPFHMMGVAGVLGGALLC 211**

**|||||||||||||||||||||||.||||||||||||||||||:|||||||**

**model_D2 162 LGQSSWFFAPSFGVAAIFRFLLFLQGFHNWTLNPFHMMGVAGILGGALLC 211**

**6dhe.1.D (template) 212 AIHGATVENTLFQDGEGASTFRAFNPTQAEETYSMVTANRFWSQIFGIAF 261**

**||||||||||||:|||.::|||||.|||||||||||||||||||||||||**

**model_D2 212 AIHGATVENTLFEDGEQSNTFRAFEPTQAEETYSMVTANRFWSQIFGIAF 261**

**6dhe.1.D (template) 262 SNKRWLHFFMLFVPVTGLWMSAIGVVGLALNLRSYDFISQEIRAAEDPEF 311**

**|||||||||||||||||||||:||:||||||||:|||:|||:||||||||**

**model_D2 262 SNKRWLHFFMLFVPVTGLWMSSIGIVGLALNLRAYDFVSQELRAAEDPEF 311**

**6dhe.1.D (template) 312 ETFYTKNLLLNEGIRAWMAPQDQPHENFVFPEEVLPRGNAL 352**

**|||||||:|||||||||||||||||||||||||||||||||**

**model_D2 312 ETFYTKNILLNEGIRAWMAPQDQPHENFVFPEEVLPRGNAL 352**

**Notes S7.** Coding sequences (CDSs) of *psbA* (D1)

**>NC_028663.1:176059-177144 Cyanophage P-TIM40, PsbA based on complete genome**

**ATGACTACACTTTCACGTCGCAATAGTGGCGAATTGCTCCAAGGTTGGGAGCAGTTTTGCCAGTGGGTCACCAATACAAACAACAGAATTTACGTTGGTTGGTTTGGTGTGCTCATGATTCCATGTCTGCTCACTGCTGCAGCATGTTTTATCGTTGCATTTATTGCAGCACCACCTGTCGATATCGACGGTATTCGCGAACCAGTAGCAGGATCTCTACTCTATGGAAACAACATCATCTCAGGAGCAGTCGTCCCAAGCTCAAACGCAATCGGACTACACTTCTACCCAATCTGGGAAGCAGCAACAGTTGACGAATGGCTCTACAATGGAGGACCATACCAACTCGTTGTATTCCACTTCCTTATCGGTATCTCAGCTTACATGGGACGCCAATGGGAACTTAGTTACAGACTAGGCATGCGTCCTTGGATCTGTGTTGCATACTCTGCACCAGTATCTGCAGCGATGGCAGTTTTCCTCGTCTATCCATTCGGTCAAGGATCTTTCTCTGATGGTATGCCTCTTGGCATTTCAGGTACATTCAACTTTATGTTTGTATTCCAAGCAGAGCACAACATTCTAATGCACCCATTCCATATGCTTGGAGTAGCAGGTGTGTTTGGTGGATCTCTTTTCTCAGCAATGCACGGAAGTCTTGTTACTTCTTCACTTGTTAGAGAGACAACCGAAAACGAATCACAAAACTACGGTTACAAATTCGGACAAGAAGAAGAGACATACAACATTGTAGCTGCACACGGTTACTTTGGTCGTCTTATCTTCCAGTATGCTTCTTTCAATAACTCAAGAAGTCTTCACTTCTTCCTTGCAGCATTCCCAGTTGTTTGCATCTGGTTTACTGCAATGGGTGTTTGCACAATGGCATTCAACCTTAACGGTTTCAACTTCAACCAGTCTGTCTTAGACAACTCTGGTAAAGTGATTCCTACATGGGGAGATATCCTTAACCGTGCAAACCTTGGTATGGAAGTTATGCACGAGCGTAATGCTCACAACTTCCCACTTGACCTTGCTGCTGCAGAGACAACTCAGGTTGCTCTTACTGCACCATCAATCGGTTAA**

**>NC_006882.2:19242-20321 Prochlorococcus phage P-SSP7, PsbA based on complete genome**

**ATGGCTGCAATCTCAGTAACTAGAGAAGGCACAACTAACTGGCAGAAGTTTTGTGAGTGGGTCACAAGTACCGAGAACCGCCTTTATGTAGGTTGGTTTGGAGTACTTATGATTCCTTGCTTACTAGCTGCTACTACTTGCTTTATACTCGCCTTTATCGCAGCACCGCCTGTAGATATAGATGGCATACGTGAGCCAGTTTCCGGCTCGTTAATGTACGGAAACAATATTATTTCTGGAGCAGTAGTTCCAAGCTCCAATGCAATAGGACTGCACTTTTATCCGATCTGGGAAGCCGGCACTTTAGATGAGTGGTTATACAACGGCGGTCCATATCAACTTGTTGTCTTCCACTTCTTAATAGGAGTAGCAGCATACGCTGGTAGACAGTGGGAACTATCTTACAGATTAGGAATGAGACCATGGATATTCGTAGCATATACAGCACCACTATCAGCAGCTCTTGCTGTGTTTCTCGTTTACCCATTTGGGCAAGGGAGTTTTAGTGATGGTATGCCTCTTGGTATCTCTGGTACTTTTAACTTTATGTTCGTATTCCAAGCAGAACACAATATCCTTATGCATCCGTTCCACATGCTCGGTGTTGCTGGCGTATTCGGTGGATCTCTTTTCTCTGCTATGCACGGAAGTTTGGTTACTTCCTCGATCATCAAGGAAACAACTGAGGATGTATCGCAGAACTATGGCTATAAGTTCGGGCAAGATGAAGAGACATATAATATTGTCGCTGCACACGGGTACTTTGGGAGATTGATATTTCAATATGCTTCTTTCAATAATTCTCGTTCTTTACATTTCTTTCTTGCTACTTTCCCCGTGGTTGGCATATGGCTTACCTCCATGGGAATCTGCACTATGGCTTTCAACCTTAATGGTTTTAACTTTAACCAGTCAGTAGTTGATGTCAACGGAAAGATAATCCCAACATGGGCTGATGTATTAAATAGAGCTAACCTCGGATTTGAAGTTATGCACGAGCGCAATGCTCATAACTTCCCACTTGATTTAGCTTCGGCTGAGTCAACAAACATTGCACTAACAGCTCCAGAAATTGGTTGA**

**>NC_048026.1:19251-20327 Synechococcus T7-like virus S-TIP37, PsbA based on complete genome**

**ATGACTGCAATTTCTTATGGACAGCGGTCATCCTCTACGTGGGATGACTTCTGTCAGTGGGTGACGTCCACCAACAACCGTCTATACGTTGGCTGGTTTGGCATCTTGATGATTCCGTGCCTTCTTGCCGCAACCCTTTGTTTTATTACGGCGTTCGTCGCAGCGCCACCTGTCGACATCGATGGAATCCGTGAGCCTGTTGCAGGTTCTCTGATGTACGGCAACAACATTATTTCTGGAGCTGTTGTGCCTAGCAGCAATGCTATTGGCCTGCACTTCTATCCGATCTGGGAAGCCAACACTCTTGACGAGTGGCTCTACAACGGCGGCCCATATCAACTGGTTGTGTTCCACTTCCTCATTGGAGTCTTCTCCTACATGGGTCGTGAGTGGGAACTCAGCTATCGACTGGGTATGCGTCCCTGGATCTGCGTTGCTTACTCAGCTCCTGTAGCCGCTGCCTCTGCTGTCTTCTTGGTCTATCCATTTGGACAGGGTTCCTTCTCTGACGGTATGCCTCTTGGTATTTCTGGCACGTTTAACTACATGCTTGTGTTCCAAGCTGAGCACAACATTCTCATGCATCCTTTCCATATGCTGGGAGTTGCTGGTGTTTTTGGTGGTGCCCTGTTTAGTGCTATGCACGGCAGCTTGGTCACGTCTTCGCTTGTTCGCGAAACCACCGAAACTGAATCTCAAAACTATGGCTACAAGTTTGGGCAAGAGGAAGAGACTTACAACATTGTTGCCGCTCATGGTTATTTTGGTCGTCTTATCTTCCAGTACGCCAGCTTCAACAATAGTCGCAGTCTGCATTTCTTCCTGGCTGCTTGGCCTGTGGTTGGCATCTGGTTCGCTGCGCTAGGTGTTAGCACCATGGCGTTCAATCTGAACGGCTTCAACTTCAACCAATCCATCCAAGCTCAGGGTCACGTCGTGAACACCTGGGCCGACATTCTCAACCGTGCCAACCTCGGCTTCGAGGTGATGCACGAGCGAAATGCTCACAACTTCCCGCTTGATCTGGCTATGGCCTCTACCACTGAGGTCGCCCTTCAAGCACCTGCAATCGGCTAA**

**>NC_006883.2:211519-212601 Prochlorococcus phage P-SSM2, PsbA based on complete genome**

**ATGACAACTCTTCAAAAAAGGGAACAAGGCTTACTATCAGGTTGGTCTGAGTTCTGCGACTGGGTAACATCAACAAACAACAGAATCTATGTTGGTTGGTTTGGTGTTCTTATGATCCCATGTCTTCTAGCTGCAACAACATGCTTTATCGTAGCATTCATCGCAGCACCTCCCGTTGATATCGACGGAATCCGTGAACCAGTAGCTGGTTCATTCATGTATGGTAACAACATCATCTCTGGTGCAGTTGTTCCATCTTCAAACGCAATCGGTTTACACTTCTATCCTATCTGGGAAGCAGCAACTCTTGATGAGTGGCTCTACAATGGTGGTCCTTACCAGTTGGTAATCTTCCACTTCCTTATCGGAATCTCAGCATACATGGGAAGACAGTGGGAACTTTCATACCGTTTAGGTATGCGTCCTTGGATCTGTGTTGCTTATTCAGCACCTGTATCAGCAGCTTTCGCTGTATTCCTTGTTTACCCATTCGGTCAGGGTTCATTCTCAGACGGTATGCCTTTAGGTATATCAGGAACATTCAACTTCATGTTCGTATTCCAAGCAGAACATAACATCCTTATGCACCCATTCCATATGGCAGGTGTTGCAGGTATGTTCGGTGGAGCATTATTCTCTGCTATGCACGGTTCTCTTGTTACATCTTCACTTATCCGTGAAACCACTGGTTTAGATTCACAGAACTATGGTTACAAGTTCGGACAAGAAGAAGAGACATATAACATCGTTGCTGCACATGGATACTTCGGTAGACTTATCTTCCAGTATGCATCATTCAACAACAGCAGAAGTTTACACTTCTTCCTCGCATCATGGCCTGTTATCTGTGTATGGTTAACCTCTATGGGTATCTGCACAATGGCATTCAACCTAAATGGATTCAACTTCAACCAATCAGTTGTTGATGCATCAGGTAAGGTTGTTCCTACTTGGGGTGATGTTCTTAACAGAGCAAACCTTGGTATGGAAGTTATGCACGAAAGAAATGCACACAACTTCCCACTTGACTTAGCATCTGCTAACGAGACAGAAGTTGCTCTTGTTGCTCCTTCTATAGGTTAA**

**>NC_048015.1:168501-169589 Cyanophage S-TIM4, PsbA based on complete genome**

**ATGACAACTCTTTCAAGACAAGGCAGACAAGGCGGACTCCTACAAGGATGGCCTGAGTTCTGCGAATGGGTAACAAGCACTAACAACAGACTTTATGTTGGTTGGTTCGGTGTCTTAATGATCCCATGTTTATTAACAGCAGCAGCATGTTTCATTGTTGCATTTATTGCTGCACCACCTGTCGATATCGACGGAATCAGAGAACCTGTAGCAGGTGCTCTAATGTATGGAAACAACATCATCTCAGGTGCAGTTGTTCCATCTTCAAACGCAATCGGTCTACACTTCTACCCAATTTGGGAAGCAGCTACAATCGATGAGTGGTTATATAATGGTGGTCCTTACCAGTTGGTAATCTTCCACTTCCTAATCGGAATCTCTGCCTACATGGGTAGACAGTGGGAATTATCATACAGACTAGGTATGAGACCTTGGATCTGTGTTGCTTATTCAGCACCAGTATCTGCAGCATTTGCAGTATTCCTTGTATACCCATTTGGTCAGGGTTCTTTCTCTGATGGTATGCCTCTAGGAATTTCTGGAACATTCAACTTCATGTTCGTATTCCAAGCAGAGCACAACATTCTTATGCATCCCTTCCATATGGCAGGAGTAGCAGGTATGTTCGGTGGATCTCTATTCTCCGCAATGCATGGTTCACTTGTAACATCTTCTCTAATCAGAGAGACTACTGAGGAAGAATCTCAGAACTATGGTTACAAGTTCGGTCAAGAAGAAGAAACCTATAATATTGTTGCAGCACATGGATACTTCGGTAGATTAATCTTCCAGTATGCAAGTTTCAACAACTCAAGAAGTCTTCACTTCTTCCTAGCAGTGTTCCCAGTTGTATGTGTATGGTTAACCTCTATGGGTATCTGTACAATGGCATTCAACTTGAACGGATTTAACTTTAACCAATCAGTTGTAGATGTTAACGGTAAGATCATTCCTACATGGGGTGACGTACTTAACAGAGCAAACCTAGGTATGGAAGTAATGCATGAGCGTAACGCTCACAACTTCCCACTAGACTTAGCATCTGCTGAGACTACAGAGGTTGCTTTAACTGCACCAACAATCGGTTAA**

**>NC_015290.1:169639-170721 Prochlorococcus phage P-SSM7, PsbA based on complete genome**

**ATGACAACCCTACAAAAAAGAGAAACAGGTCTCCTCGCTGGATGGCCTCAGTTTACTGATTGGGTAACAAGCACAAACAACCGCATCTATGTTGGTTGGTTCGGTGTCTTGATGATCCCATGTCTATTAGCAGCAGCAACCTGCTTTATCGTTGCTTTCATCGCAGCTCCACCTGTGGACATCGATGGAATCAGAGAACCTGTAGCAGGTTCATTCATGTATGGTAACAACATCATCTCTGGTGCAGTTGTTCCATCTTCAAACGCAATCGGTCTCCACTTCTATCCTATTTGGGAAGCAGCAACTCTAGATGAGTGGTTGTATAACGGTGGTCCTTATCAATTAGTTATCTTCCACTTCCTTATCGGAATCTCTGCCTACATGGGTAGACAGTGGGAACTATCATACCGTTTAGGTATGCGTCCTTGGATCTGTGTAGCATATTCTGCTCCAGTGTCAGCAGCATTCGCTGTATTCTTAGTGTATCCTTTCGGTCAGGGATCTTTCTCAGACGGAATGCCTCTAGGTATCTCAGGTACATTTAACTTCATGTTCGTATTCCAAGCAGAGCACAACATTCTTATGCACCCATTCCATATGGCAGGGGTTGCTGGTATGTTCGGAGGAGCACTCTTCTCAGCAATGCACGGTTCACTTGTAACATCTTCTCTAATCAGAGAGACAACAGATAACGAGTCACAGAACTATGGATACAAATTCGGACAAGAAGAAGAAACATACAACATTGTTGCAGCACATGGATACTTTGGTAGACTTATCTTCCAATATGCTAGCTTTAACAATAGTCGTAGTCTTCACTTCTTCCTTGCTACATTCCCTGTAGTATGCATCTGGTTAACTTCAATGGGTATTTGCACAATGGCATTTAACCTTAATGGATTTAACTTCAACCAGTCAATCGTAGATGGATCTGGTAAAGTAGTTCCTACATGGGCAGACGTTCTTAACAGAGCAAACCTAGGTATGGAAGTAATGCATGAGCGTAATGCACACAACTTCCCACTTGACTTGGCAACTGCTGAGACATCTGAAGTTGCATTAATTGCTCCTTCTGTTGGTTAA**

**>NC_015288.1:181746-182825 Prochlorococcus phage Syn1, PsbA based on complete genome**

**ATGGCTTCAACTCTTTCTAGACAACAATCAACCTCTTCGTGGGAACAATTCTGCGAGTGGGTTACATCTACCAACAATCGTCTCTATGTCGGTTGGTTTGGTGTGCTGATGATCCCAACTCTGTTGGCGGCAACCATCTGTTTCATCGTTGCTTTCGTAGCAGCACCTCCTGTCGATATCGACGGTATCCGCGAACCCGTAGCTGGTTCACTCATGTATGGTAACAACATCATCTCTGGTGCTGTTGTCCCATCCTCCAACGCAATTGGTCTTCACTTCTACCCAATCTGGGAAGCTGCCTCACTTGATGAGTGGCTGTATAACGGTGGTCCTTTCCAACTGGTTGTTTTCCACTTCCTGATCGGCATCTACGCCTACATGGGACGTGAGTGGGAACTTTCTTACCGCTTAGGTATGCGTCCCTGGATCTGTGTAGCATACTCTGCTCCAGTCGCTGCAGCATCTGCTGTATTCTTAGTCTATCCTTTCGGTCAAGGTTCGTTCTCCGATGCAATGCCCCTCGGAATCTCAGGAACATTCAACTATATGTTGGTTTTCCAAGCTGAGCACAACATTCTTATGCATCCCTTCCACATGTTGGGTGTAGCAGGTGTATTCGGTGGTTCACTGTTTAGTGCAATGCACGGTTCTTTGGTTACATCTTCACTCGTCCGTGAGACGACTGAGCAAGAGTCACAGAACTATGGTTACAAGTTCGGTCAAGAAGAAGAGACCTATAACATCGTTGCAGCCCATGGCTACTTCGGTCGTTTGATCTTCCAATACGCATCATTCAACAACTCCCGTTCCTTGCACTTCTTCCTTGCTGCATGGCCTGTTGTTGGAATCTGGTTCACCGCACTTGGTGTCTCCACGATGGCATTCAACCTCAACGGTTTCAACTTCAACCAGTCCATCCTTGATGGTCAGGGTCGTGTCCTTAACACTTGGGCAGATGTATTGAACCGTGCTGGTCTTGGTATGGAAGTTATGCATGAGCGTAACGCACACAACTTCCCACTAGATCTTGCTGCTGCTGAGTCAACTCCTGTTGCACTTGTAGCACCTTCTGTTGGTTGA**

**>NC_015286.1:167040-168119 Synechococcus phage Syn19, PsbA based on complete genome**

**ATGGCTAGTTCAACTCTGTCACGCTCTCGCGTGTCTAACTGGGAATCCTTTTGCGATTGGGTTACCAGCACTAACAACCGTCTGTATGTCGGTTGGTTCGGCGTTCTGATGATCCCTACGTTGCTTGCAGCAACCATCTGTTTCATCATCGCCTTCGTCGGTGCTCCTCCTGTGGACATCGATGGTATCCGCGAACCAGTTGCTGGTTCACTCATGTATGGTAACAACATCATCTCTGGTGCAGTTGTTCCTTCTTCTAATGCTATTGGTCTTCACTTCTATCCCATCTGGGAAGCAGCAACCCTCGACGAGTGGTTGTACAATGGTGGTCCTTTCCAACTCGTAGTTTTCCACTTCCTGATTGGCATCTATGCCTATATGGGTCGTGAGTGGGAACTCTCTTACCGTCTCGGTATGCGTCCCTGGATCTGTGTTGCATACTCTGCTCCAGTCGCTGCAGCATCTGCTGTATTCCTCGTCTATCCTTTCGGTCAAGGTTCTTTCTCTGACGCGATGCCTCTTGGTATCTCTGGTACATTCAACTACATGCTTGTCTTCCAAGCAGAGCACAACATCCTGATGCACCCCTTCCATATGCTCGGCGTTGCTGGCGTATTCGGCGGTTCTCTGTTCAGTGCAATGCACGGTTCTCTGGTTACCTCTTCGCTGGTTCGTGAAACCACCGAAACTGAGTCCCAGAACTATGGTTACAAGTTCGGTCAGGAAGAAGAGACGTATAACATCGTCGCTGCTCATGGTTACTTCGGTCGCCTGATCTTCCAATACGCATCCTTCAACAACTCCCGCTCGCTGCACTTCTTCTTGGCAGCATGGCCTGTTGTTGGTATCTGGTTCACTGCACTTGGTGTTAGCACCATGGCATTCAACCTGAACGGTTTCAACTTCAACCAGTCTATCCTTGATAGTTCTGGTAAGGTTCTGCCTACTTGGGCAGACGTGCTCAACCGTGCTGGTCTGGGTATGGAAGTTATGCACGAGCGTAATGCTCACAACTTCCCTCTCGACCTTGCTGCTGCTGAGTCCACTCCTGTGGCACTCACCGCACCTGCTATCGGTTGA**

**>NC_015285.1:162337-163416 Prochlorococcus phage Syn33, PsbA based on complete genome**

**ATGGCTACAACTCTTTCAAGACAACAATCAACCTCGCCGTGGAATGATTTCTGCGAGTGGGTAACTTCTACCAATAACCGCCTCTATGTCGGTTGGTTCGGCGTACTGATGATTCCAACTCTGTTGGCAGCAACCATCTGTTTCATCGTCGCCTTCGTCGCTGCTCCCCCTGTGGACATCGACGGCATCCGTGAACCCGTCGCTGGTTCACTCATGTATGGTAACAACATCATCTCTGGTGCAGTTGTTCCATCTTCCAACGCAATTGGTCTTCACTTCTATCCCATCTGGGAAGCCGCATCACTTGATGAGTGGCTGTATAACGGTGGTCCTTTCCAACTCGTAGTATTCCACTTCCTGATCGGCATCTATGCCTACATGGGACGTGAATGGGAACTCTCTTACCGTCTTGGTATGCGTCCATGGATCTGCGTAGCATACTCTGCACCTGTTGCAGCAGCATCGGCAGTCTTCCTGGTCTATCCTTTCGGTCAAGGTTCTTTCTCTGACGCAATGCCCTTGGGTATCAGTGGTACATTCAACTACATGCTTGTCTTCCAAGCAGAGCACAACATTCTGATGCATCCCTTCCACATGTTGGGTGTCGCAGGTGTCTTCGGTGGTTCACTGTTCAGTGCAATGCACGGTTCTTTGGTTACATCTTCACTCGTCCGTGAGACGACTGAAACTGAGTCACAGAACTATGGTTACAAGTTCGGTCAAGAAGAAGAGACATACAACATCGTCGCAGCCCATGGTTACTTCGGTCGTTTGATCTTCCAATATGCATCATTCAACAACTCCCGTTCCTTGCACTTCTTCCTTGCTGCATGGCCTGTTGTCGGCATCTGGTTCACCGCCCTTGGCGTGTCAACCATGGCATTCAACCTGAACGGTTTCAACTTCAACCAGTCCATCCTTGATGGTCAGGGTCGTGTGTTGAACACCTGGGCAGACGTTCTGAACCGTGCAGGTTTGGGTATGGAAGTTATGCACGAGCGCAATGCTCACAACTTCCCACTTGACCTGGCAGCTGCCGAGTCAACTCCTGTTGCTCTTACCGCACCTACTGTCGGTTGA**

**>NC_015282.1:163737-164816 Synechococcus phage S-SM1, PsbA based on complete genome**

**ATGGCTACTACTCTCTCGCGCCAGTCTGGCGTGTCCTCCTGGGAATCATTCTGCGAATGGGTGACTTCCACAAACAACCGCCTCTATGTTGGTTGGTTCGGCGTTCTGATGATCCCTACGCTGCTTGCAGCAACCATCTGTTTCATCGTCGCCTTCGTCGCTGCTCCCCCTGTGGACATCGATGGTATTCGTGAACCTGTTGCAGGTTCGCTCATGTATGGTAACAACATCATCTCTGGTGCAGTTGTTCCCTCGTCCAATGCAATTGGTCTTCACTTCTACCCCATTTGGGAAGCCGCATCCCTTGATGAATGGCTTTACAACGGTGGTCCTTTCCAACTTGTCGTCTTCCACTTCCTGATCGGTATCTATGCATACATGGGTCGTGAGTGGGAACTGTCTTACCGTCTTGGTATGCGTCCTTGGATTTGTGTTGCTTACTCTGCACCCGTTGCAGCAGCATCCGCAGTCTTCCTGGTCTATCCTTTCGGTCAAGGTTCGTTCTCTGACGCAATGCCCCTGGGCATCTCTGGCACCTTCAACTACATGTTGGTGTTCCAAGCAGAGCACAACATCCTGATGCACCCCTTCCATATGCTCGGCGTTGCTGGCGTATTTGGCGGTTCTCTGTTCAGTGCAATGCACGGTTCTCTGGTTACTTCTTCGCTGGTTCGTGAAACCACCGAAACTGAGTCCCAGAACTATGGTTACAAGTTTGGTCAAGAAGAAGAAACATACAACATTGTTGCCGCTCACGGTTACTTTGGTCGTCTGATCTTCCAATACGCTTCATTCAACAACTCCCGTTCCTTGCACTTCTTCCTTGCTGCATGGCCTGTTGTTGGCATCTGGTTCACCGCACTTGGTGTTTCCACGATGGCATTCAACCTGAACGGTTTCAACTTCAACCAGTCCATCATCGATGGTCAGGGTCGTGTGCTCAACACTTGGGCAGACGTTCTCAACCGCGCTGGTCTGGGTATGGAAGTTATGCACGAGCGTAACGCTCACAACTTCCCTCTGGATCTCGCTGCTGCTGAGTCCACTCCTGTCGCTCTGACTGCACCCGCTATCGGTTGA**

**>NC_015281.1:167818-168894 Synechococcus phage S-ShM2, PsbA based on complete genome**

**ATGGCTACAACTCTTTCAAGACAACAAACCTCTCCGTGGAATGATTTCTGCGAGTGGGTAACATCAACTAACAACCGCCTCTATGTCGGTTGGTTCGGCGTACTGATGATTCCAACTCTGTTGGCAGCAACTATCTGTTTCATCGTCGCCTTCGTCGCTGCTCCCCCTGTGGACATCGACGGCATCCGTGAACCCGTCGCTGGTTCACTCATGTATGGTAACAACATCATCTCTGGTGCAGTTGTCCCTTCTTCTAATGCTATTGGACTTCACTTCTATCCCATCTGGGAAGCAGCATCACTCGATGAGTGGTTGTATAACGGTGGTCCTTTCCAACTAGTAGTCTTTCACTTCCTCATCGGTATCTATGCATACATGGGACGTGAATGGGAACTTTCATATCGTTTAGGTATGCGTCCATGGATCTGTGTTGCATACTCTGCACCAGTCGCTGCAGCGAGTGCAGTATTCCTAGTGTATCCTTTCGGTCAAGGTAGTTTCTCTGATGCAATGCCACTCGGAATCTCAGGAACATTCAACTACATGTTGGTCTTCCAAGCTGAGCACAATATTCTCATGCATCCCTTCCATATGCTCGGCGTTGCTGGGGTATTTGGTGGCAGTTTGTTTAGTGCTATGCACGGAAGTTTGGTTACTTCTTCTCTCGTTAGAGAGACGACTGAAACCGAATCCCAAAACTACGGTTACAAGTTCGGACAAGAAGAAGAAACATACAACATCGTAGCCGCTCATGGTTACTTCGGTCGTTTGATCTTCCAATACGCTTCATTCAACAACTCCCGCTCCTTGCACTTCTTCCTTGCTGCATGGCCTGTTGTCGGCATCTGGTTCACCGCCCTTGGCGTCTCCACGATGGCATTCAACCTGAACGGTTTCAACTTCAACCAGTCCATCCTTGATGGTCAGGGTCGTGTGTTGAACACCTGGGCAGATGTTCTGAACCGCGCTGGTCTGGGTATGGAAGTTATGCATGAGCGTAACGCACACAACTTCCCACTCGATCTTGCTGCTGCTGAGTCCACACCTGTGGCCTTGATTGCTCCTTCTGTTGGTTGA**

**>NC_016658.1:c6219-5146 Cyanophage NATL1A-7, PsbA based on complete genome**

**ATGACAACAGCCACATTATCACCTCTTTCCAATTGGGATAAGTTCTGTGACTGGGTTACTAGCACCAACAACCGCCTCTATGTGGGGTGGTTTGGTGTCTTAATGATACCCGCACTCTTAACCGCAACTACTGCATTTATATTAGCATTCGTAGCTGCACCCCCAGTCGACATCGACGGCATACGTGAACCCGTCGCAGGATCTCTACTCTATGGAAACAACATCATATCAGGAGCCGTTGTACCCAGCTCCAACGCAATTGGGTTACACTTCTATCCCATCTGGGAAGCAGCCAATCTCGATGAATGGCTTTACAATGGAGGACCATACCAACTCGTCGTCTTCCACTTCCTTATTGGTATCGCAGCTTACTTGGGACGCCAATGGGAACTTAGTTACAGATTAGGAATGCGACCATGGATTTGCGTAGCATATTCAGCACCGGTTGCCGCAGCCTACTCAGTATTTTTAGTATACCCTTTCGGACAGGGAAGTTTCAGTGATGGTATGCCTCTTGGTATCTCTGGTACTTTTAACTTTATGTTCGTATTCCAGGCAGAACACAATATCCTTATGCATCCGTTCCACATGCTCGGTGTTGCTGGGGTATTCGGTGGAGCTTTGTTCGCTGCTATGCATGGAAGTCTTGTTACTTCTTCGCTCATTCGTGAAACAACTGGGCTTACTTCTCAGAACTATGGATATAAATTCGGCCAAGAGGAAGAAACGTATAACATTGTTGCGGCTCATGGCTACTTTGGGAGACTCATCTTCCAGTATGCTAGCTTTAACAATAGCCGTAGTTTACATTTCTTCCTGGCTACTTGGCCCGTCGTTTGCATATGGCTTACCTCTATGGGAATCTCCACTATGGCTTTTAATCTTAACGGCTTTAACTTCAATCAGTCAGTCGTTGACTCTAGCGGTAGAATCGTTCCCACTTGGGCAGATGTCCTGAACCGTGCCAACCTTGGTATGGAAGTAATGCACGAACGAAATGCTCACAACTTCCCACTTGATCTAGCAGCTGCTGAGTCAAGTGAAGTAGCTTTAACAGCACCCTCAATTGGTTAG**

**>NC_019516.1:8197-9279 Cyanophage S-TIM5, PsbA based on complete genome**

**ATGTCTGCTTCAACTCTCTCACGTCAACAATCCCAAAGCACTTGGGAAAATTTTTGCGAATGGGTCACTTCGACCAATAATCGCTTGTATGTCGGCTGGTTTGGCGTTCTCATGATCCCAACCCTTCTTGCCGCTACAATTTGCTTCATTACCGCATTCGTTGCTGCTCCTCCTGTGGACATCGACGGCATCCGCGAACCAGTTGCTGGATCTCTCATGTACGGTAACAACATCATCTCTGGTGCAGTTGTTCCCAGCTCTAACGCAATTGGCCTCCACTTCTACCCCATCTGGGAAGCCGCTTCTCTTGATGAGTGGCTCTACAACGGTGGTCCTTACCAGCTTGTAGTTTTCCACTTCCTCCTTGGCGTCTTTGCTTATATGGGTCGTGAATGGGAACTTTCTTACCGCCTTGGCATGCGTCCTTGGATCTGTGTTGCATACTCTGCTCCTGTTGCCGCTGCTTCTGCTGTATTCCTTGTCTATCCTTTTGGTCAAGGTTCGTTCTCTGATGGTATGCCTCTTGGTATCTCCGGTACGTTCAACTATATGCTTGTCTTCCAGGCTGAGCACAACATCCTGATGCACCCCTTCCACATGCTTGGTGTTGCTGGTGTCTTCGGCGGATCTCTCTTTTCCGCAATGCATGGTTCACTCGTTACTTCTTCGCTGGTTCGTGAGACCACCGAAACCGAGTCTCAAAACTATGGCTACAAGTTCGGTCAAGAAGAGGAAACTTACAACATTGTTGCCGCTCATGGCTACTTTGGTCGCTTGATCTTCCAATATGCATCTTTTAACAATTCTAGAAGCTTGCATTTCTTCCTCGCTGCGTGGCCTGTGGTGGGAATCTGGTTTACCGCCCTCGGCGTCTCGACCATGGCTTTCAACCTCAATGGCTTCAACTTCAACCAGTCCATTATCGACGGACAAGGACGAGTGCTCAACACCTGGGCAGACGTACTTAACCGCGCAGGACTCGGTATGGAAGTCATGCACGAAAGAAACGCACACAACTTCCCGCTTGACCTTGCGGCTGCTGAGTCCACACCTGTGGCCTTGACTGCTCCTTCTATCGGCTGA**

**>NC_047734.1:186641-187720 Cyanophage S-RIM44 isolate Np_42_0711, PsbA based on complete genome**

**ATGGCTTCAACTCTTTCTAGACAACAATCAACCTCTTCGTGGGAACAATTCTGCGAGTGGGTTACATCTACCAACAATCGTCTCTATGTTGGTTGGTTTGGTGTGCTGATGATCCCAACTCTGTTGGCGGCAACCATCTGTTTCATCGTTGCTTTCGTAGCAGCACCCCCCGTCGATATCGATGGTATCCGTGAACCCGTAGCTGGTTCACTCATGTATGGTAACAACATCATCTCTGGTGCTGTTGTCCCATCCTCCAACGCAATTGGTCTTCACTTCTACCCCATCTGGGAAGCTGCCTCACTCGATGAGTGGCTCTACAACGGCGGTCCTTTCCAACTGGTTGTCTTCCACTTCCTGATCGGCATCTACGCCTATATGGGACGTGAGTGGGAACTTTCTTACCGCTTAGGTATGCGTCCCTGGATCTGTGTAGCATACTCTGCTCCAGTAGCAGCAGCATCTGCTGTATTCCTCGTCTATCCTTTCGGTCAAGGTTCATTCTCTGATGCGATGCCTTTGGGCATCTCTGGAACCTTCAACTACATGTTGGTCTTCCAAGCAGAGCACAACATCCTGATGCACCCCTTCCACATGCTGGGAGTTGCTGGTGTCTTCGGTGGTTCATTGTTCTCAGCGATGCACGGTTCTTTGGTTACATCTTCACTCGTCCGTGAGACGACTGAAACTGAGTCACAGAACTATGGTTACAAGTTCGGACAAGAAGAAGAAACATACAACATTGTAGCCGCTCATGGTTACTTTGGTCGCTTGATCTTCCAATACGCTTCATTCAATAACTCCCGTTCACTCCACTTCTTCCTCGCCGCATGGCCTGTAGTGGGTATCTGGTTCACTGCACTTGGTGTTAGCACCATGGCATTCAACCTGAACGGTTTCAACTTCAACCAGTCCATCCTTGATGGTCAGGGTCGTGTGTTGAACACCTGGGCAGACGTGCTTAACCGCGCTGGTCTTGGTATGGAAGTCATGCATGAGCGTAATGCTCATAACTTCCCACTTGACCTTGCTGCTGCTGAGTCCACTCCTGTGGCACTTGTAGCACCTTCTGTTGGTTGA**

**>NC_047733.1:160833-161912 Synechococcus phage S-RIM8 isolate RW_22_0300, PsbA based on complete genome**

**ATGACTGCAACACTTTCGCAACAACGTTCTACTAACACCTGGGAACAGTTTTGTAACTGGGTCACCAGCACCGACAACCGCCTTTATGTTGGTTGGTTCGGCGTCCTGATGATTCCCTGCTTGCTCGCTGCTACCATTTGTTTCATCATTGCCTTCGTTGGTGCTCCCCCTGTGGACATCGACGGCATTCGTGAACCCGTCGCTGGTTCGCTCATGTATGGAAACAACATCATCTCTGGTGCTGTTATCCCTTCTTCTAACGCTATCGGTCTGCACTTCTACCCCATCTGGGAAGCAGCAAGTCTGGACGAATGGCTCTACAACGGTGGTCCTTTCCAACTCGTTGTGTTCCACTTCCTGATTGGCATCTATGCTTACATGGGTCGTGAGTGGGAACTTTCTTACCGTCTGGGAATGCGTCCTTGGATTTGCGTAGCATACTCTGCTCCTGTCGCTGCAGCGAGTGCAGTGTTCCTGGTCTATCCTTTCGGTCAAGGTTCTTTCTCTGATGCAATGCCTCTGGGCATCTCTGGTACGTTCAACTACATGCTTGTCTTCCAAGCAGAGCACAACATTCTCATGCACCCCTTCCATATGCTCGGCGTAGCAGGTGTGTTTGGCGGTTCTCTGTTCAGTGCAATGCACGGTTCGCTGGTTACCTCTTCGCTGGTTCGTGAAACCACCGAAAACGAGTCCCAGAACTATGGTTACAAGTTCGGTCAAGAAGAAGAGACCTACAACATCGTTGCTGCCCACGGGTACTTCGGTCGTCTGATTTTCCAATACGCTTCCTTCAACAACTCCCGCTCGCTGCACTTCTTCCTTGCTGCTTGGCCTGTTGTTGGCATCTGGTTCACTGCACTGGGCGTAAGCACCATGGCATTCAACCTGAATGGTTTCAACTTCAACCAGTCTATCGTTGATAGTCAGGGTAAAGTTATCAACACCTGGGCAGACGTTCTCAACCGTGCTGGTCTTGGCATGGAAGTGATGCACGAGCGCAATGCTCACAACTTCCCTCTTGACCTTGCTGCTGCTGAGTCCACTCCTGTGGCACTCCAAGCACCTGCAATCGGTTGA**

**>NC_031935.1:177658-178629 Synechococcus phage S-WAM2 isolate 0810PA29, PsbA based on complete genome**

**ATGATCCCTACGCTGCTCGCTGCTGCTATCTGTTTCATCGTCGCTTTCGTCGCTGCTCCTCCTGTGGACATCGACGGCATCCGTGAACCCGTCGCTGGTTCCCTGATGTATGGCAACAACATCATTTCTGGTGCTGTTGTTCCTTCTTCTAACGCTATTGGTCTCCACTTCTATCCCATCTGGGAAGCAGCTTCTCTCGATGAGTGGCTCTACAATGGTGGTCCTTTCCAACTCGTTATCTTCCACTTCCTGATCGGTATCTATGCATACATGGGTCGTGAGTGGGAACTTTCTTACCGCCTAGGTATGCGTCCCTGGATCTGTGTAGCATACTCTGCTCCAGTCGCCGCAGCATCTGCTGTATTCCTTGTTTATCCTTTCGGTCAAGGTTCTTTCTCTGACGCTATGCCCCTGGGTATCAGTGGCACCTTCAACTACATGCTTGTCTTCCAAGCAGAGCACAACATCCTGATGCACCCCTTCCACATGTTGGGCGTCGCTGGTGTCTTTGGTGGTTCTCTGTTCAGTGCGATGCACGGTTCTCTGGTTACTTCCTCGCTGGTTCGTGAAACCACCGAGAACGAAAGTCAGAACTATGGTTACAAGTTCGGTCAAGAAGAAGAGACCTACAACATCGTTGCTGCTCATGGTTACTTCGGTCGCCTGATCTTCCAATACGCATCCTTCAACAACTCCCGTTCGCTGCACTTCTTCCTCGCAGCATGGCCTGTTGTCGGTATCTGGTTCACTGCTCTTGGTGTTAGCACCATGGCATTCAACCTCAACGGTTTCAACTTCAACCAGTCCATCATGGATAGTCAGGGCAAAGTCCTGAACACCTGGGCAGACGTTCTCAACCGTGCTGGTCTTGGTATGGAAGTCATGCACGAGCGTAATGCTCACAACTTCCCTCTTGACCTCGCTGCTGCTGAAAGCACTCCTGTTGCTCTCACCGCACCTGCTGTTGGTTGA**

**>NC_031922.1:160518-161603 Synechococcus phage S-CAM9 isolate 1109NB16, PsbA based on complete genome**

**ATGGCTAACTCTACACTTTCACAAGGTTACAATCAACAATCCACTTGGGAACAATTCTGCGAGTGGGTAACTTCTACAAACAATCGTCTTTACGTCGGTTGGTTCGGTGTGCTGATGATCCCAACTCTGTTGGCAGCAACCGTCTGCTTTATTACTGCGTTCGTTGCTGCTCCCCCCGTGGACATCGACGGTATCCGTGAACCCGTTGCTGGTTCACTGATGTATGGAAACAACATCATCTCTGGTGCAGTTGTTCCTTCTTCCAACGCAATTGGTCTTCACTTCTACCCCATTTGGGAAGCTGCATCTCTTGATGAATGGCTCTACAATGGTGGTCCTTACCAACTGGTCGTATTTCACTTCCTGATCGGCATCTTCTGCTACATGGGTCGCGAGTGGGAACTTTCCTACCGCCTCGGCATGCGCCCCTGGATCTGCGTTGCATATTCTGCACCTGTTGCTGCTGCGAGTGCAGTATTCCTCGTCTATCCTTTCGGTCAAGGTTCTTTCTCTGATGGTATGCCCCTGGGTATCTCTGGTACCTTCAACTTCATGCTTGTCTTCCAGGCAGAACACAACATCCTGATGCACCCCTTCCACATGTTGGGTGTTGCTGGTGTCTTCGGTGGTTCTCTGTTCTCCGCAATGCATGGTTCACTGGTTACTTCTTCCCTGGTTCGTGAAACCACTGAGAACGAGTCCCAGAACTATGGTTACAAGTTTGGTCAAGAAGAAGAGACCTACAACATCGTCGCTGCTCATGGTTACTTCGGTCGCCTGATCTTCCAATACGCTTCCTTCAACAACTCCCGTTCGTTGCACTTCTTCCTCGCAGCATGGCCTGTTGTCGGTATCTGGTTCACTGCACTTGGTGTCTCCACCATGGCATTCAACCTGAACGGATTCAACTTCAACCAGTCCATCATGGATGGTCAGGGCAAAGTCCTGAACACCTGGGCTGACGTTCTGAATCGTGCTAACCTTGGTATGGAAGTAATGCACGAGCGTAATGCTCACAACTTCCCTCTGGATCTCGCTGCTGCTGAGTCCACTCCTGTTGCACTGACCGCACCTACTGTTGGTTGA**

**>NC_031906.1:188806-189882 Synechococcus phage S-CAM3 isolate 1010CC42, PsbA based on complete genome**

**ATGACTGCAACTCTCTCACGTCAGCAGAGTGGCACTTGGGAATCATTCTGCGAATGGGTCACCTCTACTAACAACCGTCTGTATGTCGGTTGGTTCGGTGTCCTGATGATCCCTACGCTTCTCGCTGCAACCATCTGCTTCATCGTCGCCTTTGTCGCTGCACCCCCTGTAGACATTGATGGCATCCGCGAACCCGTCGCTGGTTCACTCATGTATGGAAACAACATCATCTCTGGTGCTGTTGTCCCTTCATCCAACGCAATTGGTCTTCACTTCTACCCCATCTGGGAAGCCGCCTCACTCGATGAGTGGCTCTACAACGGTGGTCCTTTCCAACTGGTAGTCTTCCACTTCCTCATCGGCATCTACGCCTACATGGGTCGTGAGTGGGAACTCTCTTACCGTCTCGGTATGCGTCCTTGGATCTGCATTGCTTACTCTGCACCTGTTGCAGCAGCATCCGCAGTCTTTCTGGTCTATCCTTTTGGTCAAGGTTCTTTCTCTGACGCAATGCCCTTGGGTATCAGTGGAACCTTCAACTACATGCTTGTCTTCCAAGCAGAGCACAACATCCTGATGCACCCCTTCCACATGCTGGGAGTCGCAGGTGTCTTCGGTGGTTCACTGTTCTCCGCAATGCATGGTTCACTCGTTACCTCTTCACTGGTTCGTGAAACCACTGAAACCGAGTCCCAGAACTATGGTTACAAGTTTGGACAAGAAGAAGAAACGTACAACATCGTTGCTGCTCATGGATATTTCGGTCGTCTTATCTTCCAATATGCATCGTTCAACAACTCCCGTTCACTTCACTTCTTCCTCGCTGCCTGGCCAGTCGTGGGTATCTGGTTTACCGCCCTCGGCGTCAGCACCATGGCATTCAACCTCAATGGGTTCAACTTCAATCAATCCATTCTTGATGGTCAAGGTCGTGTTCTGAACACATGGGCAGACGTTCTCAACCGTGCTGGTCTTGGTATGGAAGTCATGCATGAGCGTAACGCTCACAACTTCCCTCTTGATCTGGCAGCAGCAGAGTCCACTCCTGTGGCACTCACCGCTCCCGCAATTGGTTGA**

**>NC_031242.1:163880-164959 Cyanophage S-RIM50 isolate RW_29_0704, PsbA based on complete genome**

**ATGACTGCTACACTTTCACAACAACGTTCTACTAACACCTGGGAACAATTCTGTAACTGGGTAACTTCAACCGACAATCGTTTGTATGTCGGTTGGTTCGGCGTCCTGATGATTCCCTGCCTGCTGGCAGCGACTACATGTTTCATCATCGCCTTCATCGGTGCTCCTCCTGTGGACATCGATGGCATCCGCGAACCCGTCGCTGGTTCGCTCATGTATGGAAACAACATCATCTCTGGTGCTGTTATCCCTTCTTCTAACGCTATCGGTCTGCACTTCTACCCCATCTGGGAAGCAGCAAGTCTGGACGAATGGCTCTACAACGGTGGTCCTTTCCAACTCGTTGTGTTCCACTTCCTGATTGGCATCTATGCTTACATGGGTCGTGAGTGGGAACTTTCTTACCGTCTTGGTATGCGTCCTTGGATTTGCGTAGCATACTCTGCTCCTGTCGCTGCAGCGAGTGCAGTGTTCCTGGTCTATCCTTTCGGTCAAGGTTCTTTCTCTGATGCAATGCCTCTGGGCATCTCTGGAACCTTCAACTACATGTTGGTCTTCCAGGCTGAGCACAACATCCTCATGCACCCCTTCCATATGCTCGGCGTAGCAGGTGTGTTTGGCGGTTCTCTGTTCAGTGCAATGCACGGTTCGCTGGTTACTTCTTCGCTGGTTCGTGAGACCACCGAAAATGAGTCCCAGAACTATGGTTACAAGTTTGGTCAGGAAGAAGAAACCTACAACATCGTTGCCGCTCACGGTTACTTTGGTCGTCTGATTTTCCAATACGCTTCCTTCAACAACTCCCGTTCGCTGCACTTCTTCCTCGCAGCATGGCCTGTTGTCGGTATCTGGTTTACTGCTCTGGGCGTTAGCACCATGGCATTCAACCTCAACGGTTTCAACTTCAACCAGTCCATCATCGATGGTCAGGGTCGTGTGCTCAACACCTGGGCAGACGTTCTCAACCGTGCTGGTCTGGGCATGGAAGTGATGCACGAGCGTAATGCTCACAACTTCCCTCTCGACCTTGCTGCTGCTGAGAACACTCCTGTTGCTCTCAAGGCACCTGCTGTTGGTTGA**

**>ath:ArthCp002 K02703 photosystem II P680 reaction center D1 protein [EC:1.10.3.9] | (RefSeq) psbA; photosystem II protein D1 (N)**

**atgactgcaattttagagagacgcgaaagcgaaagcctatggggtcgcttctgtaactggataactagcactgaaaaccgtctttacattggatggtttggtgttttgatgatccctaccttattgaccgcaacttctgtttttattatcgcattcattgctgctcctccagtagatattgatggtattcgtgaacctgtttctggatctcttctttacggaaacaatattatttccggtgccattattcctacttctgcagctattggattgcatttttacccaatctgggaagctgcatccgttgatgaatggctatacaacggcggtccttatgaactaattgttctacactttttacttggtgtagcttgttatatgggtcgtgagtgggaacttagtttccgtctgggtatgcgtccttggattgctgttgcatattcagctcctgttgcagctgcgactgctgttttcttgatctatccaattggtcagggaagtttttctgatggtatgcctctaggaatctctggtactttcaactttatgattgtattccaggctgagcacaacattcttatgcacccatttcacatgttaggtgtagctggtgtattcggcggctccctttttagtgctatgcatggttccttggtaacttctagtttgatcagggaaaccacagaaaatgaatctgctaatgaaggttacagattcgggcaagaagaagaaacttacaacattgtagctgctcacggttattttggccgattgattttccaatatgctagtttcaacaattctcgttctttacatttcttcttagcggcttggccggtagtaggtatttggtttactgctttaggtattagtactatggctttcaacctaaatggtttcaatttcaaccaatcagtagttgatagtcaaggacgtgttattaatacttgggctgatattattaaccgtgctaaccttggtatggaagttatgcatgaacgtaatgctcacaacttccctctagacctagctgctgttgaggctccatctacaaatggataa**

**>S.oleracea:2715607 K02703 photosystem II P680 reaction center D1 protein [EC:1.10.3.9] | (RefSeq) psbA, SpolCp002; photosystem II protein D1 (N)**

**atgactgcaattttagagagacgcgaaagcgaaagcctatggggtcgcttctgtaattggataaccagcactgaaaaccgtctttacattggatggtttggtgttttgatgatccctaccttattgactgcaacttctgtatttattatagccttcattgctgctcctccagtagatattgatggtattcgtgaacctgtttccggatctctactttacggaaataatattatttcgggtgccattattcctacttctgcagctatagggttgcacttttatccaatctgggaagcggcatcggttgatgagtggttatacaatggtggtccttatgaactaattgttctacacttcttacttggtgtagcttgttatatgggtcgtgagtgggaacttagtttccgtctaggtatgcgcccttggattgctgttgcatattccgctccagttgcagcggctactgctgttttcttgatctacccaatcggtcaaggaagcttttctgatggtatgcctctaggaatctccggtactttcaactttatgattgtattccaggctgagcacaacatccttatgcacccatttcacatgttaggtgtagctggtgtattcggcggctccctatttagtgctatgcatggttccttggtaacttctagtttgatcagggaaaccacagaaaatgaatctgctaatgaaggttacagattcggtcaagaggaagaaacttataatatcgtagctgctcatggttattttggtcgattgatcttccaatatgctagtttcaacaactctcgttctttacacttcttcttagctgcttggcctgtagtaggtatttggtttactgctttaggtattagtactatggctttcaacttaaatggtttcaatttcaaccaatctgtagttgatagtcaaggtcgtgtaattaatacttgggctgatatcattaaccgtgctaaccttggtatggaagttatgcatgaacgtaatgctcataacttccctctagacctagctgctattgaagctccatctacaaatggataa**

**>T.elongatus:tlr1843 K02703 photosystem II P680 reaction center D1 protein [EC:1.10.3.9] | (RefSeq) psbA1; photosystem II D1 protein (N)**

**atgaccacaactctccaacgtcgcgaaagcgcgaatttgtgggagcggttttgtaactgggtgacgagcaccgataaccgcctttatgtgggctggtttggggtgatcatgatccccaccctattagccgcaaccatctgctttgtgattgccttcatcgctgctccccctgtggacatcgatggcatccgtgagcctgtttctggctctttgctctatggcaacaacatcatcacgggtgcagttgtcccctctagcaacgccattggcttgcacttctaccccatttgggaagctgcttccctcgatgagtggctctacaacggtggcccctaccaactgatcatcttccacttcctgttgggtgcctcctgctacatgggtcgccagtgggaactcagctaccgcctcggtatgcggccttggatctgcgtggcctactctgcccccctggcttctgcctttgcagtcttcttgatctaccccattggtcaaggcagcttctctgacgggatgcccctcggtatctctggtaccttcaactttatgattgtgttccaagcggagcacaacattctcatgcaccccttccaccaactgggtgtagccggtgtctttggtggggcgctgttctgcgccatgcacggttctctggtgacctccagcttgatccgtgaaaccaccgaaaccgaatccgccaactacggttacaaatttggtcaagaggaagaaacctacaacatcgtggctgcccacggttactttggccggttgatcttccaatacgccagcttcaacaacagccgctccctgcacttcttcttggccgcttggccggtggtgggtgtgtggtttaccgccttggggatcagcaccatggcctttaacctgaatggcttcaacttcaaccactcggtcattgatgccaagggcaacgtgatcaacacctgggcggacatcatcaaccgtgccaacttgggtatggaagtgatgcacgagcgcaatgctcacaacttccccctcgacttggccagcgctgagtctgctcctgtggccatgattgctcccagcatcaacggctaa**

**>Synechocystis PCC 6803:slr1181 K02703 photosystem II P680 reaction center D1 protein [EC:1.10.3.9] | (GenBank) psbA1; photosystem II D1 protein (N)**

**atgactactacccaattaggattacaggaacaaagcctgtggtcacggttctgttgttggattactagcacttctaatcgcctttacatcggctggtttggggtgttgatgatccccactttgttaaccgctaccacctgttttattatcgctttcatcgccgctcccccggtggatattgatggcattcgggagcccattgctggttctttactgtacggtaacaatatcatcactgccgccgtagtgcccagttccaatgccattggcttgcacttttaccccatttgggaagcccatagcctcgatgaatggctttacaatggtggcccttaccaattgattgtttttcactttttaattgggattttttgttatctcggtcgtcaatgggaattgtcttaccgtctaggaatgcgtccttggatctgtgttgcctatagcgcccccgtcgccgctgccactgctactttgttaatctattccattggtcaaggctctttctccgatggtttacccctaggcatcagtggtacttttaacttcatgttggtgctgcaagcggaacataacgtattgatgcatcctttccacatgttaggggtagctggggtgtttggtggggctttgtttgccgctatgcacggttccttggttacttcttccttaattcgagaaactacagaagttgaatcccaaaatcaaggttacaaatttggtcaagaggaagaaacctacaacatcgttgctgcccacggttactttggtcgcttaattttccaatatgcttcctttaacaatagtcgggctctccacttctttctgggagcttggccagtggtaggcatctggtttgccgctttggcagtttgttgtttcgctttcaacctcaacggttttaacttcaaccaatccattctggatgcccaaggccgtcccgtgagcacctgggccgatgtgattaaccgtgccaacattggttttgaggtgatgcacgaacgcaacgttcacaatttccccttggatttagcttccggtgatgcccaaatggtggccctgaacgcccccgccattgaaggctag**

**>S. elongatus PCC 7942:Synpcc7942_0424 K02703 photosystem II P680 reaction center D1 protein [EC:1.10.3.9] | (GenBank) photosystem q(b) protein (N)**

**atgaccagcattcttcgcgagcaacgccgcgataacgtttgggatcggttttgtgagtgggtaaccagcaccgacaaccgcatctacgtgggttggttcggcgtgctgatgatccccactctgctgaccgccaccatctgcttcatcgttgcgttcattgcagcccctcccgtcgacatcgacggcatccgtgagcccgttgccggctctctcatgtatggcaacaacatcatttccggcgctgttgttccttccagcaacgccatcggcctgcatttctatccgatttgggaagccgctagcctcgacgagtggctgtacaacggtggtccttaccaattagtggtcttccacttcttgctgggtatcagctgctacatgggtcgtcaatgggagctgtcgtaccgcctcggtatgcgcccttggatctgtgttgcatacagtgctccactctcggctgcttttgcagtgtttctgatctacccgatcggccaaggttcgttctcggacggcatgcccctgggtatcagcggcaccttcaacttcatgttcgtgttccaagcagagcacaacattttgatgcaccccttccacatgctgggtgtggctggtgtgttcggtggttcgctgttctcggcaatgcacggttcgttggtgaccagctcgctggtgcgtgagacgaccgagaccgagagccaaaactacggctacaaatttggtcaagaggaagagacctacaacatcgtggcagcccacggttacttcggtcgcttgatcttccaatacgcatcgttcaacaacagccgttcgctgcacttcttcctgggtgcatggccggtcgtgggcatctggtttacctccatgggcatcagcaccatggcgttcaacctgaatggtttcaacttcaaccagtcggttttggatagccaaggcaaagtgatcaacacttgggcagatgtgttgaaccgtgccaacttgggcatggaagtgatgcacgagcgtaatgctcacaacttcccgctcgacttggcagcaggcgaagcgaccccggtcgctttgactgcgccttcaattcacggttaa**

**>X00735.1:87-321,757-839,1283-1861,2296-2394,3012-3053 Euglena gracilis chloroplast psbA locus with 32-kd protein gene (herbicide binding protein)**

**ATGATTTCACCTGTTTTAAAGAAATATGCAAGACCTAGTCTTTGGTATCGTTTTTGTGCTTGGGTAGCTTCAAAGAAAAATCGTCTTTATGTAGGATGGTTCGGTGTTTTGATGATTCCAACTTTACTTACAGCTGCAACTGTATTTATTATTGCTTTCATTGCAGCTCCGCCTGTTGATATCGATGGTATTCGTGAGCCTGTGTCTGGATCTCTTTTTTATGGAAATAATATAATTACTGGTGCTGTAGTTCCAACTTCAAATGCTATTGGATTGCATTTTTATCCTATTTGGGAAGCAACTTCATTAGATGAATGGTTGTATAATGGTGGTCCTTATCAATTAATCGTATGCCATTTCTTTATCGGTATTTGTTCTTATATGGGAAGAGAATGGGAGCTTTCATTCCGATTAGGAATGCGCCCGTGGATTGCAGTTGCTTATTCTGCTCCTGTTGCTGCTGCAAGTGCTGTATTTATTGTTTATCCTTTAGGTCAAGGTTCTTTTTCAGATGGTATGCCTTTAGGTATTTCAGGTACTTTTAACTTTATGATTGTTTTCCAAGCTGAACATAATATTTTAATGCATCCATTTCATATGCTTGGTGTTGCTGGTGTTTTTGGTGGTTCTCTTTTCTCTGCTATGCATGGTTCTTTGGTAACATCTAGTTTGCTTCGTGAAACGACTGAAAATGAATCAATAAATGTTGGTTACAAGTTTGGTCAAGAAGAAGAAACATATAATATTATTGCTGCTCACGCTTATTTTGGTCGTTTAATCTTCCAATATGCGTCGTTCAATAATTCACGTTCATTGCATTTCTTTTTAGCTGTTTGGCCTGTTGTTGGTATTTGGTTTACAGCATTAGGTGTTTCAACTATGGCATTTAATTTAAACGGTTTTAATTTTAATCAATCTGTTATTGATTCACAAGGTCGCGTTATCAATACTTGGGCTGATATTATTAATAGAGCAAATTTAGGTATGGAAGTTATGCACGAACGTAATGCACATAATTTCCCATTAGATTTGGCTTAA**

**>X79222.1:44-1126 T.vulcanus (Copeland) psbA-I gene**

**ATGACCACAACTCTCCAACGTCGCGAAAGCGCGAATTTGTGGGAGCGGTTTTGTAACTGGGTGACGAGCACCGATAACCGCCTTTATGTGGGCTGGTTTGGGGTGATCATGATCCCCACCCTATTAGCCGCAACCATCTGCTTTGTGATTGCCTTCATCGCTGCTCCCCCTGTGGACATCGATGGCATCCGTGAGCCTGTTTCTGGCTCTTTGCTCTATGGCAACAACATCATCACGGGTGCAGTTGTCCCCTCTAGCAACGCCATTGGCTTGCACTTCTACCCCATTTGGGAAGCTGCTTCCCTCGATGAGTGGCTCTACAACGGTGGCCCCTACCAACTGATCATCTTCCACTTCCTGTTGGGTGCCTCCTGCTACATGGGTCGCCAGTGGGAACTCAGCTACCGCCTCGGTATGCGGCCTTGGATCTGCGTGGCCTACTCTGCCCCCCTGGCTTCTGCCTTTGCAGTCTTCTTGATCTACCCCATTGGTCAAGGCAGCTTCTCTGACGGGATGCCCCTCGGTATCTCTGGTACCTTCAACTTTATGATTGTGTTCCAAGCGGAGCACAACATCCTCATGCACCCCTTCCACCAACTGGGTGTAGCCGGTGTCTTTGGTGGGGCGCTGTTCTGCGCCATGCACGGTTCTTTGGTGACCTCCAGCTTGATCCGTGAAACCACCGAAACCGAATCCGCCAACTACGGTTACAAATTTGGTCAAGAGGAAGAAACCTACAACATCGTGGCTGCCCACGGTTACTTTGGCCGGTTGATCTTCCAATACGCCAGCTTCAACAACAGCCGCTCCCTGCACTTCTTCTTGGCCGCTTGGCGGGTGGTGGGTGTGTGGTTTGCCGCCTTGGGGATCAGCACCATGGCCTTTAACCTGAATGGCTTCAACTTCAACCACTCGGTCATTGATGCCAAGGGCAACGTGATCAACACCTGGGCGGACATCATCAACCGTGCCAACTTGGGTATGGAAGTGATGCACGAGCGCAATGCTCACAACTTCCCCCTCGACTTGGCCAGCGCTGAGTCTGCTCCTGTGGCCATGATTGCTCCCAGCATCAACGGCTAA**

**>S.tuberosum:4099946 K02703 photosystem II P680 reaction center D1 protein [EC:1.10.3.9] | (RefSeq) psbA, SotuCp001; photosystem II protein D1 (N)**

**atgactgcaattttagagagacgcgaaagcgaaagcctatggggtcgcttctgtaactggataactagcactgaaaaccgtctttacattggatggtttggtgttttgatgatccctaccttattgacggcaacttctgtatttattattgccttcattgctgctcctccagtagacattgatggtattcgtgaacctgtttcagggtctctactttacggaaacaacattatttccggtgccattattcctacttctgcagctataggtttacatttttacccaatctgggaagcggcatccgttgatgaatggttatacaacggtggtccttatgaactaattgttctacacttcttacttggcgtagcttgttacatgggtcgtgagtgggagcttagcttccgtctgggtatgcgaccttggattgctgttgcatattcagctcctgttgcagctgctaccgcagttttcttgatctacccaattggtcaaggaagtttttctgatggtatgcctctaggaatctctggtactttcaatttcatgattgtattccaggctgagcacaacatccttatgcacccatttcacatgttaggcgtagctggggtattcggcggctccctattcagtgctatgcatggttccttggtaacttctagtttgatcagggaaaccacagaaaatgaatctgctaatgaaggttacagattcggtcaagaggaagaaacttataatatcgtagccgctcatggttattttggccgattgatcttccaatatgctagtttcaacaactctcgttcgttacacttcttcttagctgcttggcctgtagtaggtatctggtttaccgctttaggtatcagcactatggctttcaacctaaatggtttcaatttcaaccaatctgtagttgacagtcagggtcgtgtaattaacacttgggctgatatcatcaaccgtgctaaccttggtatggaagttatgcatgaacgtaatgctcataacttccctctagacctagctgctatcgaagctccatctacaaatggataa**

**>S.lycopesicum:3950408 K02703 photosystem II P680 reaction center D1 protein [EC:1.10.3.9] | (RefSeq) psbA, LyesC2p089; photosystem II protein D1 (N)**

**atgactgcaattttagagagacgcgaaagcgaaagcctatggggtcgcttctgtaactggataactagcactgaaaaccgtctttacattggatggtttggtgttttgatgatccctaccttattgacggcaacttctgtatttattattgccttcattgctgctcctccagtagacattgatggtattcgtgaacctgtttcagggtctctactttacggaaacaacattatttccggtgccattattcctacttctgcagctataggtttacatttttacccaatctgggaagcggcatccgttgatgaatggttatacaacggtggtccttatgaactaattgttctacacttcttacttggcgtagcttgttacatgggtcgtgagtgggagcttagcttccgtctgggtatgcgaccttggattgctgttgcatattcagctcctgttgcagctgctaccgcagttttcttgatctacccaatcggtcaaggaagtttttctgatggtatgcctctaggaatctctggtactttcaatttcatgattgtattccaggctgaacacaacatccttatgcacccatttcacatgttaggcgtagctggggtattcggcggctccctattcagtgctatgcatggttccttggtaacttctagtttgatcagggaaaccacagaaaatgaatctgctaatgaaggttacagattcggtcaagaggaagaaacttataatatcgtagccgctcatggttattttggccgattgatcttccaatatgctagtttcaacaactctcgttcgttacacttcttcctagctgcttggcctgtagtaggtatctggtttaccgctttaggtatcagcactatggctttcaacctaaatggtttcaatttcaaccaatctgtagttgacagtcagggtcgtgtaattaacacttgggctgatatcatcaaccgtgctaaccttggtatggaagttatgcatgaacgtaatgctcataacttccctctagacctagctgctatcgaagctccatctacaaatggataa**

**>M11005.1:128-1189 Pisum sativum a chloroplast PGII gene encoding a 34.5-kd putative thylakoid membrane protein, complete cds**

**ATGACTGCAATTTTAGAGAGACGCGATAGCGAAAACCTATGGGGTCGCTTCTGTAACTGGATAACCAGCACTGAAAACCGTCTTTACATTGGATGGTTTGGTGTTTTGATGATCCCTACCTTATTGACCGCAACTTCTGTATTTATTATCGCTTTCATTGCTGCCCCTCCAGTAGATATTGATGGTATTCGTGAGCCTGTTTCTGGATCTCTACTTTACGGAAACAATATTATTTCTGGTGCCATTATTCCTACTTCTGCGGCTATCGGTTTGCACTTTTACCCGATATGGGAAGCTGCATCCGTTGATGAATGGTTATACAACGGCGGTCCTTATGAACTAATTGTTCTACACTTCTTACTTGGTGTAGCTTGTTACATGGGTCGTGAGTGGGAACTTAGTTTTCGTCTGGGTATGCGCCCTTGGATTGCTGTTGCATATTCAGCTCCCGTTGCAGCTGCTACTGCAGTTTTCTTAATCTACCCAATTGGTCAAGGAAGCTTTTCAGATGGTATGCCTCTAGGAATCTCTGGTACTTTCAACTTTATGATTGTATTTCAGGCTGAGCATAATATTCTTATGCACCCATTTCACATGTTAGGTGTAGCTGGTGTATTCGGCGGCTCCCTATTCAGTGCTATGCACGGTTCCTTGGTAACTTCTAGTTTGATCAGGGAAACCACAGAAAATGAATCTGCTAATGAAGGTTACCGATTCGGTCAAGAGGAAGAAACCTATAATATTGTAGCTGCTCACGGTTATTTTGGCCGATTGATCTTCCAATATGCTAGTTTCAACAATTCTCGCTCTTTACATTTCTTCCTAGCTGCTTGGCCTGTAGTAGGTATCTGGTTTACCGCGTTAGGTATCAGCACTATGGCTTTCAATTTAAATGGTTTCAATTTTAACCAATCTGTAGTTGATAGTCAAGGTCGTGTAATTAATACCTGGGCTGATATTATTAACCGTGCTAACCTTGGTATGGAAGTTATGCATGAACGTAATGCTCATAATTTCCCTCTAGACCTAGCTGCGGTTGAGGCTCCATCTATAAATGGATAA**

**>N. tabacum:800514 K02703 photosystem II P680 reaction center D1 protein [EC:1.10.3.9] | (RefSeq) psbA, NitaCp001; photosystem II protein D1 (N)**

**atgactgcaattttagagagacgcgaaagcgaaagcctatggggtcgcttctgtaactggataactagcactgaaaaccgtctttacattggatggtttggtgttttgatgatccctaccttattgacggcaacttctgtatttattattgccttcattgctgctcctccagtagacattgatggtattcgtgaacctgtttcagggtctctactttacggaaacaatattatttccggtgccattattcctacttctgcagctataggtttacatttttacccaatctgggaagcggcatccgttgatgaatggttatacaacggtggtccttatgaactaattgttctacacttcttacttggcgtagcttgttacatgggtcgtgagtgggagcttagtttccgtctgggtatgcgaccttggattgctgttgcatattcagctcctgttgcagctgctaccgcagttttcttgatctacccaattggtcaaggaagtttttctgatggtatgcctctaggaatctctggtactttcaatttcatgattgtattccaggctgagcacaacatccttatgcacccatttcacatgttaggcgtagctggtgtattcggcggctccctattcagtgctatgcatggttccttggtaacttctagtttgatcagggaaaccacagaaaatgaatctgctaatgaaggttacagattcggtcaagaggaagaaacttataacatcgtagccgctcatggttattttggccgattgatcttccaatatgctagtttcaacaactctcgttcgttacacttcttcctagctgcttggcctgtagtaggtatctggtttaccgctttaggtatcagcactatggctttcaacctaaatggtttcaatttcaaccaatctgtagttgacagtcaaggccgtgtaattaatacttgggctgatatcattaaccgtgctaaccttggtatggaagttatgcatgaacgtaatgctcacaacttccctctagacctagctgctatcgaagctccatctacaaatggataa**

**>NC_002762.1:c1152-91 Triticum aestivum, PSII D1 chloroplast, complete genome**

**ATGACTGCAATTTTAGAGAGACGCGAAAGTACAAGCCTGTGGGGTCGCTTCTGCAACTGGATAACTAGCACTGAAAATCGTCTTTACATCGGATGGTTCGGTGTTTTGATGATCCCTACCTTATTGACCGCAACTTCTGTATTTATTATCGCCTTCATCGCTGCCCCTCCAGTAGATATTGATGGTATTCGTGAGCCTGTTTCTGGTTCTTTACTTTATGGAAACAATATTATCTCTGGTGCTATTATCCCTACTTCTGCGGCGATCGGATTGCACTTTTACCCAATTTGGGAAGCTGCATCTGTTGATGAGTGGTTATACAATGGTGGTCCTTATGAGCTAATTGTTCTACACTTCTTACTTGGTGTAGCTTGTTATATGGGTCGTGAGTGGGAACTTAGTTTCCGTCTGGGTATGCGTCCTTGGATTGCTGTTGCATATTCAGCTCCTGTTGCAGCTGCCACTGCTGTTTTCTTGATTTACCCTATTGGTCAAGGAAGCTTTTCTGATGGTATGCCTTTAGGAATCTCTGGTACTTTCAACTTTATGATTGTATTCCAGGCAGAGCATAACATCCTTATGCATCCATTCCACATGTTAGGTGTAGCTGGTGTATTCGGCGGTTCCCTATTCAGTGCTATGCATGGTTCCTTGGTAACCTCTAGTTTGATCAGGGAAACTACTGAAAATGAATCTGCTAATGAGGGTTACAAATTTGGTCAAGAGGAAGAAACTTATAATATTGTGGCTGCTCATGGTTATTTTGGCCGATTAATCTTCCAATATGCTAGTTTCAACAACTCTCGTTCTTTACACTTCTTCTTGGCTGCTTGGCCTGTAGTAGGAATCTGGTTCACTGCTTTAGGTATTAGTACTATGGCTTTCAACCTAAATGGTTTCAATTTCAACCAATCTGTAGTTGATAGTCAAGGTCGCGTTATTAATACTTGGGCTGATATCATCAACCGTGCTAACCTTGGTATGGAAGTAATGCACGAACGTAATGCTCACAACTTCCCTCTAGACTTAGCTGCTGTTGAAGTTCCATCTATTAATGGATAA**

**>ana:alr4866 K02703, Nostoc sp. PCC 7120, photosystem II P680 reaction center D1 protein [EC:1.10.3.9] | (GenBank) psbAI; photosystem II protein D1 (N)**

**atgaccacaaccttacaacagcgtagtagcgctaacgtatgggaacggttctgcacatggatcaccagcaccgaaaaccgcatttatgttggttggttcggcgtgttgatgattcctaccctgctagccgctaccgtctgcttcatcatcgctttcgttgctgcacctccagtagacattgatggtatccgtgaaccagtagcaggttccttgatctacggaaacaacatcatctctggtgcagttgttccttcctccaacgctattggtttgcacttctaccccatctgggaagcagcttccttagatgagtggttgtacaacggtggcccttaccaattggtaattttccacttcttgatcggatgcgcttgctacctcggtcgtcagtgggaattgtcttaccgtttaggtatgcgtccttggatctgcgtagcttactctgcacctttggcatctgctacagcagtattcttgatctaccccatcggacaaggttccttctctgatggtatgcccttgggtatctccggcaccttcaacttcatgatcgtgttccaagcagaacacaacatcctgatgcaccccttccacatgttgggtgtagctggtgtattcggtggttccttgttctctgcaatgcacggttctttggtaacttcctccttggttcgtgaaacaaccgaaatcgaatctcagaactacggttacaaattcggtcaagaagaagaaacctacaacatcgttgcagcgcacggctacttcggtcgtttgatcttccaatacgcttcctttaacaacagccgccaactgcacttcttcctagctgcatggcctgtaatcggtatctggtttaccgcgttgggcgtaagcacaatggcgttcaacttgaacggttttaacttcaaccaatccatcatcgactcacaaggtcgtgtaatcaatacctgggctgacatcatcaaccgcgctaacttgggtatggaagtaatgcacgagcgtaacgctcacaacttccctctagacttagctgctggtgaagttgctcctgttgcgttaaccgctcctgctatcaacggttaa**

**>C. reinhardtii, ChreCp021 K02703 photosystem II P680 reaction center D1 protein [EC:1.10.3.9] | (RefSeq) psbA; photosystem II protein D1 (N)**

**atgacagcaattttagaacgtcgtgaaaattctagcctatgggctcgtttttgtgagtggatcacttcaactgaaaaccgtttatacatcggttggttcggtgtaatcatgatcccatgtcttcttactgcaacatcagtattcatcatcgctttcatcgctgctccgccagtagacatcgatggtatccgtgaaccagtttcaggttctcttctttacggtaacaacatcattacaggtgctgtaatcccaacttctaacgcaatcggtcttcacttctacccaatttgggaagctgcttctctagacgagtggttatacaacggtggtccttaccaacttatcgtttgtcacttccttctaggtgtatactgctacatgggtcgtgagtgggaattatctttccgtttaggtatgcgtccatggatcgctgtagcttactcagctccagtagctgcagcttcagctgtattcttagtttaccctatcggccaaggttcattctctgacggtatgcctttaggtatctctggtactttcaacttcatgatcgtattccaagcagaacacaacatccttatgcacccattccacatgttaggtgttgctggtgtattcggtggttcattattctcagctatgcacggttctttagttacttcatctttaatccgtgaaacaactgaaaacgaatcagctaacgaaggttaccgtttcggtcaagaagaagaaacttacaacattgtagctgctcatggttactttggtcgtctaatcttccaatacgcttctttcaacaactctcgttcattacacttcttcttagctgcttggccggtaatcggtatttggttcactgctttaggtttatcaactatggcattcaacttaaacggtttcaacttcaaccaatcagtagtagactcacaaggtcgtgtactaaacacttgggcagacatcatcaaccgtgctaacttaggtatggaagtaatgcacgagcgtaacgctcacaacttccctctagacttagcttcaactaactctagctcaaacaactaa**

**>V. carteri, photosystem II, D1**

**atgacagcaatcttagaacgtcgtgaaaattctagcctatgggctcgtttttgtgagtggatcacttcaactgagaaccgcctttacatcggttggttcggtgtaatcatgatcccatgtcttcttactgcaacatcagtatttatcatcgctttcatcgctgctccgccagtagacatcgatggtattcgtgaaccagtttcaggttctcttctttatggtaacaacatcatcacaggtgctgttgttccaacttctaacgctatcggtctgcatttctacccaatttgggaagctgcttctctagatgagtggttatacaacggtggtccttaccagcttatcgtttgccacttccttctaggtgtatactgctacatgggtcgtgagtgggaactttcattccgtttaggtatgcgtccttggatcgctgtagcttactcagctccagtagctgctgcttcagctgtattcttagtatacccaatcggtcagggttcattctcagacggtatgccgttaggtatttcaggtactttcaacttcatgatcgtattccaggctgaacataacattcttatgcacccattccacatgttaggtgttgctggtgtattcggtggttcattattctcagctatgcacggttcattagttacttcatctctaatccgtgaaacaactgaaaacgaatcagctaacgaaggttaccgcttcggtcaagaagaagagacttacaacatcgtagctgctcatggttactttggtcgtctaatcttccaatacgcttcattcaacaactcacgttcattacacttcttcttagctgcttggccagtaatcggtatttggttcacagctttaggtctatcaacaatggcgttcaacttaaacggtttcaacttcaaccaatcagttgtagactcacaaggtcgtgtacttaacacttgggctgatatcatcaaccgtgctaacttaggtatggaagttatgcacgaacgtaacgctcacaacttcccgcttgacttagcttctaacaacaattcttctatgaactaa**

**>Crocosphaera subtropica ATCC 51142:cce_0636 K02703 photosystem II P680 reaction center D1 protein [EC:1.10.3.9] | (GenBank) psbA5; photosystem II D1 protein (N)**

**atgactactaccttacaacaacgcgagagcgtttccttgtgggaacagttttgtcagtgggtgaccagcaccaacaaccgcatctatgtcggttggttcggtaccttaatgatccccaccctcttaactgctaccacctgtttcatcattgctttcatcgctgctcctcctgtggacatcgatggtatccgtgagcctgtagctggttctttactctacggaaacaacatcgtttccggtgcagtcgtaccttcttctaatgctatcggtttacacttctatcccatctgggaagctgcttcacttgatgagtggctttacaatggcggtccttaccagttagtaattttccacttcttaattggaatcttttgctacatgggtcgtcagtgggaactttcctaccgtttaggaatgcgtccttggatctgtgttgcctactctgcacctgtatccgcagcgaccgcagtattcttaatctaccccatcggacaaggttctttctctgatggtatgcctttaggaatctccggaaccttcaacttcatgttcgtgttccaagctgaacacaacatcttgatgcaccccttccatatgttgggtgttgctggtgtattcggtggatctttattctccgctatgcacggttccttagtaacctcttctttagttcgtgaaaccactgaaatcgagtctcaaaactacggttacaagttcggacaagaagaagaaacctacaacatcgtagctgctcacggttactttggtcgtttaatcttccaatacgcatccttcaacaacagccgtgctttacacttcttcttaggtgcatggcctgtaatcggtatctggttcaccgcaatgggtgtatccaccatggctttcaacttaaacggatttaacttcaaccagtctatccttgattctcaaggccgtgtaatcggaacctgggctgatgtattaaaccgtgcaggaatcggaatggaagtaatgcacgagcgtaacgctcacaacttccccttagacttagcttctgctgagcctgtatctgctcctgtaatcaatggttaa**

**>Gloeobacter violaceus PCC 7421:gvip089 K02703 photosystem II P680 reaction center D1 protein [EC:1.10.3.9] | (RefSeq) psbA; photosystem II protein D1 (N)**

**atgactgcaactcttgaacgtcgttcttcacaagggctgtgggaccgcttcgccgattgggtgacctccaccaacaaccgcttctacgttggttggttcggcgtcctgatgatccccaccctgctttctgcgacgatttgcttcgtcgtcgcttttgtcgccgccccgccggtggacatggacggcatccgcgaaccgatttccggttcgctgctgtacggcaacaacatcatcaccggggcggtgatcccgtcttccaacgccatcggcctgcacttctacccaatttgggaagcggcttcgatggacgagtggctctacaacggcggcccctaccagctggtggtcttccattttctcattggggtgttttgctacctgggccgcgagtgggagctcagctaccgtttgggcctgcgtccgtggatctgcatcgcctacagtgctcctgtggcggcggcggcggcggtcttcttgatttacccgattggccagggcagcttcagtgacggtatgccgttgggtatttcgggcacgttcaacttcatgtttgtttttcaggcggagcacaacattctgaaccatcctttccacatgctgggggtggcgggtgtgttcggcggttcgctgttcagtgcgatgcacggttcgctggtgacttcttcattgattcgtgagacgtcgatggaagagtcccagaactacggatacaagtttggccaggaagaggagacgtacaacatcattgcggcgcatggttactttggccgtttgattttccagtacgccagcttcaacaacagccgcagtttgcactttttcttggcggcgtggccggtgattgggatttggttcacggcgctgggcatcagtgtgatggcgttcaacctgaacggcttcaacttcaacagcagcatcgtggattctcagggtcgtgcgatatacacgtgggcggacatcgtcaaccgagcgaatttgggaatggaagtgatgcacgagcgcaatgctcacaacttccccttggatttggctggaacggagtcggctccggtggcggtgggcaacgccgacctcaacggctaa**

**>Geitlerinema sp. PCC 7407 :GEI7407_0907 K02703 photosystem II P680 reaction center D1 protein [EC:1.10.3.9] | (GenBank) photosystem q(b) protein (N)**

**atgactacaacactccaacgtcgggagggtggtagcctatgggagcgtttttgcagctgggtgaccagcaccgacaaccgcctgtatgtgggctggttcggtgtgctgatgatccccacgttgctgaccgcaaccgtttgcttcatcatcgcttttgttgcagcaccccccgtcgacatcgacggtatccgtgagcccgttgctggctccctgctgtacggcaacaacatcatctctggtgctgttgttccttcttccaacgctatcggcctccacttctaccccatctgggaagctgcttccctcgatgagtggctgtacaacggcggcccttaccagctcgtcgtttgccacttcctcatcggcatcagctgctacatggggcgtcagtgggaactgagctaccgcctgggcatgcgtccttggatctgcgttgcttacagcgctccgctgtctgcagctttcgcagtgttcctgatctacccgatcggccaaggcagcttctctgacggtatgcctctgggcatcagcggcaccttcaacttcatgttcgtgttccaagctgagcacaacatcctgatgcaccccttccacatgctcggtgtggctggtgtcttcggtggttcgctgttctccgccatgcacggctctctggtgacctcttctctggtgcgtgaaaccaccgagaacgagtctcagaacgctggctacaagttcggtcaagaagaagagacctacaacatcgttgcagcccacggctacttcggtcgtctcatcttccaatacgcttctttcaacaacagccgttctctgcacttcttcctcggcgcttggccggtggtaggcatctggttcacgtcgatgggcatcagcaccatggcgttcaacctgaacggtttcaacttcaaccagtcggtcttggatagccaaggtcgagtggtcggcacctgggctgacgtgctcaaccgcgcgaacctgggtatggaagtgatgcacgagcgcaacgctcacaacttcccccttgacctggcttctggcgatgaaatgccggttgctctgcaagctcctgccatccacggctaa**

**>P. trichocarpa:4929629 K02703 photosystem II P680 reaction center D1 protein [EC:1.10.3.9] | (RefSeq) psbA, Poptr_cp001; photosystem II protein D1 (N)**

**atgactgcaattttagagagacgcgaaagcgaaagcctttggggtcgcttctgtaattggataaccagcactgaaaaccgtctttatattggatggtttggtgttttgatgattcctactttattgaccgcaacttctgtatttattatcgctttcattgctgcccctccagtagacattgatggtattcgtgaacctgtttctggatctctactttatggaaacaatatcatttctggtgccattattcctacttctgcggctatagggttgcacttttacccaatatgggaagcggcatccgttgatgaatggttatacaacggcggtccttatgagctaattgttctacacttcttacttggtgtagcttgttacatggggcgtgagtgggaacttagtttccgtctgggtatgcgtccttggattgctgttgcatattcagctcctgttgcggcggctactgctgttttcttgatttatccaatcggtcaaggaagtttttcggatgggatgcctctaggaatctctggtacttttaactttatgattgtattccaggctgagcacaacatccttatgcacccatttcacatgttaggcgtagctggtgtattcggcggctccctattcagtgctatgcatggttctttggtaacctctagtttgatcagggaaaccacagaaaatgaatctgctaatgaaggttacagatttggtcaagaggaagaaacttataatatcgtagccgctcatggttattttggccgattgatcttccaatatgctagttttaacaactctcgttctttacatttcttcttagctgcttggcctgtagtaggtatctggttcactgctttaggtattagcactatggctttcaatctaaatggtttcaatttcaaccaatctgtagttgatagtcaaggtcgtgtaattaatacctgggctgatattattaaccgtgctaaccttggtatggaagttatgcatgaacgtaatgctcataatttccctctagacctagctgctgtcgaagttccatctacaaatggataa**

**>Gloeobacter kilaueensis JS1:GKIL_0727 K02703 photosystem II P680 reaction center D1 protein [EC:1.10.3.9] | (GenBank) psbA; photosystem II protein D1 (N)**

**atgactgcaacacttgagcgtcgttcgactcagggattgtgggagcgcttcgccgactgggtcacctccaccaacaaccgcttctacgtcggttggttcggcgtcttgatgatccccaccctgctttcggctaccatctgctacatcgtcgccttcatcgccgccccgccggtggacatggacggcatccgcgagccgatttccggctccttgctttacggcaacaacatcatcaccggcgctgtcattcccagctccaacgctatcggtcttcacttttatccgatttgggaagcagccagcatggatgagtggctctacaacggcgggccttaccagcttgtcgtcttccactttttgattggggtgttttgctacctgggtcgggagtgggagttatcgtatcgtctgggcttgcgtccctggatctgcattgcctacagtgcgcctgtggcggcagcgacggcggtgtttttgatttacccgattggtcagggcagtttcagcgatgggatgccgttgggtatctccggcacgttcaacttcatgtttgtctttcaggcggagcacaacattctcaaccaccccttccacatgctgggagtggcgggagtgttcggtggttcgcttttcagtgcgatgcacggttcgttggtgaccagcagcctgattcgtgagacttcttacgaggagtcgcagaactacggttacaagtttggtcaggaagaggagacgtacaacatcattgcggcccacggctactttggtcgtctgattttccagtacgcgagcttcaacaacagccgttcgttgcactttttcctggcggcgtggccggttgtagggatctggttcacggcgctgggcatcagcgtgatggcgttcaacctgaacgggttcaacttcaattcgagcatcgtggattcgcagggccgtgcgatttacacgtgggcggacgtggtgaaccgggcgaacctgggaatggaagtgatgcacgagaggaatgcgcacaatttcccgctggacctggcgtcgagcgagagtgtgccggtggcggtgagcaccgctgacctcaacggctaa**

**>D84228.1:577-1659 Microcystis aeruginosa psbA2 gene for D1 protein, complete cds**

**ATGACTACAACTCTACAACAGCGCGAGAGCGCTTCCCTGTGGGAGCAGTTCTGCCAGTGGATCACCAGCACCAACAACCGTCTTTATGTCGGTTGGTTCGGTGTCATCATGATCCCCACCCTGCTCACCGCCACCACCTGCTTCATCATCGCCTTTATCGCCGCTCCTCCCGTAGATATCGACGGTATTCGCGAGCCTGTAGCTGGTTCTCTACTCTACGGAAACAACATCATCTCTGGTGCGGTTGTTCCCTCCTCCAACGCAATTGGACTCCACTTTTACCCCATCTGGGAAGCTGCTTCCTTAGATGAGTGGTTATACAACGGTGGTCCCTACCAGTTAGTCATTTTCCACTTCTTACTAGGTGTCTTCTGCTACCTCGGTCGTCAGTGGGAACTGTCTTTCCGTTTAGGAATGCGTCCTTGGATTTGTGTAGCTTACTCTGCACCCGTATCCGCCGCTACTGCTGTATTCTTAATCTACCCCATCGGACAAGGTTCCTTCTCTGATGGTATGCCTTTAGGAATCTCTGGAACCTTCAACTTTATGTTCGTGTTCCAAGCAGAACATAACATCCTGATGCACCCCTTCCATATGTTAGGTGTTGCTGGTGTGTTCGGCGGTTCTCTGTTCTCCGCGATGCACGGTTCCCTAGTAACTTCTTCCTTAGTGCGTGAAACCACTGAAATCGAATCTCAAAACTACGGTTACAAATTCGGTCAAGAGGAAGAAACCTACAATATCGTTGCCGCTCACGGTTACTTCGGACGTTTAATCTTCCAATACGCTTCTTTCAACAATAGCCGCTCTTTGCACTTCTTCTTAGGTGCTTGGCCGGTAATCGGTATCTGGTTTACGGCAATGGGTGTTAGCACCATGGCGTTCAACCTCAACGGTTTCAACTTCAACCAGTCGATTCTCGATTCTCAAGGTCGTGTAATCGGTACTTGGGTCGATGTGTTAAACCGCGCTGGTATCGGTATGGAAGTAATGCACGAGCGCAATGCTCACAACTTCCCCTTAGACTTGGCTAGTGGTGAACAGGCTCCTGTGGCTCTGACCGCTCCCGCTATCAATGGTTAA**

**>fig|224013.5.peg.5465|ACX27_22810| Photosystem II protein D1 (PsbA) [Nostoc piscinale CENA21 | 224013.5]**

**atgacagcaaccttacaacagcgccgtagcgccaacgtatgggagcagttctgcaactggatcaccagcaccaacaaccgcctatatattggctggttcggcgtattaatgatcccaaccttgctagctgcaaccacctgcttcgtaatcgccttcatcgcagcacctccagtagacatcgatggtatccgtgaaccagtagctggttccttactctacggaaacaacatcatctccggtgcagttgttccttcctccaacgcaattggattacacttctacccaatttgggaagcagcatctcttgatgagtggttgtacaacggtggcccttaccaactagtaatattccacttcctgattggcgtattctgctacttaggacgtgagtgggaattgtcttaccgcttgggaatgcgtccttggatctgcctagcattctccgctcccgtagcggctgctaccgcagtattcttgatttaccccatcggacaaggttccttctctgacggtatgcccttgggaatcagtggtaccttcaacttcatgatcgtgttccaagcagaacacaacatcctgatgcaccccttccacatgcttggtgtggctggtgtattcggtggttctctgttctccgcaatgcacggttccttggtaacttcctccttagttcgtgaaacaaccgagaacgaatcccaaaactacggatacaaattcggtcaagaagaagaaacctacaacatcgttgcagcacacggttacttcggtcgcttaatcttccaatacgcgtccttcaacaacagccgttccttgcacttcttcctggcagcatggcctgtaatcggcatctggttcacagcgttgggcgtaagcacaatggcgttcaacctcaacggtttcaacttcaaccaatcagtgattgactctcaaggtcgcgtcatcaacacctgggctgacatcatcaaccgcgctaacttgggtatggaagtcatgcacgagcgcaatgctcacaacttccccttagacttggctgctggtgagcaagctcctgttgctctgactgctcctgctatcaatggctaa**

**>Oscillatoria acuminata PCC 6304:Oscil6304_3571 K02703 photosystem II P680 reaction center D1 protein [EC:1.10.3.9] | (GenBank) photosystem II DI subunit, Q(B) protein (N)**

**atgaccacaactttacagcagcgcgaaagcgctaatctgtgggaccgcttctgcgaatgggtcgcttctaccgaaaaccgcctttatatcggctggttcggcgtgttgatgatccctactctcttaagcgctaccgtttgttacatcatcgccttcatcgctgctcctcctgtggacattgatggaatccgcgaacctgttgccggttctctgttgtacggaaacaacatcatctctggtgcggttgttccttcctctaacgcgatcggtcttcacttctaccccatttgggaagcagccagcttggatgagtggctctacaatggtggcccttaccagcttgtgattttccacttcctcattggcatcttctgctacatgggtcgtgagtgggaactctcctaccgcttaggaatgcgtccttggatctgcgttgcttactctgcacctgttgcagccgcttccgcagtgttcttgatctacccgatcggacaaggttctttctctgatggtatgcccttgggtatctctggaaccttcaacttcatgttggtgttccaagctgaacacaacatcctgatgcaccccttccatatgttgggtgtggccggtgtgttcggtggttccttgttcagtgccatgcacggttctctggttaccagttctttggttcgtgaaaccagcgaaaccgaatctcaaaactacggttacaaattcggtcaagaagaagaaacctacaacattgttgccgctcacggttactttggtcgtttaatcttccaatatgcttctttcaacaacagccgttccttgcacttcttcttagctgcttggccggttgtgggtatctggttcaccgctttgggtgtgtccacgatggcctttaacttgaacggatttaacttcaaccagtccatcatcgactcgacaggtcgtgttgtgaatacctgggctgatgtgattaaccgggctaacctgggtatggaagtgatgcacgagcgtaatgctcacaacttcccccttgatttggctgctggcgaagcaactccggttgctttgactgctccttctatcaatggttaa**

**>Nostoc sp. ATCC 29411 / PCC 7524:Nos7524_0680 K02703 photosystem II P680 reaction center D1 protein [EC:1.10.3.9] | (GenBank) photosystem II DI subunit, Q(B) protein (N)**

**atgaccacaaccttacaacagcgcaaaagcgccaatgtatgggagcagttctgcgagtggatcaccagcaccgacaaccggatttacatcggttggttcggcgtgttaatgatccccaccctgctagctgcaaccacctgcttcatcatcgccttcatcgctgcacctccagtagacatcgatggtatccgcgaacccgttgcaggttccttaatctacggaaacaacatcatctctggtgcagttgttccttcttctaacgctatcggtttgcacttctaccccatctgggaagcagcttccttagatgagtggttgtacaacggtggtccttaccaattggtaattttccacttcttgatcggttgcgcttgctacctaggtcgtcagtgggaattgtcctaccgcttaggtatgcgtccttggatctgcgtagcatactctgcacctttggcatctgctacagcagtattcttgatctaccccatcggtcaaggttccttctctgacggtatgcccttgggtatctccggcaccttcaacttcatgatcgtgttccaagcagaacacaacatcctgatgcaccccttccacatgttaggtgtggctggtgtattcggcggtagcttgttctctgcaatgcacggttccttggtaacttcctccttggtgcgtgaaaccaccgaaaccgaatctcaaaactacggttacaaattcggtcaagaagaagaaacctacaacatcgttgcagcccacggctacttcggtcgtttgatcttccaatacgcatctttcaacaacagccgttccttgcacttcttcttggctgcatggccagtagtaggcatctggttcaccgcattgggcatcagcacaatggcgttcaacttgaacggtttcaacttcaaccaatccgtgattgactcccaaggtcgcgttatcaacacctgggctgatatcatcaaccgcgctaacttgggtatggaagtaatgcacgagcgtaacgctcacaacttccccttagacttggctgctggtgaagttgctcctgttgctttaactgcacctgcaatcaacggctaa**

**>SIHH01000170.1:c2315-1254 Phormidium sp. SL48-SHIP NODE_14125_length_8693_cov_65.550590, PsbA D1, whole genome shotgun sequence**

**ATGACAACCACACTTCAACAACAGCAATCCGCCTCCCTCTGGGAACGGTTTTGCGGTTGGGTCACCAGCACCAACAACCGCCTTTACATCGGCTGGTTCGGTGTCTTAATGATCCCGACCCTGCTCACCGCCACCACCTGCTTCATCATCGCCTTCATCGCCGCTCCCCCCGTGGACATCGATGGTATCCGCGAACCGGTGGCTGGTTCTCTACTCTACGGAAACAACATCATCTCCGGTGCCGTTGTTCCTTCTTCCAACGCGATCGGACTTCACTTGTACCCCATCTGGGAAGCTGCCAGCCTCGACGAATGGCTCTATAACGGCGGTCCTTACCAGCTCGTGATTCTTCACTTCCTCATTGGCGTCTTCTGCTACATGGGTCGTGAATGGGAACTGTCCTTCCGTCTCGGAATGCGTCCCTGGATTTGCGTAGCGTACTCCGCACCCGTTGCCGCTGCCACCGCAGTCTTTCTGATTTACCCCATTGGTCAAGGTTCCTTCTCCGATGGTATGCCTCTCGGCATCTCGGGAACCTTCAACTTCATGTTGGTGTTCCAAGCTGAGCACAACATCCTGATGCACCCCTTCCATATGCTCGGTGTGGCGGGTGTCTTCGGTGGTGCCTTGTTCTCCGCCATGCACGGTTCTCTCGTGACCTCCTCCTTGGTTCGTGAAACCACCGAGAGTGAATCTCAGAACTACGGCTACAAATTCGGTCAAGAAGAAGAAACCTACAACATTGTTGCAGCTCACGGCTACTTCGGTCGCCTGATCTTCCAATACGCTTCCTTCAACAACAGCCGCTCTCTGCACTTCTTCTTAGGTGCATGGCCGGTAGTTGGCATCTGGTTCACCGCCTTAGGTATCAGCACCATGGCGTTCAACCTCAACGGCTTCAACTTCAACCAATCCGTGTTGGATAGCCAAGGTCGCGTCATCAACACCTGGGCTGATGTCATCAACCGTGCCAACCTGGGTATGGAAGTGATGCACGAGCGCAACGCTCACAACTTCCCCCTCGACTTGGCAGCCACTGAAGCGCCTTCGATTAACGGCTAG**

**>Prochlorococcus marinus (MIT 9301):P9301_02451 K02703 photosystem II P680 reaction center D1 protein [EC:1.10.3.9] | (GenBank) psbA; Photosystem II PsbA protein (D1) (N)**

**atgacaactattcagcagcagcgttcttcgctgttaaaaggttggccacagttttgtgagtgggtaacatcaactaacaacagaatttatgttggttggttcggcgtcttaatgatcccatgcctacttacagcagcggcttgcttcatcgttgcattcatcgcagcaccaccagtagacatcgacggaattagagagccagttgctggttcattcctatatggaaacaacatcatctcaggtgcagttgttccttcttctaacgctattggtctacacttctacccaatttgggaagcagctactgtagatgagtggttatacaacggtggtccttaccagcttgtaattttccacttcctaattggtatttcagcatacatgggaagacagtgggagctttcataccgcttaggtatgcgtccatggatctgtgttgcatactctgcaccagtttcagcagctttcgcagtatttcttgtatacccattcggtcaaggttcattctctgacggaatgcctctaggtatctctggaacattcaacttcatgtttgttttccaggcagagcacaacattctaatgcacccattccacatggctggtgttgctggtatgttcggaggatctctattctcagctatgcatggttcacttgttacttcatctctaatcagagaaacaactgagacagaatctcagaactatggttacaagttcggacaagaagaagaaacatacaacatcgttgcagctcatggctacttcggtcgtttgatcttccaatatgcttcattcaacaacagcagaagtcttcacttcttcctagctgtattcccagttgtttgtgtatggttaacttcaatgggtatctgcacaatggcattcaaccttaacggtttcaacttcaaccagtcagttgttgatgcaaacggtaagattgttcctacatggggtgacgttcttaacagagcaaacctaggtatggaagtaatgcacgagcgtaacgctcacaacttcccacttgatctagcagcagctgagtctacaacagtagctctttcagctccagctatcggttaa**

**>Physcomitrella patens subsp. patens:2546772 K02703 photosystem II P680 reaction center D1 protein [EC:1.10.3.9] | (RefSeq) psbA, PhpapaCp046; photosystem II protein D1 (N)**

**atgactgctactttagaaagacgcgaaagcgcaagcctatggggtcgcttctgcgactgggttaccagcactgaaaaccgcctttacatcggatggttcggtgttttaatgattcctactctattaactgcaacttctgtatttattattgctttcattgcagctcctcctgtagatattgatggtatccgtgagcctgtttctggttctcttctttacggaaacaacatcatctctgctgctatcatccctacttctgcagctatcggtttgcacttctacccaatttgggaagctgcttccgttgatgaatggctttacaacggtggtccttacgagttaatcgttcttcacttcttacttggtgtagcttgctacatgggtcgtgagtgggaacttagctaccgtttaggtatgcgtccttggatcgctgttgcatattcagctcctgttgcggctgctactgctgttttcttgatctacccaattggtcaaggaagcttctctgacggtatgcctttaggaatctctggtactttcaacttcatgattgtgttccaagctgaacacaacatccttatgcacccattccacatgcttggtgtagctggtgtattcggtggctctctattcagtgctatgcatggttccttagtaacttcaagtttaatccgtgaaactactgagaatgagtctgctaacgcaggttacaagtttggtcaagaggaagaaacttacaacatcgtagctgctcacggttactttggtagattaattttccaatacgctagctttaacaactctcgttctttacacttcttcttagctgcttggcctgtagtaggtatctggttcactgcattaggtatcagcactatggctttcaacttaaacggtttcaacttcaaccaatctgttgttgacagtcaaggtcgtgttattaacacttgggctgacatcatcaaccgtgctaaccttggtatggaagttatgcatgaacgtaacgctcacaacttccctctagacttagcttctgttgaagctccttctgtaaacggttaa**

**>X52758.1:1-1083 C.caldarium (G. sulphuraria) plastid DNA of psbA gene**

**ATGACAGCTACATTAGAAAGACGTCAAACAGCAAGTTTATGGGAACGTTTTTGTTCTTGGATAACTAGCA**

**CAGAAAATCGCCTATACATAGGTTGGTTTGGTGTATTGATGATACCTACATTATTAACAGCTACATCTGT**

**ATTTATAATTGGTTTTATAGCTGCTCCACCTGTTGATATAGATGGTATTAGAGAACCAGGTTTCAGGTCC**

**TTATTATATGGAAATAATATTATAACCGGTGCTATTGTACCTACATCTAATGCAATAGGAATACACTTTT**

**ATCCTATTTGGGAAGCAGCATCATTAGATGAATGGTTATATAATGGTGGTCCTTATGAATTAATAGTTTT**

**GCACTTCTTTATTGGAATTTGTGCATATATGGGACGTGAATGGGAATTAAGTTATCGTCTTGGAATGCGT**

**CCTTGGATCGCTGTAGCTTTTTCAGCTCCTGTTGCTGCCGCCACAGCTGTTTTTATTATATATCCTATCG**

**GACAAGGTAGTTTTTCAGATGGTATGCCATTAGGTATTTCTGGTACTTTTAATTTTATGTTAGTTTTCCA**

**AGCTGAACATAATATTTTAATGCATCCATTCCATATGATGGGTGTTGCTGGTGTATTTGGTGGTTCACTA**

**TTTAGTGCAATGCATGGTTCCTTGGTAACTTCTAGTTTGATCCGTGAAAGAACAGAGAATGAGTCTGCTA**

**ACAATGGTTATAAATTCGGTCAAGAATATGAAACTTATAACATCGTTGCTGCTCATGGTTATTTTGGAAG**

**ATTAATTTTCCAATATGCAAGTTTTAATAATTCACGTTCATTACATTTCTTCTTAGCTTTATGGCCAGTA**

**GTATGTATTTGCGTTACAGCTCTAGGTGTTAGCACCATGGCATTTAACTTAAACGGATTCAATTTCAACC**

**AATCTGTTGTTGATTCTCAAGGAAGAGTAATTAATACTTGGGCTGATATTTTAAATCGTGCAAATTTAGG**

**AATAGAAGTAATGCACGAACGTAATGCACATAACTTCCCTCTAGATTTAGCAAGCGAGGTATCTTTACCA**

**GTTGCTTTAAATAAAGTAGAAATAAATGGTTAA**

**>AF227740.1:34-1116 Vaucheria litorea photosystem II subunit core 32 kD protein D1 (psbA) gene, complete cds; chloroplast gene for chloroplast product**

**ATGACTGCAACTTTAGAAAGACGTGAAAGCATTAGCTTATGGGAACGTTTCTGTTCTTGGATCACTAGTA**

**CTGATAACCGTTTATACATCGGTTGGTTTGGTGTATTAATGATTCCTACATTATTAACTGCAACTACTTG**

**TTATATCATTGCATTTATTGCTGCTCCTCCAGTAGATATTGACGGTATTCGTGAACCAGTTGCTGGTTCA**

**TTATTATATGGAAACAACATCATTTCTGGTGCTGTAATTCCTTCATCTAACGCTATTGGTGTACACTTCT**

**ACCCAATTTGGGAAGCTGCTTCAGTAGATGAGTGGTTATATAACGGTGGTCCTTACCAATTAATCGTATT**

**ACATTTTTTATTAGGTGTAGCTAGTTATATGGGTCGTGAGTGGGAATTAAGCTACCGTTTAGGTATGCGT**

**CCATGGATCTTTGTAGCATTTTCTGCTCCTGTAGCAGCAGCTTCAGCTGTATTCTTAGTATATCCAATTG**

**GTCAAGGTTCATTCTCTGATGGTATGCCATTAGGTATTTCAGGAACTTTTAACTTTATGTTAGTATTCCA**

**AGCAGAACATAACATTTTAATGCACCCATTCCACATGGCTGGTGTTGCTGGTGTATTTGGTGGTTCATTA**

**TTCTCTGCTATGCACGGTTCTTTAGTAACTTCAAGTTTAATTCGTGAAACTAGCGAAGTAGAATCTACAA**

**ACTATGGTTACAAATTCGGTCAAGAAGAAGAAACTTATAACATCGTAGCTGCACATGGTTACTTTGGTCG**

**TTTAATCTTCCAATATGCATCATTCAATAATTCTCGTGCATTACACTTCTTCTTAGCTGCATGGCCTGTA**

**GTTGGTATTTGGTTAACTGCTTTAGGTGTAAGTACAATGGCTTTCAACTTAAACGGTTTCAACTTTAACC**

**AATCAGTAGTAGATAGTCAAGGTCGTGTAATTAACACTTGGGCAGATATTATTAACCGTGCTGATTTAGG**

**TATGGAAGTAATGCATGAACGTAATGCGCACAACTTCCCATTAGATTTAGCTGCTGGTGAAATTCTTCCT**

**GTAGCAGTAACTGCTCCAGTAATAGCTGGTTAA**

**Notes S8.** Coding sequences (CDSs) of *psbD* (D2)

**>NC_015288.1:183142-184197 Prochlorococcus phage Syn1, PsbD on complete genome**

**ATGACTACAAGCACACTAACAACACCAACGAGGGGGTGGTTCGATGTCCTTGATGACTGGGTTAAACGCGATCGCTTTGTCTTTGTGGGCTGGTCTGGACTCCTACTTCTTCCCACTGCTTATCTTGCCATTGGCGGTTGGCTTACTGGCACAGCTTTTGTTACGAGTTGGTATACCCATGGACTTGCTTCTTCCTATCTTGAAGGTGCTAACTTTCTTACGGCAGCTGTCTCGACGCCTGCTGACGCTATGGGTCATTCTCTTCTGTTACTTTGGGGTCCTGAGTCTCAAGGGGACTTTCAACGCTGGTGCCAACTTGGGGGACTCTGGAATTTTGTGGCACTCCACGGTGCCTTTGCTCTCATTGGTTTCATGCTTCGACAGTTTGAACTTGCTCGCTTGATTGGTATCCGTCCTTACAATGCTATTGCGTTCTCTGGTCCTATTGCTGTTTTTGTCAGTGTATTCCTTATCTATCCACTGGGTCAGTCTTCATGGTTCTTTGCACCTAGTTTCGGGGTAGCAGCAATCTTTAGATTCCTATTGTTCCTTCAGGGTTTCCACAACTGGACGTTAAACCCCTTCCATATGATGGGAGTTGCTGGTATACTAGGAGGAGCACTACTCAGTGCTATCCATGGTGTCACAGTAGAGAATACATTGTATGAAGATGGAGAACAAGCAAACACATTCAAGGCATTTGATTCCACTCAAGAAGAAGAGACGTATTCTATGGTTACAGCGAATCGTTTCTGGTCACAAATCTTCGGGGTTGCGTTTAGCAATAAGCGTTGGTTGCACTTCTTTATGTTGTTTGTTCCTGTCATGGGTCTTTGGGTCTCTTCTATTGGCATCATTGGGCTTGCTCTTAATCTTCGTGCTTATGATTTTGTAAGTCAAGAGATCAGAGCAGCAGAAGATCCAGAATTTGAGACGTTCTACACAAAGAACATACTTCTTAATGAAGGACTACGTGCATGGTTAGCACCTTCTGATCAACCACATGAGAATTTTATATTTCCAGAGGAGGTATTACCTAGAGGCAATGCTCTTTGA**

**>NC_015286.1:168444-169499 Synechococcus phage Syn19, PsbD on complete genome**

**ATGGTCGCTTCAACACTAAGTCCCCCGAGGAGGGGGTGGTTCGATGTCCTCGATGACTGGCTTAAACGGGATCGTTTCGTTTTTGTTGGTTGGTCTGGACTTCTTCTTTTTCCCACTGCTTATCTTGCTATTGGCGGTTGGCTTACTGGGACTGCTTTCGTCACGAGTTGGTATACTCACGGGTTGGCAAGTTCCTATCTGGAGGGCGCAAACTTTCTTACTGCGGCAGTTAGTACTCCAGCAGATTCTATGGGTCATTCTCTTCTGCTTCTCTGGGGTCCTGAGGCACAAGGGGATTTCGTCAGATGGTGTCAACTTGGAGGACTCTGGGCTTTTGTGGCGCTCCACGGGGCTTTCGCTCTAATCGGTTTCATGCTTCGTCAGTTTGAACTGGCACGTCTCATCGGTATCCGTCCCTACAATGCTATTGCGTTCTCTGGTCCTATCGCTGTTTTTGTTAGCGTCTTTCTCATCTACCCTCTGGGTCAATCGAGTTGGTTTTTCGCTCCATCTTTCGGGGTCTCAGCAATCTTCCGATTCCTTCTCTTCCTACAAGGATTCCATAACTGGACACTGAATCCATTTCATATGATGGGTGTAGCAGGTATTTTGGGAGGAGCCTTGCTTTCTGCCATTCATGGTGTTACAGTAGAGAATACTCTGTATCAAGATGGTGAACAAGCAAATACTTTCAAAGCATTTGACAGCACTCAGGAAGAGGAGACCTATTCAATGGTTACTGCAAACCGTTTCTGGTCTCAGATCTTTGGTATTGCGTTCAGTAATAAGAGGTGGTTGCATTTCTTTATGCTGTTTGTTCCTGTTATGGGTCTTTGGACAAGTTCCATCGGTATTATTGGTCTTGCTCTCAACCTTCGTGCTTATGACTTTGTTTCCCAAGAGATCAGAGCAGCAGAAGATCCAGAGTTTGAGACGTTCTACACCAAGAACATCCTCTTGAACGAAGGTCTTCGTGCATGGTTAGCACCTATTGATCAACCTCATGAGAACTTTGTGTTCCCTGAGGAAGTACTTCCCCGAGGTAATGCCCTGTGA**

**>NC_015285.1:163582-164637 Prochlorococcus phage Syn33, PsbD on complete genome**

**ATGACTACAACAACACTAAAAGCACCAACAAGGGGGTGGTTTGATGTCCTGGATGACTGGCTTAAGCGCGATCGTTTCGTATTTATTGGTTGGTCTGGATTACTACTTCTTCCCACTGCTTATATGGCAATTGGTGGCTGGCTTACTGGTACAACTTTCGTTACGTCGTGGTACACCCATGGACTGGCAACTTCCTATCTTGAGGGTGCTAATTTTCTCACAGCGGCTGTGTCAACGCCTGCTGACGCTATGGGTCATTCTCTTCTTCTTCTCTGGGGTCCTGAGGCTCAGGGCGATTTCGTCCGCTGGATCCAACTTGGGGGACTCTGGAATTTTGTGGCACTCCACGGAGCCTTTGCTCTCATTGGTTTCATGCTTCGGCAATTTGAACTTGCTCGCCTGATTGGAATCAGACCCTACAATGCGATTGCTTTCTCTGGTCCTATTGCTGTCTTCGTTAGTGTCTTTCTCATCTACCCTCTGGGTCAATCAAGTTGGTTCTTCGCTCCATCTTTTGGAGTGGCAGCAATCTTCAGATTCCTCTTATTCCTTCAAGGATTTCATAACTGGACTTTGAACCCCTTCCATATGATGGGAGTTGCTGGTATACTAGGCGGAGCATTGCTATCTGCTATTCACGGAGTCACAGTTGAAAATACATTGTATGAAGATGGAGAACAAGCAAACACCTTTAAGGCGTTTGATTCGACACAGGAAGAAGAGACTTATTCTATGGTCACTGCAAACCGCTTCTGGTCGCAGATCTTCGGTGTTGCGTTTTCTAACAAGCGGTGGCTTCATTTTTTCATGTTGTTTGTGCCTGTTATGGGTCTATGGACATCCTCTATTGGCATTATTGGCCTTGCTCTCAATCTTCGTGCTTACGATTTTGTAAGTCAAGAGATTAGAGCAGCAGAAGATCCTGAGTTCGAGACTTTCTATACCAAGAACATCTTATTGAACGAAGGACTCCGTGCATGGTTAGCACCAGTTGACCAGCCAGGAGAACGGTTTGTCTTCCCAGAAGAAGTACTGCCGAGAGGTAATGCGCTTTAA**

**>NC_015282.1:165155-166210 Synechococcus phage S-SM1, PsbD on complete genome**

**ATGGTTGCATCAACACTAAGTCCCCCACGGAGGGGGTGGTTCGATGTCCTCGATGACTGGCTTAAACGAGATCGTTTCGTTTTTGTTGGTTGGTCTGGACTTCTTCTTTTTCCCACTGCTTATCTTGCTATTGGCGGTTGGCTTACTGGGACTACTTTCGCGACGAGTTGGTATACTCACGGGTTGGCAAGTTCCTATCTGGAGGGTGCAAACTTTCTTACTGCGGCAGTTTCTACTCCAGCAGACGCTATGGGTCATTCTCTTCTGCTTCTCTGGGGTCCTGAGGCTCAAGGGGATTTCGTCAGGTGGATCCAACTTGGGGGACTCTGGGCTTTTGTGGCGCTCCACGGGGCTTTCGCTCTAATTGGTTTCATGCTTCGTCAGTTTGAATTAGCACGTCTCATCGGTATCCGTCCCTACAATGCTATTGCGTTCTCTGGTCCTATCGCTGTTTTTGTTAGCGTCTTTCTCATCTACCCTCTGGGTCAATCGAGTTGGTTTTTCGCTCCATCTTTCGGGGTCGCAGCAATCTTCAGGTTCCTTCTCTTCCTACAAGGATTCCATAACTGGACCTTGAATCCCTTTCATATGATGGGTGTTGCAGGTATCCTGGGTGGTGCATTGCTTTCTGCCATCCATGGTGTTACAGTAGAGAATACTCTGTATCAAGATGGTGACGATGCAAATACTTTCAAGGCATTTGACTCTACTCAAGAGGAAGAAACCTACTCAATGGTTACTGCAAACCGTTTCTGGTCTCAGATCTTCGGTATTGCGTTTAGTAATAAGAGGTGGTTGCATTTCTTTATGCTGTTTGTTCCTGTTATGGGTCTTTGGACAAGTTCCATCGGTATTATTGGTCTTGCTCTCAACCTTCGTGCTTATGACTTTGTATCCCAAGAGATCAGAGCAGCAGAAGACCCAGAGTTTGAAACCTTCTACACGAAAAACATTCTCTTGAATGAAGGTCTTCGTGCTTGGTTGGCACCAGTCGATCAACCGCATGAGAACTTTGTCTTCCCTGAAGAAGTTCTGCCTAGGGGTAACGCGCTGTGA**

**>NC_015281.1:169268-170323 Synechococcus phage S-ShM2, PsbD on complete genome**

**ATGACGACAAGCACACTAAACATACCAACTAGGGGGTGGTTCGATGTTCTCGATGACTGGCTCAAACGCGACCGCTTTGTATTCATTGGTTGGTCTGGTCTACTTCTTCTTCCCACTGCCTATCTTGCTATTGGAGGGTGGCTCACTGGTACAGCGTTTGTTACTTCCTGGTACACCCACGGACTCGCTTCCAGTTACTTAGAAGGCGCTAACTTCCTCACTGCTGCTGTCAGTACACCTGCTGACGCTATGGGACACTCACTGCTCTTGCTATGGGGTCCAGAAGCACAAGGCGATTTCGTTCGCTGGTGTCAATTGGGTGGTCTCTGGAACTTTGTAGCACTTCATGGTGCATTTGCATTGATTGGTTTCATGCTTAGGCAGTTTGAATTGGCACGTCTTATTGGCATCCGTCCTTATAATGCTATTGCATTCTCTGGTCCTATTGCTGTATTTGTCAGTGTCTTTCTTATCTACCCACTTGGACAGTCCTCATGGTTCTTTGCTCCCTCCTTTGGAGTATCAGCAATCTTCAGGTTCCTGTTGTTCCTGCAAGGATTCCACAACTGGACGTTGAATCCATTCCACATGATGGGTGTAGCAGGAATCTTGGGCGGAGCATTGCTTTCTGCTATCCATGGTGTTACAGTAGAGAACACATTGTATGAAGACGGTGAGCAGGCAAATACTTTCAAGGCGTTTGATTCGACACAGGAAGAAGAGACTTATTCTATGGTCACTGCAAACCGCTTCTGGTCGCAGATCTTCGGTATTGCGTTTTCTAACAAGCGTTGGCTTCATTTTTTCATGTTGTTTGTGCCTGTTATGGGTCTTTGGACATCCTCTATTGGCATTATTGGCCTTGCTCTCAATCTTCGTGCTTACGATTTCGTGAGTCAGGAGATCAGAGCAGCAGAAGATCCAGAATTTGAGACCTTCTACACCAAAAACATCCTATTGAATGAAGGACTTCGTGCATGGTTGGCACCTGCTGATCAACCACATGAAAACTTCATCTTCCCAGAAGAAGTTCTTCCGAGAGGTAACGCACTGTGA**

**>NC_019516.1:9770-10831 Cyanophage S-TIM5, PsbD on complete genome**

**ATGGTTGCTTCAACATTAACACAACAATCACAACGGAGGGAATGGTTTGACATCCTTGATGACTGGCTTAAACGAGATCGCTTTGTTTTTGTGGGTTGGTCTGGACTACTTCTTTTTCCCACTGCTTATCTTGCAATTGGTGGCTGGCTTACTGGCACTACGTTTGTCACGAGTTGGTACACTCACGGACTTGCAAGTAGTTACCTTGAGGGTGCTAATTTCCTTACAGCGGCTGTCTCAACGCCTGCTGATGCTATGGGTCATTCTCTTCTTCTACTTTGGGGTCCTGAGTCTCAGGGAGATTTCGTCCGCTGGATCCAGCTTGGGGGACTCTGGGCTTTTGTGGCGCTCCACGGAGCCTTCGCTTTAATCGGCTTTATGTTACGCCAGTTTGAGCTGGCGAGATTAATTGGAATCCGACCTTACAATGCTATCGCGTTTTCTGGGCCTATTGCTGTTTTTGTCAGTGTTTTCCTCATCTATCCACTCGGACAGTCTAGTTGGTTCTTTGCGCCGAGTTTCGGCGTCGCGGCGATTTTCCGCTTCCTTCTCTTCCTCCAGGGCTTTCATAATTGGACGCTCAATCCCTTCCATATGATGGGAGTTGCTGGTATACTGGGAGGGGCGCTCCTATCAGCAATCCACGGAGTCACTGTAGAAAACACACTTTATCAAGATGGCGAACAAGCAAACACTTTCAAAGCTTTTGATAGCACTCAAGAAGAAGAGACCTATTCGATGGTTACTGCTAACCGTTTCTGGTCCCAGATATTTGGAATTGCTTTTAGCAATAAACGCTGGCTTCATTTCTTTATGCTGTTTGTACCTGTTATGGGTCTTTGGACCTCTTCTATTGGAATCATTGGTCTTGCTCTCAATCTTCGTGCTTACGATTTTGTATCTCAAGAGATCAGGGCGTCAGAAGATCCTGAATTTGAGACGTTCTATACGAAGAATATCCTTCTGAACGAAGGTCTTCGTGCATGGATGGCACCAGTTGACCAACCACACGAAAGCTTTGTTTTCCCAGAAGAAGTTCTTCCTAGAGGTAATGCACTGTGA**

**>NC_047734.1:187824-188879 Cyanophage S-RIM44 isolate Np_42_0711, PsbD on complete genome**

**ATGACAACTTCAACACTAACAACACCAACGAGGGGGTGGTTCGATGTCCTTGATGACTGGGTTAAACGCGATCGCTTTGTCTTTGTGGGCTGGTCTGGACTCCTACTTCTTCCCACTGCTTATCTTGCCATTGGCGGTTGGCTTACTGGCACAGCTTTTGTTACGAGTTGGTATACCCATGGACTTGCTTCTTCCTATCTTGAAGGTGCTAACTTTCTTACGGCAGCTGTCTCGACGCCTGCTGACGCTATGGGTCATTCTCTTCTGTTACTTTGGGGTCCTGAGTCTCAAGGGGACTTTCAACGCTGGTGCCAACTTGGGGGACTCTGGAATTTTGTGGCACTCCACGGTGCCTTTGCTCTCATTGGTTTCATGCTTCGACAGTTTGAACTTGCTCGCTTGATTGGTATCCGTCCTTACAATGCTATTGCGTTCTCTGGTCCTATTGCTGTTTTTGTCAGTGTATTCCTTATCTATCCACTGGGTCAGTCTTCATGGTTCTTTGCACCTAGTTTCGGGGTAGCAGCAATCTTTAGATTCCTATTGTTCCTTCAGGGTTTCCACAACTGGACCCTCAACCCCTTCCATATGATGGGAGTTGCTGGTATACTAGGAGGAGCACTACTCAGTGCTATCCATGGTGTCACAGTAGAGAATACATTGTATGAAGATGGAGAACAAGCAAACACATTCAAGGCATTTGATTCCACTCAAGAAGAAGAGACGTATTCTATGGTTACAGCGAATCGTTTCTGGTCACAAATCTTCGGGGTTGCGTTTAGCAATAAGCGTTGGTTGCACTTCTTTATGTTGTTTGTTCCTGTCATGGGTCTTTGGGTCTCTTCTATTGGCATCATTGGGCTTGCTCTTAATCTTCGTGCTTATGATTTTGTAAGTCAAGAGATCAGAGCAGCAGAAGATCCTGAATTTGAAACTTTCTACACTAAGAACATCTTATTGAATGAAGGACTACGTGCATGGTTAGCACCTTCTGATCAACCACACGAAAACTTTATCTTCCCTGAGGAAGTTCTGCCTCGTGGTAATGCTCTTTGA**

**>NC_047733.1:162083-163138 Synechococcus phage S-RIM8 isolate RW_22_0300, PsbD on complete genome**

**ATGACAACAAGTACACTACAATTACCAAGAAGGGGGTGGTTTGATGTCCTTGACGACTGGCTTAAAAGAGACCGTTTCGTTTTTGTTGGCTGGTCTGGACTTCTTCTTTTTCCCACTGCTTATCTTGCTCTTGGGGGTTGGCTTACTGGGACTACTTTCGTCACGAGTTGGTATACTCACGGACTTGCGAGTTCCTATCTTGAGGGTGCAAACTTTCTTACTGCGGCAGTTTCTACTCCAGCAGACGCTATGGGTCATTCTCTTCTGCTTCTATGGGGTCCTGAGTCTCAAGGGGACTTCATCCGCTGGTGCCAACTTGGGGGACTCTGGACTTTTGTGGCGCTCCACGGAGCCTTTGCACTTATAGGTTTCATGCTTCGCCAGTTTGAACTGGCACGACTTATCGGTATTCGTCCTTACAATGCTATTGCTTTCTCAGGTCCTATTGCTGTATTCGTTAGTGTTTTTCTTATCTATCCTTTGGGACAATCGTCCTGGTTCTTCGCACCGTCGTTCGGGGTCGCAGCAATCTTCCGATTCCTGCTCTTCCTCCAAGGGTTCCATAACTGGACGCTAAATCCTTTTCACATGATGGGCGTAGCAGGTATCCTTGGTGGTGCATTGCTCTCTGCTATTCATGGAGTGACAGTTGAAAATACACTGTACGAAGATGGCGAACAAGCAAACACTTTTAAAGCATTTGACTCCACACAGGAGGAAGAAACGTATTCGATGGTTACTGCGAACCGCTTCTGGTCACAGATTTTCGGTATTGCTTTTAGTAACAAGCGTTGGTTGCATTTCTTTATGCTCTTTGTTCCCGTCATGGGTCTCTGGACAAGTTCTATCGGTATTATTGGACTCGCTCTCAATCTTCGTGCTTACGACTTTGTATCTCAAGAAGTTCGTGCGGCAGAAGACCCTGAGTTTGAGACGTTCTATACAAAGAACATTCTTCTGAATGAAGGACTTCGAGCATGGTTAGCACCAGTGGACCAACCTCATGAGAACTTTGTGTTCCCTGAAGAGGTCTTGCCAAGAGGCAACGCTCTATGA**

**>NC_031935.1:178797-179855 Synechococcus phage S-WAM2 isolate 0810PA29, PsbD on complete genome**

**ATGGTCGCAAGCACACTAACACTACAGAAACAAAGGGGGTGGTTTGATGTACTCGATGACTGGCTTAAGCGTGATCGGTTCGTTTTTGTTGGTTGGTCAGGTCTTCTTCTGTTCCCTACTGCTTACATGGCACTGGGCGGTTGGCTCACGGGCACAACATTCGTCACCTCGTGGTATACTCACGGGATTGCTTCTTCGTATCTTGAGGGATGTAATTTCCTTACTGCTGCTGTATCAACTCCTGCTGACGCTCTCGGACATTCCCTACTTCTTCTATGGGGTCCAGAAGCTCAGGGAGATTTCGTCCGCTGGCTCCAACTTGGGGGACTATGGCCTTTTGTGGCGCTCCACGGTGCCTTCGCTCTCATAGGTTTCATGCTTCGGCAGTTTGAAATCGCACGTCTAGTCGGTATTCGTCCTTACAATGCAATCGCGTTTTCTGGTCCAATTGCTGTCTTTGTGTCTGTTTTTCTCATGTATCCTCTCGGACAGTCCTCGTGGTTCTTTGCGCCGTCGTTTGGAGTTGCAGCGATTTTCCGCTTCCTACTCTTCCTACAGGGCTTCCACAACTGGACGCTCAACCCGTTCCACATGATGGGTGTAGCAGGTATCCTGGGCGGAGCATTGCTTTGTGCCATTCATGGTGCTACAGTAGAGAACACACTCTTTGAAGACAGTGACCAATCAAATACATTCAAGGCATTCGAGCCTACACAAGAAGAGGAGACCTATTCGATGGTTACTGCCAACCGTTTCTGGTCTCAGATCTTTGGTATCGCCTTCAGCAATAAGCGTTGGCTACATTTCTTTATGTTGTTTGTTCCTGTCATGGGTCTTTGGACCTCTAGCATTGGTATTATTGGTCTTGCACTTAACCTTAGAGCTTATGACTTTGTTTCACAAGAGATCAGAGCGGCAGAGGATCCTGAGTTCGAGACGTTCTATACCAAGAACATCCTATTGAATGAAGGTCTACGTGCTTGGATGGCACCAGTCGATCAACCACATGAAAACTTTGTATTTCCTGAAGAAGTATTGCCAAGAGGCAACGCTCTGTGA**

**>NC_031922.1:163848-164903 Synechococcus phage S-CAM9 isolate 1109NB16, PsbD on complete genome**

**ATGACAACAAGTACACTAAACATTCCACAAAGGGGGTGGTTCGATGTCCTGGACGACTGGCTTAAACGAGATCGCTTTGTCTTTGTGGGTTGGTCTGGATTACTTCTTTTTCCCACTGCTTATCTTGCAATTGGTGGCTGGCTTACTGGTACAACGTTTGTTACCAGTTGGTACACCCACGGTCTCGCGTCTAGTTACCTTGAGGGTGCTAATTTCCTTACAGCGGCTGTGTCAACGCCTGCTGATGCTATGGGTCATTCTCTTCTTCTACTTTGGGGTCCTGAGTCTCAGGGGGACTTCATTAGGTGGTGCCAACTTGGGGGACTCTGGGCTTTTGTGGCGTTCCACGGATCTTTCGCTCTGATCGGTTTCATGCTTCGGCAGTTTGAAATTGCACGTCTTGTAGGTATCCGTCCGTACAATGCTATTGCGTTCTCTGGTCCGATTGCTGTCTTTGTCAGCGTTTTCCTTATGTACCCTCTCGGGCAATCTAGTTGGTTCTTTGCCCCCTCGTTTGGGGTCGCGGCGATCTTCAGATTCCTCTTGTTCCTCCAAGGTTTCCATAACTGGACACTCAACCCCTTCCATATGATGGGTGTTGCAGGCATCCTTGGTGGTGCTCTTCTCTGTGCTATTCATGGTGCTACCGTAGAGAATACTTTGTTTGAAGATGGTGAACAATCAAATACTTTCAAGGCATTCGAGCCTACTCAAGAGGAAGAGACTTACTCGATGGTCACTGCCAACCGCTTCTGGTCTCAGATTTTTGGTATTGCTTTCAGCAATAAGCGTTGGTTGCACTTCTTTATGCTCTTTGTTCCTGTCATGGGACTTTGGACAAGTTCTATTGGTATCATCGGTCTCGCACTCAATCTTCGTGCTTATGATTTTGTGTCTCAGGAGATTAGAGCAGCAGAGGATCCTGAATTTGAGACGTTCTATACGAAGAACATCCTCCTCAATGAAGGACTCCGTGCCTGGATGGCTCCAGTAGACCAGCCACACGAATCGTTTGTATTTCCAGAGGAAGTTCTTCCGAGAGGTAACGCACTCTAA**

**>NC_031906.1:189948-191003 Synechococcus phage S-CAM3 isolate 1010CC42, PsbD on complete genome**

**ATGGTCGCTTCAACACTACAACAACCGAGGAGGGAATGGTTTGACATCCTTGATGACTGGCTTAAACGAGATCGCTTTGTCTTTGTGGGTTGGTCTGGATTACTTCTTTTTCCCACTGCTTATCTCGCAATTGGTGGCTGGCTTACTGGCACGACGTTTGCTACAAGCTGGTACACCCACGGACTCGCAAGTAGTTACCTTGAGGGTGCTAATTTTCTTACAGCGGCTGTGTCAACGCCTGCTGATGCTATGGGTCATTCTCTTCTTCTACTTTGGGGTCCTGAATCTCAAGGCGATTTCGTCAGGTGGATCCAACTTGGGGGACTCTGGGCTTTTGTGGCGCTCCACGGGGCTTTCGCTCTAATTGGATTCATGCTTCGTCAGTTTGAACTGGCACGTCTCATCGGTATCCGTCCTTACAATGCTATTGCGTTCTCTGGTCCTATCGCTGTTTTTGTTAGCGTCTTTCTCATCTACCCTCTGGGTCAATCGAGTTGGTTCTTCGCGCCATCCTTCGGTGTGGCAGCGATCTTCCGCTTCCTCCTCTTCTTGCAGGGCTTCCATAACTGGACGCTGAACCCCTTCCACATGATGGGTGTTGCAGGTATCCTGGGAGGAGCACTACTGTCAGCAATCCATGGTGTGACTGTAGAGAATACTTTGTATGAAGATGGTGAACAAGCAAACACGTTTAAAGCATTCGACTCTACACAAGAGGAAGAGACCTATTCGATGGTCACTGCCAACCGTTTCTGGTCTCAAATCTTTGGTGTTGCGTTCAGTAATAAGAGGTGGTTGCATTTCTTTATGCTCTTTGTTCCTGTTATGGGTCTGTGGACTTCTTCCATCGGTATTATTGGTCTTGCTCTCAACCTTCGTGCTTATGACTTTGTATCCCAAGAGATCAGAGCAGCAGAAGATCCAGAGTTCGAGACTTTTTACACAAAAAATATACTTTTGAATGAAGGTCTTCGTTCATGGTTGGCACCTGTTGATCAACCCCATGAGAATTTTGTATTTCCAGAAGAAGTATTGCCAAGAGGCAACGCTCTGTGA**

**>NC_031242.1:165114-166169 Cyanophage S-RIM50 isolate RW_29_0704, PsbD on complete genome**

**ATGGTAGCAAGCACACTAAGTCCCCCAAGGAGGGGTTGGTTCGATGTCCTTGACGACTGGCTTAAAAGAGACCGTTTCGTTTTTGTTGGCTGGTCTGGACTTCTTCTTTTTCCCACTGCTTATCTTGCTCTTGGGGGTTGGCTTACTGGGACTACTTTCGTCACGAGTTGGTATACTCACGGACTTGCGAGTTCCTATCTTGAGGGTGCAAACTTTCTTACTGCGGCAGTTTCTACTCCAGCAGACGCTATGGGTCATTCTCTTCTGCTTCTATGGGGTCCTGAGTCTCAAGGGGACTTCATCCGCTGGTGCCAACTTGGGGGACTCTGGACTTTTGTGGCGCTCCACGGAGCCTTTGCACTTATAGGTTTCATGCTTCGCCAGTTTGAACTGGCACGACTTATCGGTATTCGTCCTTACAATGCTATTGCTTTCTCTGGTCCTATTGCTGTATTCGTTAGTGTTTTTCTTATGTATCCTCTGGGACAATCGTCCTGGTTCTTCGCACCGTCGTTCGGGGTCGCAGCAATCTTCCGATTCCTGCTCTTCCTCCAAGGGTTCCATAACTGGACGCTGAATCCCTTTCACATGATGGGCGTAGCAGGTATCTTAGGCGGAGCATTGCTTTCTGCCATTCATGGTGTTACAGTAGAGAATACTTTGTATGAAGATGGTGAACAAGCAAACACTTTCAAAGCATTTGATAGTACTCAGGAAGAGGAAACTTATTCCATGGTCACTGCTAACCGTTTCTGGTCTCAGATTTTTGGTATTGCTTTCAGCAATAAGCGTTGGCTTCACTTCTTTATGCTCTTTGTTCCTGTCATGGGACTTTGGACAAGTTCTATTGGTATCATTGGTCTCGCACTCAATCTTCGTGCTTATGATTTTGTGTCTCAGGAGATTAGAGCTGCTGAGGATCCAGAGTTTGAAACGTTCTATACGAAAAACATCCTCCTTAATGAAGGACTCCGTGCCTGGTTGGCTCCTGTAGACCAACCTCATGAGAACTTTGTGTTCCCTGAAGAAGTCTTGCCAAGAGGCAACGCACTTTAA**

**>A. thaliana:ArthCp017 K02706 photosystem II P680 reaction center D2 protein [EC:1.10.3.9] | (RefSeq) psbD; photosystem II protein D2 (N)**

**atgactatagcccttggtaaatttaccaaagacgaaaaagatttatttgatattatggatgactggttacggagggaccgcttcgtttttgtaggttggtctggtctattgctctttccttgtgcctatttcgctttagggggttggttcacaggtacaacctttgtaacttcatggtatactcatggattggccagttcctatttagaaggttgcaattttttaaccgctgcagtttctactcctgctaatagtttagcgcattctttgttgttactgtggggtcctgaagcacaaggagattttactcgttggtgtcaattaggtggtctatgggcttttgttgctctccacggcgctttcgcattaataggttttatgttacgtcaatttgaacttgctcgatctgttcaattgcgaccttataatgcaatcgcattctctggtcccattgctgtttttgtttctgtcttcctaatttatccactaggtcaatctggttggttctttgcgcctagttttggtgtagcggctatatttcgattcatcctctttttccaagggtttcataattggacattgaacccatttcatatgatgggagtcgccggtgtactgggcgcggctctgctatgcgctattcatggtgctactgtagaaaatactttatttgaagatggtgatggtgcaaatacattccgtgcttttaacccaactcaagccgaagaaacttattcaatggtcaccgctaaccgcttttggtcacaaatctttggggttgctttttccaataaacgttggttacatttctttatgttatttgtaccggtaactggtttatggatgagtgctcttggagtagtcggtttagctttgaacctacgtgcctatgacttcgtttcccaggaaatccgtgcagcggaagatccagaatttgagactttctatactaaaaatattcttttaaacgaaggtattcgtgcttggatggcggctcaagatcagcctcatgaaaaccttatattccctgaggaggttctaccacgtggaaacgctctttaa**

**>S. oleracea:2715610 K02706 photosystem II P680 reaction center D2 protein [EC:1.10.3.9] | (RefSeq) psbD, SpolCp018; photosystem II protein D2 (N)**

**atgactatagccgttggtaaatttaccaaagacgaaaaagatttatttgatagtatggatgactggttacggagggaccgttttgtttttgtaggttggtctggtctattgctctttccttgtgcttatttcgccttagggggttggtttacgggtacaacctttgtaacttcatggtatacccatggattggccagttcttatttggaaggctgcaatttcttaaccgcagcagtttctactcctgctaatagtttagcccactctttattgttactgtggggtcctgaagcacaaggggattttactcgttggtgtcaattaggtggcctgtgggcttttgttgctcttcatggtgctttcgcactaataggttttatgttacgtcaattcgaacttgctcggtctgttcaattgcgcccttataatgcaatcgcattctctggtccaattgctgtttttgtttctgtattcctgatttatccactaggtcaatctggttggttctttgcgcctagttttggtgtagcagctatattccgattcattctcttttttcaagggtttcataattggacgctgaacccatttcatatgatgggagttgccggtgtattgggcgccgctctgctatgcgctattcatggtgctaccgttgaaaatactttatttgaagatggtgatggggcaaatacattccgggcttttaacccaacccaagctgaagaaacttattcaatggtcaccgctaaccgcttttggtcccaaatctttggggttgctttttccaataaacgttggttacatttctttatgttatttgtaccagtaaccggtttatggatgagtgctcttggagtagtcggtctggctttgaacctacgtgcctatgacttcgtttcccaggaaatccgtgcagctgaagatcctgaatttgaaactttttacaccaaaaatattctcttaaacgagggtatccgtgcttggatggcggctcaagatcagcctcatgaaaaccttatattccctgaggaggttctaccccgtggaaacgctctttaa**

**>T. elongatus:tlr1630 K02706 photosystem II P680 reaction center D2 protein [EC:1.10.3.9] | (RefSeq) psbD1; photosystem II reaction center D2 protein (N)**

**atgacgatcgcgattggacgagcgccagcggaacggggatggtttgacatcctcgacgactggctcaaacgggacagatttgtctttgtcggctggtcaggcatcctgcttttcccctgcgcctacctggcgctggggggctggctgaccggtaccacctttgtgacctcctggtacacccacggcctggcctccagctacctagaagggtgcaacttcctcaccgttgccgtttccacccccgccaacagcatggggcactccctgctcctcctgtggggacctgaagcccaaggggactttacccgctggtgccaactgggtggtctgtggacctttatcgccctccacggcgccttcggtctcattgggttcatgctgcggcagtttgaaattgcccgcttggtgggcgtccgtccctacaacgccattgccttcagcgcccccattgccgtctttgtcagcgtcttcttgatctatcccttggggcaatccagctggttctttgcccccagctttggcgtcgccgccatcttccgtttcctgctctttttccaagggttccacaactggaccttgaaccccttccacatgatgggggtagccggtgtgctagggggtgccctgttgtgtgccatccacggcgccacggtggaaaataccctcttccaagatggagaaggggcgagcaccttccgtgccttcaatcccacccaagcggaagagacctactcgatggtgacggcgaaccgtttttggagccaaatttttgggattgccttctcgaacaagcgctggttgcactttttcatgttgtttgtgccggtgacggggctgtggatgagtgcgattggcgtggtgggtctagcgttgaacctgcggtcctatgacttcatttcgcaggagattcgggctgcggaagaccctgaatttgagacgttctacacgaagaacctgctgttgaacgagggtatccgcgcttggatggcgccccaagaccaaccccatgaaaactttgtcttcccagaagaggtactcccccgtggtaacgctctctag**

**>Synechocystis PCC 6803:slr0927 K02706 photosystem II P680 reaction center D2 protein [EC:1.10.3.9] | (GenBank) psbD2; photosystem II D2 protein (N)**

**atgaccattgcagtcggacgcgccccagtcgaaagaggatggtttgatgtcctcgacgattggctaaagcgtgatcgtttcgtatttatcggttggtctggtttgctactcttcccctgcgccttcatggccctggggggatggttaaccggcaccaccttcgttacttcctggtacacccacggtctagccagttcctacctggaaggggctaactttttgaccgtggcggtctcttcccccgccgatgccttcggccattccctcctgttcctgtggggaccggaagctcaaggtaacctgacccgctggttccaaattggtggtttgtggcccttcgttgccctccacggtgcctttggattgattggcttcatgctgcgtcagttcgaaatttcccgtctggtaggcattcgtccctacaacgccatcgctttctctggtcccattgcggtatttgtcagcgtctttctgatgtaccccttgggtcaatcgagttggttctttgctcccagctttggggtagcgggaatcttccggtttattttgttcctacaaggtttccacaactggaccctgaaccccttccacatgatgggagtagccggtattctcggtggtgccctactgtgtgccatccacggtgccacggtggaaaacaccctgtttgaagacggtgaagattccaacaccttccgggcgtttgaacctacccaagcggaagaaacctactccatggtgactgccaaccgtttctggtctcagattttcggtattgctttctccaacaaacggtggctgcacttcttcatgttgttcgttcccgtaactggtttgtggatgagttctgtgggtatcgtcggtttggcgttgaacctacgggcttatgacttcgtttcccaggaactgcgggctgctgaagatccggaatttgaaacgttttatacgaaaaacattttgttgaacgaagggatgcgcgcctggatggctccccaagatcaaccccatgaaaactttatcttccctgaggaagtactgccccggggtaatgctctctaa**

**>S. elongatus PCC 7942:Synpcc7942_0655 K02706 photosystem II P680 reaction center D2 protein [EC:1.10.3.9] | (GenBank) photosystem II D2 protein (photosystem q(a) protein) (N)**

**atgacgattgcagtagggcgagcgccagcggagcggggatggtttgacgtcctcgacgactggctgaagcgcgaccgatttgtatttgtgggttggtcagggttgctgctgtttccctgtgcgtatttagcactgggcgggtggttgaccgggaccagctttgtgacgtcgtggtacacccacggcatcgcgtcttcgtacttagaaggcggcaactttttgaccgtagcagtgagcaccccagcggatgcgtttgggcattcgttgatgctgctgtggggccccgaggcacaagggaacttcgtgcgttggtgccagttgggtggcttgtggaacttcgtagcactgcacggcgccttcggcctgattgggttcatgctgcgtcaatttgagattgcgcggttggtgggcgtccgtccgtacaacgcgatcgccttttcgggtccgatcgcagtgttcgtgtcggtgttcttgatgtacccgttgggtcaatcgagctggttcttcgctccgagctttggcgtggcagcgattttccggtttttgttgttcctgcaagggttccacaactggaccttgaacccattccacatgatgggcgtggccgggattttgggtggggcattgctgtgcgccattcacggtgcgacggtggagaacaccctgttcgaggattcagagcaatcgaacaccttccgggcatttgagccgacgcaggccgaagagacgtactcgatggtgacggcgaaccgtttttggagccagattttcgggattgcgttttcgaacaagcggtggctgcactttttcatgctgttcgtgccggtgacgggcttgtggatgagctcgatcgggattgtaggtttggcgttgaacctgcgggcgtacgacttcgtgtcgcaggagctgcgggccgctgaggatccggaatttgagacgttctacacgaagaacatcttgttgaacgaagggattcgggcctggatggcaccgcaagaccaaccgcacgaaaaattcgtcttccccgaagaggttctgccccgtggtaacgctctctag**

**>E. gracilis ENA|CAA50076|CAA50076.1 Euglena gracilis PSII D2-polypeptide**

**ATGACTTTTACAGATTTAAATACTGAAAATAAAAACGGTTGGTTCGACGTAGCAGACGATTGGTTGAAAAAAGACAGATTTATTTTTATTGGTTGGTCTGGATTGCTGTTATTTCCTTGTTCATATCTTGCTTTAGGTGGATGGTTAACGGGAATTACATTTGTTACTTCATGGTATACACATGGATTAGCTAGCTCTTTCCTTGAAGGTTGCAATGCTTTGACAGCAGCAGTTTCAACACCTCCAAATAGTATGGGTCATTCTTTATTATTGCTTTTGGGGTCTGAAGCACAGTGGGATTTTACTCGTTGGTTGCAAATAGGAGGACTATGGCCATTTATTGCTCTTCATGGTGCTTTTGGGTTGATAGGCTTTATGTTGCGTCAGTTTGAAATTGCAAAGGCTGTGCAAATTCGACCTTATAATGCTATAGCTTTTTCTGCTCCTATCTCTGTTTTCGTTTCTGTATTCTTAATTTATCCTTTAGGTCAATCAGGTTGGTTTTTTGCTCCTAGTTTTGGAGTAGCAGCAATTTTTAGATTTATTTTATTTTTTCAAGGTTTTCATAATTGGACTCTTAATCCTTTTCATATGATGGGAGTTGCTGGTGTTTTAGGTGCTGCTTTGTTATGTGCTATTCATGGTGCAACGGTAGAAAATACTATTTTCGAAGATGGGGATAGTCCAAATACATTTAGAGCATTTAATCCACTTCAATCTGAAGAAACTTATTCTATGGTGACAGCTAATCGTTTTTGGTCTCAAATTTTTGGTGTAGCTTTTTCAAATAAGCGCTGGTTGCATTTTTTTATGGTATTTGTTCCTGTAACAGGATTGAGGATGAGTGCTTTGGGTATTGTTGGATTAGCTCTTAATTTACGTGCTTATGATTTCGTTTCTCAAGAAATCCGTGCTGCAGAAGATCCAGAATTTGAAACTTTTTATACTAAAAATATTCTTTTAAATGAAGGTATTAGAGCTTGGATGGCTGCACAAGATCAGCCTCATGAACAATTTATATTCCCGGAGGAGGTTCTTCCACGTGGAAACGCTCTTTAA**

**>T. vulcanus:NIES2134_120090 K02706 photosystem II P680 reaction center D2 protein [EC:1.10.3.9] | (GenBank) psbD1_2; photosystem II reaction center D2 protein (N)**

**atgacgatcgcgattggacgagcgccagcggaacggggatggtttgacatcctcgacgactggctcaaacgggacagatttgtctttgtcggctggtcaggcatcctgcttttcccctgcgcctacctggcgctggggggctggctgaccggtaccacctttgtgacctcctggtacacccacggcctggcctccagctacctagaagggtgcaacttcctgaccgttgccgtttccacccccgccaacagcatgggacactccctgctcctcctgtggggacccgaagcccaaggggactttacccgctggtgccaactgggtggtctgtggacctttatcgccctccacggcgccttcggtctcattgggttcatgctgcggcagtttgaaattgcccgcttggtgggcgtccgtccctacaacgccattgccttcagcgcccccattgccgtctttgtcagcgtcttcttgatctatcccttggggcaatccagctggttctttgcccccagctttggcgtcgccgccatcttccgtttcctgctctttttccaagggttccacaactggaccttgaaccccttccacatgatgggggtagccggtgtgctagggggtgccctgttgtgtgccatccacggcgccacggtggaaaataccctcttccaagatggagaaggggcgagcaccttccgtgccttcaatcccacccaagcggaagagacctactcgatggtgacggcgaaccgtttttggagccaaatttttgggattgccttctcgaacaagcgctggttgcactttttcatgttgtttgtgccggtgacggggctgtggatgagtgcgattggcgtggtgggtctagcgttgaacctgcggtcctatgacttcatttcgcaggagattcgggctgcggaagaccctgaatttgagacgttctacacgaagaacctgctgttgaacgagggcatccgtgcttggatggcgccccaggaccaaccccatgaaaactttgtcttcccagaagaggtactcccccgtggtaacgctctgtag**

**>S. tuberosum:4099961 K02706 photosystem II P680 reaction center D2 protein [EC:1.10.3.9] | (RefSeq) psbD, SotuCp016; photosystem II protein D2 (N)**

**atgactatagccattggtaagtttaccaaagacgaaaatgatttatttgatattatggatgactggttacggagggaccgtttcgtttttgtaggctggtccggtctattgctctttccttgtgcctatttcgctgtagggggttggttcacaggtacaacctttgtaacttcatggtatacccatggattggccagttcttatttggaaggctgcaatttcttaactgccgcggtttctactcctgctaatagtttagcacattcgttgttgttactatggggtcctgaagcacaaggagattttactcgttggtgtcaattggggggtctgtggacttttgttgctctccatggagcttttggcctaataggtttcatgttacgtcaattcgagcttgctcgatctgttcaattgagaccttataatgcaatcgcattctctggtccaattgctgtttttgtttctgtatttctgatttatccactaggtcagtctggttggttctttgcacctagttttggtgtagcagctatatttcgattcatcctattttttcaagggtttcataattggaccttgaacccctttcatatgatgggagttgccggtgtattgggcgctgctttgctatgcgccattcatggtgctaccgtagaaaatactttatttgaagacggtgatggtgcaaatacattccgtgcttttaacccaactcaagccgaagaaacttattcaatggtcaccgctaaccgcttttggtcccaaatctttggggttgctttttccaataaacgttggttacatttctttatgttatttgtaccagtaaccggtttatggatgagtgctcttggagtagtcggtctagctctgaacctacgtgcctatgacttcgtttctcaggaaattcgcgcagcggaagatcctgaatttgagactttctacaccaaaaatattctcttaaacgaaggtattcgcgcttggatggcggctcaagatcagcctcatgaaaaccttatattccctgaggaggttctaccacgtggaaacgctctttaa**

**>S. lycopersicum:3950483 K02706 photosystem II P680 reaction center D2 protein [EC:1.10.3.9] | (RefSeq) psbD, LyesC2p074; photosystem II protein D2 (N)**

**atgactatagccattggtaagtttaccaaagacgaaaatgatttatttgatattatggatgactggttacggagggaccgtttcgtttttgtaggctggtccggtctattgctctttccttgtgcctatttcgctgtagggggttggttcacaggtacaacctttgtaacttcatggtatacccatggattggccagttcttatttggaaggctgcaatttcttaactgccgcggtttctactcctgctaatagtttagcacattcgttgttgttactatggggtcctgaagcacaaggagattttactcgttggtgtcaattggggggtctgtggacttttgttgctctccatggagcttttggcctaataggtttcatgttacgtcaattcgagcttgctcgatctgttcaattgagaccttataatgcaatcgcattctctggtccaattgctgtttttgtttctgtatttctgatttatccactaggtcagtctggttggttctttgcacctagttttggtgtagcagctatatttcgattcatcctattttttcaagggtttcataattggaccttgaacccctttcatatgatgggagttgccggtgtattgggggctgctttgctatgcgccattcatggtgctaccgtagaaaatactttatttgaagacggtgatggtgcaaatacattccgtgcttttaacccaactcaagccgaagaaacttattcaatggtcaccgctaaccgcttttggtcccaaatctttggggttgctttttccaataaacgttggttacatttctttatgttatttgtaccagtgaccggtttatggatgagtgctcttggagtagtcggtctagctctgaacctacgtgcctatgacttcgtttctcaggaaattcgcgcagcggaagatcctgaatttgagactttctacaccaaaaatattctcttaaacgaaggtattcgcgcttggatggcggctcaagatcagcctcatgaaaaccttatattccctgaggaggttctaccacgtggaaacgctctttaa**

**>M27309.1:481..1542, Pisum sativum, D2 photosystem II**

**atgactatagctcttggtaaatttaccaaagatcaaaatgatttatttgatattatggatgactggttacggagggaccgttttgtttttgtgggttggtccggtctattgctctttccttgcgcctattttgccgtggggggttggttcacaggtaccacctttgtaacttcatggtatactcatggattggcaagttcctatttggaaggttgtaacttcttaactgcagcagtctctactcctgctaatagtttagcacactctttgttgttactatggggtcctgaagcacagggagatttgacccgttggtgtcaattaggtggtctgtggacttttgttgctcttcacggtgctttcggattaataggttttatgttacgtcaatttgaacttgctcgatctgttcaattgcggccttataatgcaatcgcattctccggtccaattgctgtttttgtttctgtattccttatttatccactgggccagtctggttggttctttgcgcctagttttggtgtagcagctatatttcgattcattctctttttccaagggtttcataattggacattaaacccatttcatatgatgggagttgctggtgtattgggcgctgccctactatgcgctattcatggcgctattgtagaaaataccttatttgaagatggtgatggcgcaaatacattccgggcttttaacccaacccaagcagaagaaacttattcaatggttactgctaaccgcttttggtcccaaatctttggggttgctttttccaataaacgttggttatatttctttatgttatttgtaccagtaactggtttatggatgagtgctcttggagtagtcggtctggccctgaacctacgtgcctatgactttgtttctcaagaaatccgcgcagcggaagatcctgaatttgagactttctacaccaaaaatattctcttaaacgaaggtattcgtgcgtggatggcgactcaagatcagcctcatgaaaaccttatattccctgaggaggttctaccacgtggaaacgctctttaa**

**>N. tabacum:800525 K02706 photosystem II P680 reaction center D2 protein [EC:1.10.3.9] | (RefSeq) psbD, NitaCp015; photosystem II protein D2 (N)**

**atgactatagcccttggtaagtttaccaaagacgaaaatgatttatttgatattatggatgactggttacggagggaccgtttcgtttttgtaggctggtccggtctattgctctttccttgtgcctatttcgctgtagggggttggttcacaggtacaacctttgtaacttcatggtatacccatggattggccagttcttatttggaaggctgcaatttcttaactgccgcggtttctactcctgctaatagtttagcacattcgttgttgttactatggggtcctgaagcacaaggagattttactcgttggtgtcaattggggggtctgtggacttttgttgctctccatggagcttttggcctaataggtttcatgttacgtcaattcgagcttgctcgatctgttcaattgagaccttataatgcaatcgcattctctggtccaattgctgtttttgtttctgtatttctgatttatccactgggtcagtctggttggttctttgcacctagttttggtgtagcagctatatttcgattcatcctcttttttcaagggtttcataattggacgttgaacccatttcatatgatgggagttgccggtgtattgggcgctgctttgctatgcgccattcatggtgctaccgtagaaaatactttatttgaagacggtgatggtgcaaatacattccgtgcttttaacccaactcaagccgaagaaacttattcaatggtcaccgctaaccgcttttggtcccaaatctttggggttgctttttccaataaacgttggttacatttctttatgttatttgtaccagtaaccggtttatggatgagtgctcttggagtagtcggtctagccctgaacctacgtgcctatgacttcgtttctcaggaaattcgcgcagcggaagatcctgaatttgagactttctacaccaaaaatattctcttaaacgaaggtattcgcgcttggatggcggctcaagatcagcctcatgaaaaccttatattccctgaggaggttctaccacgtggaaacgctctttaa**

**>T. aestivum, photosystem II protein D2, Sequence 1062 bp**

**atgactatagcccttggtagaattcctaaagaagaaaatgatctatttgatactatggatgactggttacgaagggaccgtttcgtttttgtaggatggtctggcctattgctctttccttgtgcttatttcgctttagggggttggtttacagggacaacttttgtaacttcttggtatacccatggattggctagttcctatttggaaggttgtaatttcttaaccgcagcagtttctacccctgccaatagtttagcacactctttgttgctactatgggggcccgaagcacaaggagattttactcgttggtgtcaattaggcggtctatggacttttgtagctctccacggggcttttgcactaataggtttcatgttacgccaatttgaacttgctcggtctgttcaattgcggccttataatgcaatctcattctctggtccaattgctgtttttgtttctgtattccttatttatccactggggcaatctggttggttctttgcgccgagttttggcgtagcagcgatatttcgattcatccttttctttcaaggatttcataattggacgttgaacccatttcatatgatgggagttgccggagtattaggtgcggctctgctatgcgctattcatggagcgaccgtagaaaacactctatttgaggacggtgatggtgcaaataccttccgtgcttttaacccaactcaagctgaagaaacttattcaatggtcactgctaaccgcttttggtcccaaatctttggtgttgctttttccaataaacgttggttacatttctttatgctatttgtacccgtcaccggtttatggatgagtgctattggcgtagttggcttggctctgaacttacgtgcctatgactttgtttcccaggaaatccgtgcagcggaagatcctgaatttgagactttctacaccaaaaatattcttttaaacgagggtattcgtgcgtggatggcagctcaggatcagcctcatgaaaatcttatattccctgaggaggttctaccacgtggaaacgctctttaa**

**>Nostoc sp. PCC 7120:alr4290 K02706 photosystem II P680 reaction center D2 protein [EC:1.10.3.9] | (GenBank) psbD; photosystem II protein D2 (N)**

**atgaccatcgcagtaggacgggcccccagtagagggtggtttgacgtactagacgactggttaaagcgcgatcgcttcgtattcgtaggctggtcagggatattattatttccttgcgccttcctagcactaggcggttggctaaccggtacaacatttgtaacgtcgtggtacacccacggactagcatcatcctacctggaaggagcaaacttcctgacagtagcagtatccagcccagcagacagcatgggacactccctgttgttgttatgggggccagaagcccaaggcgacttgacccgttggtttcagctaggagggttatggccattcgtagccctacacggagcctttggattaatcgggttcatgctacgtcaatttgaaattgcgcggctagtaggaattagaccctacaacgcactagcattttcagcccccatcgcggtattcgtcagcgtattcctgatgtaccccttgggacaatcttcctggttcttcgcacccagcttcggagtagcagcaatcttcagattcttgttattcctccaagggttccacaactggacactcaaccccttccacatgatgggagtagctggtgtactaggtggagcgttactgtgtgccatccacggtgccacagtagaaaacaccttgtttgaagacggcgaaggcgcaaacaccttccgtgccttcaaccccacccaatcagaagaaacctattcaatggtgacagccaaccgattctggtcacagatattcgggattgcgttctccaacaaacgctggttgcacttcttcatgttgttcgtacccgtaactgggttgtggatgagtgctgtaggcatcgtcggtttagcattaaacctccgggcttatgacttcgtttcccaagaactacgggcagcagaagacccagaatttgaaaccttctataccaagaacattttgttgaacgagggtatccgcgcttggatggctcctcaagatcagcctcacgaaaaatttgtattccccgaagaggtactaccacgtggtaacgctctctaa**

**>C. reinhardtii:ChreCp064 K02706 photosystem II P680 reaction center D2 protein [EC:1.10.3.9] | (RefSeq) psbD; photosystem II protein D2 (N)**

**atgacaattgcgatcggtacatatcaagagaaacgcacatggttcgatgacgctgatgactggcttcgtcaagaccgtttcgtattcgtaggttggtcaggtttattactattcccttgtgcttactttgcattaggtggttggttaactggtactactttcgttacttcatggtatacgcatggtttagctacttcttacttagaaggttgtaacttcttaacagcagctgtttctacacctgctaacagtatggctcactctcttctatttgtttggggtccagaagctcaaggtgatttcactcgttggtgtcaacttggtggtttatgggcattcgttgctttacacggtgcatttggtttaattggtttcatgcttcgtcagtttgaaattgctcgttcagtaaacttacgtccatacaacgcaattgctttctcagcaccaattgctgtattcgtttcagtattcctaatttacccattaggtcaatcaggttggttctttgcacctagtttcggtgtagctgctatcttccgtttcattttattcttccaaggtttccacaactggacacttaacccattccacatgatgggtgttgctggtgttttaggtgctgctttattatgtgctattcacggtgctactgttgaaaacacattattcgaagacggtgacggtgctaacacattccgtgcattcaaccctacacaggctgaagaaacatactctatggttactgctaaccgtttctggtcacaaatcttcggtgttgctttctctaacaaacgttggcttcacttcttcatgttattagttccagtaactggtctttggatgagtgctattggtgttgtaggtttagctctaaacttacgtgcttacgacttcgtatcacaagagattcgtgctgctgaagaccctgaattcgaaacattctacactaaaaacattcttcttaacgaaggtattcgtgcttggatggctgctcaagaccaaccacacgaacgtttagtattccctgaagaagtattaccacgtggtaacgctctataa**

**>V. carteri, photosystem II protein D2, Sequence 1059 bp**

**atgacaatagcgattggtacatatcaagaaaaacgcacatggtttgatgacgctgacgactggcttcgtcaagaccgtttcgtattcgttggttggtcgggtttattacttttcccttgtgcttactttgcattaggtggttggttaactggtacaactttcgttacatcatggtacacacatggtctagctagttcttacctagaaggttgtaacttcttaacagcagctgtatcaacaccagcaaacagtatggctcactctcttctatttgtttggggtccagaagctcaaggtgatttcactcgttggtgtcaacttggaggtttatggacatttgtagctttacacggggcgtttggtctaattggctttatgctacgtcaatttgaaattgctcgttcagtaaacttacgtccatacaacgctattgctttctcagcaccaattgctgtattcgtatctgttttcttaatttacccattaggtcaatcaggttggttttttgcacctagttttggtgttgctgctatcttccgtttcatcttatttttccaaggttttcataactggacgttaaatccattccacatgatgggtgttgctggtgttctaggtgctgcgttattatgtgctattcacggtgctactgtagaaaacacattattcgaagatggtgatggtgcgaacactttccgtgctttcaacccaacacaggctgaagaaacatactctatggtaacagcaaaccgtttctggtctcagatctttggtgtggctttctctaacaaacgttggttacacttcttcatgttattagttcctgtaacaggattatggatgagtgcacttggagttgtaggccttgcattaaatttacgtgcttacgacttcgtttcacaagaaattcgtgcagccgaagatcctgaatttgaaactttctatactaaaaacattcttcttaacgaagggatccgtgcttggatggctgctcaagaccaaccacatgaacgtttagtgttcccagaagaagttttaccacgtggtaacgctctttaa**

**>Crocosphaera subtropica ATCC 51142:cce_0660 K02706 photosystem II P680 reaction center D2 protein [EC:1.10.3.9] | (GenBank) psbD1; photosystem II D2 protein (N)**

**ttggattctatgactattgcagtcggacgcgcaccagaaagaggatggtttgatgtcctcgatgactggttaaaacgcgatcgctttgtattcgtcggttggtctgggttactactatttccctgtgcctacctcgctctcggtggctggttaaccggaaccacctttgttacctcctggtacacccacggcttagccagttcctacctagaaggatgtaacttcctaaccgtagcggtttcttcccccgccaacgccttcggacactccttactgttcctttggggaccagaagcccaaggagactttacccgttggtgtcaaatcggtggtttatggacatttaccgcccttcacggagcattcggactgattggcttcatgttacgtcagtttgaaattgcccgtctggtaggtattcgtccttacaacgccatcgccttctctgcgcctattgcggtgttcgtcagtgtattcttgatgtaccctctaggacaatcaagttggttctttggacctagctttggtgtcgccggaattttccgtttcattctcttcctacaaggattccacaactggacattgaaccccttccacatgatgggagtcgcaggtgttctcggtggtgctttactgtgtgccattcatggtgcaacggtagaaaacaccttgtttgaagacggtgaacaagctaacactttccgtgcctttgaacccacccaagcagaagaaacctactcaatggtgaccgcaaaccgtttctggtcacagattttcggaattgccttctccaacaaacgttggttacacttcttcatgttattcgtccccgtaactgggttatggatgagtgcgatcggtattgtcggtttagctttaaacttacgagcttatgactttgtgtctcaagaattacgggcagcagaagaccctgaatttgagacattctacaccaaaaacattttattaaacgaaggtctgcgagcttggatggctccccaagaccaaccccatcaaaactttgtattccctgaggaggtactgccccgtggtaacgctctctaa**

**>Gloeobacter violaceus PCC 7421:gvip318 K02706 photosystem II P680 reaction center D2 protein [EC:1.10.3.9] | (RefSeq) psbD; photosystem II protein D2 (N)**

**atgaccatcgctgtaggaagaagtgagcagagccaagggtggttcgacgcccttgacgactggctcaagaaggaccgcttcgttttcatcggttggtccggcctgctgttcttccccactgcttacctcgccgtgggtgcctggctgaccggcacgaccttcgtcacctcctggtttacccatgggctcgccagctcctaccttgaaggtggcaacttcctgaccgtcgccgtcagctcgcctgccgacgcgatgggtcactcgctgcttctgctgtggggtcctgaagcccagggtgactttacccgctggtgccagattggcgggctttgggcgtttatctcctttcacggcgctctggcgttgatgggcttcatgctccgccagttcgaaattgctcgcctgatcggcatccgtccttacaacgcgatcgccttctcggctccgatcgccgtgttcgtttcggtcttcttgatgtacccgctgggccagcactcctggttcttcggccccagctacggcgtcaacggcatcttccgcttcttgctgttcttccagggcttccacaactggacgctgaaccccttccacatgatgggtgtggcgggtgtgctcggtggtgccctgctgtgcgccatccacggcgccacggtcgagaacaccctgtttgaagacggcgaagcgcccaacaccttcaaggccttcgaccccgcccaagaagaagagacctactcgatgatcctggcaaaccgcttctggagccagatcttcggtatcgccttctccaacaagcgctggctgcacttcttcatgctgttcgtgcccgtcaccggtctgtggatggcttccatcggcattatcggtgtggccctgaacctgcgggcttacgagttcgtttcccaggaaatccgcgctgcgcaggatcctgagtttgagactctctacaccgcgaacattctgatcaacgaaggtatccgcgcctggatgggtccctacgaccagaactacgacgagacgctcaagttccctgaagaggtgctgccccgtggaaacgcgctttga**

**>Geitlerinema sp. PCC 7407:GEI7407_0926 K02706 photosystem II P680 reaction center D2 protein [EC:1.10.3.9] | (GenBank) photosystem II D2 protein (photosystem q(a) protein) (N)**

**atgactatagcagtcggacgcgcacaggcccagcgaggatggtttgacgttctcgacgactggctgaagcgcgatcggtttgtgttcattgggtggtctggtttgctgctgttcccctgcgcctacttggcggttgggggatggctgacggggaccaccttcgtaagctcgtggtacacccacgggttggcatcgtcctacctggaaggatgcaactttttgaccgtggcggtgtcgacaccgcccaacagcctgggacactccctgctgtttttgtggggacctgaggcccaaggggactttgtccgctggtgccaactgggaggtctgtggacctttgtcgccctgcacggagctttcgggttgatcgggttcatgctgcgtcagtttgagatttctcgactggtgggcattcgtccgtacaacgcgatcgccttcagcgcgccgattgcagtgtttgtgtcggtgtttctgatgtacccgctgggtcagtctggctggttttttgcaccgagctttggtgtagcggcgattttccgctttctgctgttcttccaaggtttccacaactggacgttgaaccccttccacatgatgggagtggctggcgttctggggggtgcactgctgtgcgcgatccacggagcgacggtggagaacacgctgtttgaggacggcgagaacgcgaacaccttccgggcgttcaacccgacgcaggcagaagagacgtactcgatggtgacggcgaaccgtttctggtcgcagatcttcgggattgcgttttcgaacaagcgctggctgcacttcttcatgctgttcgtgcctgtgacgggcctgtggatgagctcggtgggtatcattggtttgggtctgaacctgcgggcatacgactttgtttcgcaggagattcgggcagcggaggaccctgagttcgagacgttctacacgaagaacatcttgctgaacgagggtatccgggcctggttggcaccgcaggaccagccgcacgaaggatttgctttccctgaggaggtactgccccgtggtaacgctctctaa**

**>P. trichocarpa:4929654 K02706 photosystem II P680 reaction center D2 protein [EC:1.10.3.9] | (RefSeq) psbD, Poptr_cp016; photosystem II protein D2 (N)**

**atgactatagcacttggtaaatttaccaaagatgaaaatgatttatttgatattatggatgactggttacggagggaccgtttcgtttttgtgggttggtccggtctattgctctttccttgtgcctattttgccttagggggttggttcacaggtacaacctttgtaacctcatggtatacccatggattggccagttcctatttggaaggttgcaacttcttaaccgccgcagtttctactcctgctaatagtttagcccattctttattattactatggggtcctgaagcacaaggagattttactcgttggtgtcaattaggtggcttgtggacttttgttgctctccacggagctttcggactaataggttttatgttacgtcaatttgaacttgctcgatccgtgcaattacgaccttataatgcaatcgcattctctggtccaattgcggtttttgtttctgtattcctgatttatccactaggtcagtctggttggttctttgcgcctagttttggcgtagcagctatatttcgattcatcctctttttccaaggatttcataattggacactgaacccatttcatatgatgggagttgccggtgtattgggcgctgctctgctatgcgctattcatggtgctactgtagaaaatactttatttgaagatggtgatggtgcaaatacattccgtgcttttaacccaactcaagctgaagaaacttattccatggtcaccgccaaccgcttttggtcccaaatttttggggttgctttttccaataaacgttggttacatttctttatgttatttgtaccagtaaccggtttatggatgagtgctcttggagtagttggtctggctctgaacctacgtgcctatgacttcgtttctcaggaaatccgtgcagcggaagatcctgaatttgagactttctataccaaaaatattcttttaaatgaaggcattcgtgcttggatggcggctcaagatcagcctcatgaaaaccttatattccctgaggaggttctaccccgtggaaacgctctttaa**

**>Gloeobacter kilaueensis JS1:GKIL_2488 K02706 photosystem II P680 reaction center D2 protein [EC:1.10.3.9] | (GenBank) psbD; photosystem II protein D2 (N)**

**atgaccattgctgtaggaagaagtgagcagagtcagggctggtttgacgccctcgacgactggctcaagaaggaccgctttgtatttatcggttggtccgggctgcttttcttccccaccgcatatttcgccgtgggtggctggctgaccggaaccactttcgtttcctcctggtatacccatggtctggccagctcttacctggagggtgctaactttctgacctccgccgtcagctcgcctgccgacgcgatgggtcactcgctacttttgctgtggggtcctgaggctcaaggggacttcacgcgctggtgccagattggcggcctgtgggcgttcattgccttccatggtgcgctggcgctcatgggctttatgctccgccagttcgagattgcccgtttgattggcattcgcccttataacgccattgccttctcagctcccatcgccgtattcgtctcggtgttcttgatgtatccgttgggccagcactcctggttcttcggtccgagctttggtgtcaatggcatcttccgctttctgctctttgtacagggcttccacaactggacgctcaaccccttccacatgatgggtgtggcaggtgtgctgggcggtgcattgttgtgcgccattcacggtgccacggtcgagaataccctctttgaagacggcgaagcgccgaacaccttcaaggctttcgacccggctcaagaagaagaaacctactcgatggtgctcgccaaccgcttctggagccagatcttcggcattgccttctccaacaagcgctggctgcacttctttatgctgttcgtgccggtgacgggcctgtggatggcctcgatcggcttcgtcggtctggccttgaacctgcgcgcctacgagttcatctctcaggagagccgcgctgctcaagatccggagtttgaaactctctacactgcgaacattctgatcaacgaaggcatccgcgcctggatgggtccctacgaccagaactacgacgaaaccctgaagttccctgaagaggtgctgccccgtggaaacgcgctttga**

**>Microcystis aeruginosa, Sequence 1056 BP; Photosystem II D2 protein**

**atgaccattgctgtcggacgcgccccagaaagagggctgtttgatgctctcgatgactggctcaaaagagaccgtttcgtcttcatcggttggtctggtttactactcttcccctgcgccttcatggccctaggtggatggttaaccggcaccaccttcgtcacctcctggtacacccacgggttagccagttcctacttagaaggcggcaacttcctgactgtagccgtctccacccccgccgatgccttcggtcactccatcctctttctctggggaccggaagcccaaggtaacttcacccgttggtgtcaaatcggcggtttatggccctttgtcgctctccacggtgctttcggcttgattggcttcatgctacgtcagtttgaaatcgcccgtttagtcggcattcgtccctacaacgccctcgccttctctggcccgattgcggtgttcgtcagtgtcttcctgatgtaccccctcggtcagtctagctggttctttgcccctagcttcggcgtggctggtatcttccgttttattctcttcttccaaggcttccacaactggacccttaaccccttccacatgatgggtgtagctggtatcctcggtggtgcgcttctctgtgctattcacggagcgaccgtagaaaataccctgtttgaagacggtgaaggttccaacactttccgagcttttgaacccacccaagcggaagaaacctactccatggtcactgcgaaccgtttctggtcgcaaatcttcggcatcgctttctccaacaaacgttggttacacttcttcatgctctttgtccccgtgactggtttatggatgagtgctgtgggtgtagtgggattagcccttaatctacgggcctatgacttcgtttctcaggaattgagagcggccgaagacccggaatttgaaaccttctacactaaaaatattctgcttaacgaaggtctgagagcttggatggctccccaagaccaaccccacgaaaactttatcttccctgaggaggttctcccgcgtggtaacgctctctaa**

**>Oscillatoria acuminata PCC 6304:Oscil6304_1899 K02706 photosystem II P680 reaction center D2 protein [EC:1.10.3.9] | (GenBank) Photosystem II DII subunit, Q(A) protein (N)**

**atgacgattgcagtcggacgcgcacaggcagagcggggatggtttgacgtcctcgacgactggctaaaacgcgatcgctttgtatttatcggttggtctggcctattactcttcccctgcgcctacctcgcagtggggggctggctgaccggcaccaccttcgtcacctcttggtacacccacggattagcttcctcctatttagaaggttgcaacttcctcacggtagccgtttcaactccccccaacagtctgggccattccctgctgttcctctggggtcctgaagcccaaggcgacttcactcggtggtttcaactcggcggtctgtggacctttgtcgccctgcatggggcctttggtctgattggcttctgcctgcgtcagttggaaattgcccgcctgttagggattcgtccctacaacggactcgcctttaccggacccattgcggtgttcgtcagcgtgttcttgatttaccccttgggtcaatcgggctggttctttgctccgagctttggcgttgctgctatcttccgattcctgttgttcttccaagggttccataactggaccctcaaccccttccacatgatgggagtggcgggtatcctgggcggtgcactgctgtgcgccattcatggagccaccgtggaaaacaccttgtttgaagatggtgaaggttccaacaccttccgcgcctttgaaccaactcaggcggaagaaacctactcgatggttaccgccaaccgtttctggtctcaaatttttgggattgccttctccaacaagcgctggctgcacttctttatgttgtttgtgccggtaacaggcttgtggatgagtgcgatcggggtcgtgggcttaggtttaaacctgcgtgcttatgacttcgtgtctcaagaactgcgggctgcggaagacccagaatttgaaaccttctacactaagaatattcttctgaatgaagggattcgggcttggatggctccggttgaccagcctcacgaaaacttcgagttccctgaagaagttctacctcgcggtaacgctctgtag**

**>Nostoc sp. ATCC 29411 / PCC 7524:Nos7524_0461 K02706 photosystem II P680 reaction center D2 protein [EC:1.10.3.9] | (GenBank) Photosystem II DII subunit, Q(A) protein (N)**

**atgaccatcgcagtaggacgcgcccccagtagagggtggtttgacgtactagacgactggttgaagcgcgatcgcttcgtattcgtaggttggtcagggatattattattcccctgcgccttcctcgcactaggcggttggctcaccggcacaaccttcgtcacctcctggtacacccacggactagcctcatcctacctagaaggctgtaacttcttgacagtagcagtatctagccccgccgacagcatgggacactccctgttgttgctgtggggaccagaagcccaaggggacttcacccgttggtgtcaactaggtggattatggcccttcgtagccctacacggagcctttggtttaatcggcttcatgttgcggcaatttgagattgcgcggttagtaggcatccgtccctacaacgccctagcattctcagcccccatcgcggtattcgtcagcgtgttcttgatgtaccccttgggacaatcctcctggttcttcgcacccagcttcggtgtagcagccattttccggttcttgttattcctgcaaggtttccacaactggacactcaaccccttccacatgatgggtgtagcgggtgtactcggtggggcgctattgtgtgcgattcacggtgccaccgtagaaaacaccctatttgaagacggcgacggtgctaacaccttccgcgccttcaaccccacccaagcagaagaaacctattccatggtgacagcaaaccgtttctggtcacagattttcgggattgctttctccaacaaacgctggttgcacttcttcatgttgttcgtaccagtaactggtttgtggatgagtgctgtcggtattgtcggtttagcactcaacctgcgggcgtatgacttcgtatcgcaagaattgcgtgcggcagaagaccccgaatttgaaaccttctataccaaaaacattttgttgaacgagggtatccgcgcttggatggctcctcaagaccaaccccacgaaaaatttgtcttccccgaagaggtattacctcgcggtaacgcactgtaa**

**>Prochlorococcus marinus (MIT 9301):P9301_13491 K02706 photosystem II P680 reaction center D2 protein [EC:1.10.3.9] | (GenBank) psbD; Photosystem II PsbD protein (D2) (N)**

**atgacgatcgcagttggtagcgccccacaaagaggatggtttgatgtcctcgatgattggttgaagcgcgaccgctttgtatttattggttggtccggactacttctacttccttgtgcataccttgctataggtggttggtttgtcggaacaacatttgttacctcttggtacacacacggagttgcaagttcataccttgaaggttgtaacttcttaacagcagctgtaagcacccctggtgatgccatgggacacagtcttctatttttatggggtcctgaagcccaaggtagtttcgtaagatggctacaacttggtggtctttggaacttcgttgcattacatggagtatttggcctaattggttttatgcttcgtcagtttgaaattgctggccttgttggaattagaccatacaacgcactagctttctcagcagtaattgcagtattcacaagtattttccttatttatcctttaggacagcatagttggttcttcgcaccttcattcggtgttgcagcaatcttccgttacatcctattcattcaaggttttcacaatatcactttaaacccattccatatgatgggagttgctggaattcttggtggtgctctactttgcgctattcatggagctacagttcaaaatactttgtatgaagatacaagtatttacacagatggtaaggttcaaagttcaacatttagagcttttgatccaactcaagaagaagaaacctattcaatgattacagcgaatagattttggagtcaaatcttcggtattgctttctcaaacaagcgtttcttacatttcttgatgctatttgtacctgttatgggtatgtggacatcttctattggtattgtcggcttagcactaaacttgagagcttatgacttcgtaagccaagaaattcgtgcagcagaagatccagaatttgaaactttctatacaaaaaatatacttttgaacgaaggtatgcgagcatggatgtcttctgtggatcaaccacacgaaaactttgtattccctgaggaggttcttccacgtggaaacgccctttaa**

**>Physcomitrella patens:2546710 K02706 photosystem II P680 reaction center D2 protein [EC:1.10.3.9] | (RefSeq) psbD, PhpapaCp044; photosystem II protein D2 (N)**

**atgactatagccattggaaagtcttccaaagaaccaaaaggtttatttgatagtatggatgactggctaagaagagaccgttttgtatttgtaggttggtctggtctattactttttccgtgcgcttatttttctttaggtggatggtttacaggtacaacttttgtaacttcatggtatactcatggattggctagctcttatttagaaggttgtaattttctaactgctgctgtttctactcctgctaatagtttagcacattcgttattactattatggggtccagaagcacaaggagatttcactcgttggtgtcaattaggaggtctatggactttcgttgctcttcatggagcttttgctttaataggctttatgttgcgccaatttgagcttgctagatccgtacaattacgtccttataacgcaattgctttttctggcccaattgctgtttttgtttctgtatttttaatttacccacttggtcaatcaggttggttttttgcacctagttttggtgttgcagcaattttccgattcattttattcttccaaggtttccataactggactttaaacccatttcatatgatgggagttgctggagtcttgggagctgctcttttatgtgctattcatggtgcaactgtagaaaatactttatttgaagatggtgatggtgcaaatacattccgtgcgtttaacccaactcaatctgaagaaacttattctatggttacagctaaccgtttctggtctcaaatcttcggtgttgcattttctaacaaacgttggttgcatttctttatgctatttgtaccagtaactggtttatggatgagtgctattggagtagttggtttagctttaaatttacgagcttatgattttgtt**

**tctcaggaaattcgtgcagctgaagaccctgaatttgaaactttttacactaaaaatatt**

**cttctaaatgaaggtattcgtgcttggatggcagctcaagatcagcctcatgaaaatctt**

**gtattcccagaggaggttctaccacgtggaaacgctctttaa**

**>G. sulphuraria:JL72_p002 K02706 photosystem II P680 reaction center D2 protein [EC:1.10.3.9] | (RefSeq) psbD; photosystem II protein D2 (N)**

**atgactatagctatagaaagaaatatacaaagagggtggttcgacttagttgatgattggctaaaacgtgatcgatttgtctttattggatggtcaggtttgctactttttccatgttcatatctagctctaggtgcctggtttacaggtacaacttttgttacttcttggtatacgcatggtttagcttcttcttatttagaaggatgtaattttttaaccgctgcagtatcaagtcctgcaaatagtatgggacactcattattatttttatggggaccagaagctcaaggtgattttactagatggtgtcaaattggaggtctttggacttttactgctttacatggtgcttttgccttgataggtttttgtttgcgtcaatttgaaatagccagattagtcggaattaggccatacaatgcaatagctttttcaggaccaattgcagtttttgtttcagtatttcttatttatccacttgggcaagctagttggttttttgctcctagttttggagttgcagctatctttcgatttattttatttttacaaggtttccataattggactttaaatccatttcatatgatgggtgttgctggaatactcggaggagcattactttgtgctattcatggagcgactgtagagaatacattattcgaagatggcgaagctgctaatactttcagggcttttacaccaacacaatcagaggaaacttattcaatggttacggctaatcgtttttggtcacaaatttttggtgttgcattttctaacaaaagatggctacatttttttatgctatttgtgccagttacaggattatggacaagctctattggtatcataggacttgcattaaatttacgtgcatatgattttgtctctcaagaattacgtgccgcagaggacccagaatttgagactttttatactaaaaatttacttctaaatgaaggtataagagcatggatggcaacacaagatcaaccacatgaaaattttgtattcccagaggaggttttaccacgtggaaatgccctttaa**

**>NC_011600.1:26125-27180 Vaucheria litorea chloroplast, photosystem II protein D2**

**ATGACCATAGCAATTGGACAAAATCAAGAACGAGGTATTTTCGATCTTATTGATGACTGGTTGAAACGAGATCGCTTCGTATTTATTGGTTGGTCAGGTCTTCTTTTATTTCCAACAGCTTATTTATCTTTAGGTGGTTGGTTTACAGGTACAACTTTCGTAACATCATGGTATACTCATGGTTTGGCTAGTTCATATTTAGAAGGTTGTAATTTTTTAACTGCTGCAGTTTCATCCCCTGCAAATAGTATGGGACATTCATTAATTTTACTTTGGGGTCCTGAAGCACAAGGTGATTTTACACGTTGGTGTCAAATTGGTGGATTATGGGCTTTTATTGCTTTACATGGTTCATTTGCATTAATTGGATTTTGTTTACGACAATTTGAAATTGCTCGTTTAGTTGGTATTCGTCCATATAATGCTATAGCTTTTTCTGGACCTATTTCAGTATTTTTATCAGTATTTTTATTATACCCTTTAGGACAAGCTAGTTGGTTTTTTGCACCTAGTTTTGGTGTTGCAGCAATTTTTCGTTTTCTATTGTTTTTACAAGGTTTTCATAATTGGACATTAAATCCATTTCATATGATGGGAGTAGCAGGAATTTTAGGTGGTGCTCTTTTATGTGCTATACATGGAGCAACTGTTGAAAATACATTATTTGAAGATGGTGATGCAGCAAATACTTTCCGTGCATTTACTCCAACACAATCTGAGGAAACTTATTCGATGGTTACAGCTAATCGTTTCTGGTCTCAAATTTTTGGTATAGCTTTTTCAAACAAACGTTGGTTACATTTCTTTATGCTATTTGTACCAGTTACTGGTCTATGGACAAGTTCAATTGGAATTGTAGGTTTAGCATTAAATTTACGTGCTTATGACTTTGTTTCTCAAGAACTTCGTGCTGCTGAAGATCCTGAATTTGAAACTTTTTATACAAAAAATATTTTATTAAATGAAGGTATCCGTGCTTGGATGGCAGCACAAGATCAACCACACGAAAACTTTGTATTCCCTGAGGAGGTTTTACCACGTGGAAACGCCCTTTAA**
